# Supplementary material for: Lassa virus glycoprotein nanoparticles elicit neutralizing antibody responses and protection
Source: Cell Host Microbe. 2022 Dec 14;30(12):1759–1772.e12. doi: 10.1016/j.chom.2022.10.018 (PMC9794196; doi:10.1016/j.chom.2022.10.018)
Supplement: Document S1. Figures S1–S5 and Tables S1–S5 [file mmc1.pdf]

Cell Host & Microbe, Volume *30*

Supplemental information

Lassa virus glycoprotein nanoparticles elicit neutralizing antibody responses and protection

Philip J.M. Brouwer, Aleksandar Antanasijevic, Adam J. Ronk, Helena Müller-Kräuter, Yasunori Watanabe, Mathieu Claireaux, Hailee R. Perrett, Tom P.L. Bijl, Marloes Grobben, Jeffrey C. Umotoy, Angela I. Schriek, Judith A. Burger, Khadija Tejjani, Nicole M. Lloyd, Thijs H. Steijaert, Marlies M. van Haaren, Kwinten Sliepen, Steven W. de Taeye, Marit J. van Gils, Max Crispin, Thomas Strecker, Alexander Bukreyev, Andrew B. Ward, and Rogier W. Sanders

Table S1. CryoEM data collection information, model building and refinement information, related to figure 3 and 5

	GPC-I53-50 nanoparticle	GPC-I53-50A + LAVA01 Fab		GPC	I53-50 nanoparticle (full assembly)	GPC-I53-50A + LAVA01 Fab
Microscope	Titan Krios	Titan Krios	Final map resolution (Å)	3.97	3.67	4.41
Voltage (kV)	300	300	EMDB ID	EMD-25107	EMD-25108	EMD-25109
Detector	Gatan K2 Summit	Gatan K2 Summit	Residues	1089	21060	1728
Recording mode	Counting	Counting	Amino-acids	1014	21060	1641
Magnification	29,000	29,000	Carbohydrates	75	0	87
Movie micrograph pixel size	1.03	1.03	RMSD Bond Length (4σ)	0.023	0.020	0.021
Dose rate ($e^-/Å^2/s$)	4.8	5.3	RMSD Bond Angles (4σ)	1.733	1.688	1.971
No. of frames per movie micrograph	42	38	Ramachandran			
Frame exposure time (ms)	250	250	Outliers (%)	0.0	0.0	0.0
Movie micrograph exposure time (s)	10.5	9.5	Allowed (%)	1.8	2.3	1.9
Total dose ($e^-/Å^2$)	50.4	50.3	Favored (%)	98.2	97.7	98.1
Grid Type	QuantiFoil R 2/1	UltrAuFoil R 1.2/1.3	Rotamer Outliers (%)	0.0	0.0	0.0
Under focus range (μm)	0.6 - 1.6	0.6 - 1.6	Clash Score	1.47	0.19	1.10
Number of movie micrographs	2,337	3,098	MolProbity Score	0.88	0.64	0.82
			EMRinger Score	2.57	2.81	1.37
			PDB ID	7SGD	7SGE	7SGF

Table S2. Midpoint pseudovirus neutralization titers from rabbits that received GPC-I53-50A or GPC-I53-50NPs, tested against a panel of LASV-pseudoviruses, related to figure 4

ID_{50} values, i.e. the serum dilution at which infectivity was inhibited by 50%, are shown and color coded: white = no neutralization, ID_{50} < 20; yellow = weak neutralization, 20 > ID_{50} >100; orange = moderate neutralization, 101 > ID_{50} > 1000; red = strong neutralization, ID_{50} > 1000. BG505 = HIV pseudovirus (negative control).

Rabbit immunization

		Week 0	Week 4	Week 6	Week 18		Week 30				
		Autologous	Autologous	Autologous	Control	Autologous	Control	Autologous	Heterologous		
	Strain	Josiah	Josiah	Josiah	BG505	Josiah	BG505	Josiah	NIG08-A41	LassaCSF	Bamba
	Lineage	IV	IV	IV	-	IV	-	IV	II	III	V
Immunogen	ID										
GPCysR4-I53-50A	187	<20	<20	<20	<20	48	<20	580	275	199	<20
	188	<20	<20	<20	<20	<20	<20	<20	<20	<20	<20
	189	<20	<20	<20	<20	1518	<20	2565	411	622	<20
	190	<20	<20	<20	<20	<20	<20	<20	<20	<20	<20
	191	<20	<20	<20	<20	<20	<20	<20	<20	<20	<20
	192	<20	<20	<20	<20	<20	<20	<20	<20	<20	<20
GPCysR4-I53-50NP	193	<20	<20	<20	<20	38	<20	51	30	24	<20
	194	<20	<20	<20	<20	623	<20	2749	1733	1151	41
	195	<20	<20	<20	<20	39	<20	93	45	42	41
	196	<20	<20	<20	<20	121	<20	413	42	52	37
	197	<20	<20	<20	<20	58	<20	50	26	26	<20
	198	<20	<20	<20	<20	<20	<20	<20	<20	21	<20

Table S3. Endpoint neutralization titers from rabbits that received GPC-I53-50A or GPC-I53-50NPs, tested against authentic LASV, related to figure 4

The virus neutralization titer is calculated as the geometric mean titers (GMT) of the reciprocal value of the last serum dilution at which inhibition of the cytopathic effect on infected Vero E6 cells is detectable. The initial dilution was 1:16 so a titer of 8 was noted when no inhibition was observed.

Rabbit immunization

		Week 30
		Autologous
	Strain	Josiah
	Clade	IV
Immunogen	ID	
GPCysR4-I53-50A	187	38
	188	8
	189	23
	190	8
	191	8
	192	8
GPCysR4-I53-50NP	193	8
	194	32
	195	11
	196	8
	197	11
	198	8

Table S4. Midpoint pseudovirus neutralization titers from guinea pigs that received GPC-I53-50NPs, tested against autologous LASV-pseudovirus, related to figure 4
ID_{50} values, i.e. the purified IgG dilution at which infectivity was inhibited by 50%, are shown and color coded: white = no neutralization, ID_{50} < 20.

Guinea pig immunization

		Week 18
		Autologous
	Strain	Josiah
	Clade	IV
Immunogen	ID	
GPCysR4-I53-50NP	NP-V1	<20
	NP-V2	<20
	NP-V3	<20
	NP-V4	<20
	NP-V5	<20

Table S5. Scoring scheme to assess the health of guinea pigs post-challenge, related to figure 4

**Score (1-4)**	**Description of Animal**
**1**	Healthy
**2**	Ruffled fur and Hunched posture (Triggers 2nd observation)
**3**	A score of 2 plus 1 additional clinical sign such as, Lethargy, Orbital tightening, and/or >15% weight loss (Triggers 3rd observation)
**4**	A score of 3 plus 1 additional clinical sign such as, Refusal to stampede, or any neurologic signs (rectal prolapse, seizures, tremors, head tilt, paralysis, etc.) OR >20% weight loss - Immediate Euthanasia

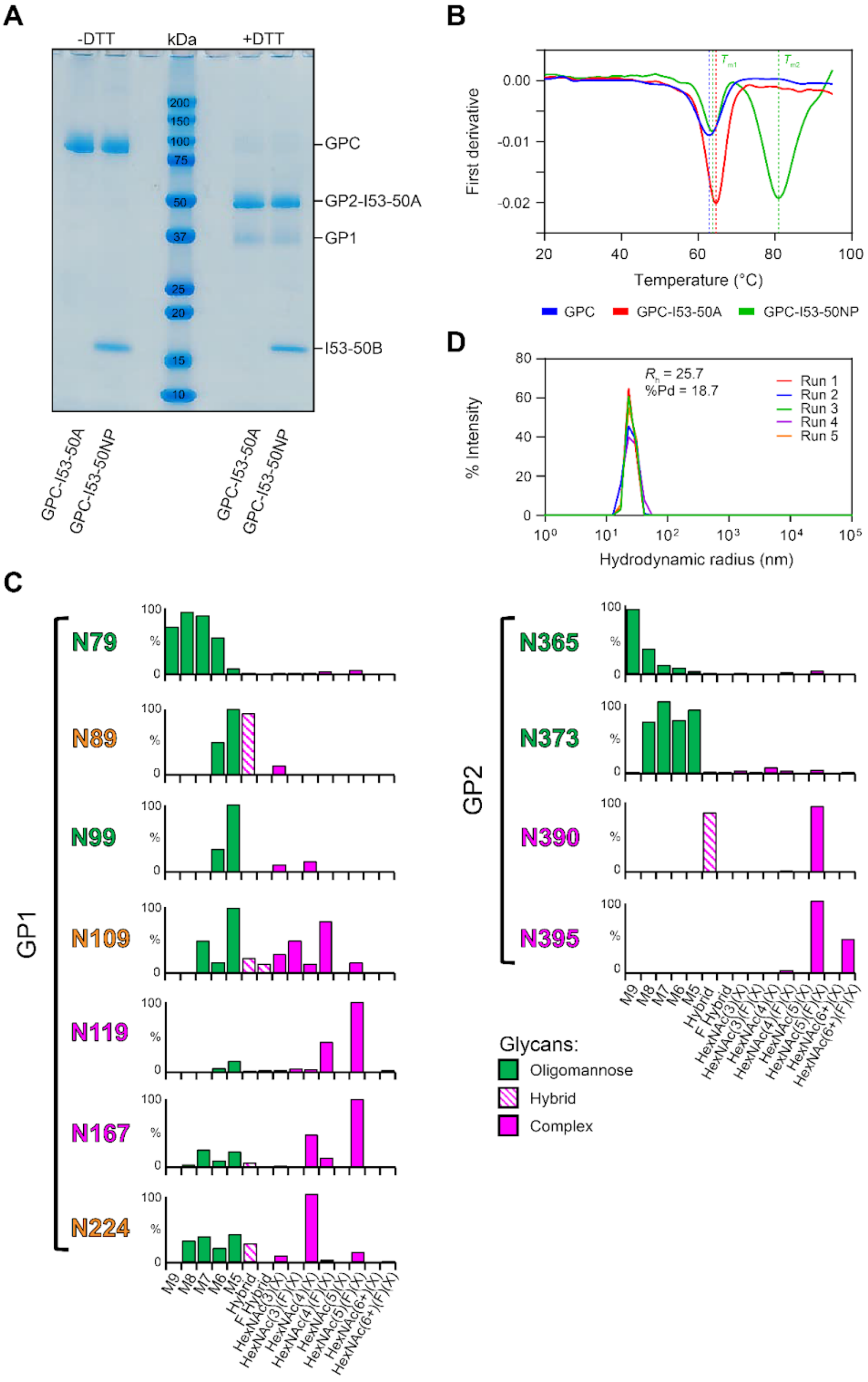
A
-DTT
kDa
+DTT
200
150
100
75
50
37
25
20
15
10
GPC
GP2-I53-50A
GP1
I53-50B
GPC-I53-50A
GPC-I53-50NP
GPC-I53-50A
GPC-I53-50NP
B
First derivative
0.00
-0.01
-0.02
T_{m1}
T_{m2}
20
40
60
80
100
Temperature (°C)
GPC
GPC-I53-50A
GPC-I53-50NP
D
% Intensity
80
60
40
20
0
R_h = 25.7
%Pd = 18.7
Run 1
Run 2
Run 3
Run 4
Run 5
10^0
10^1
10^2
10^3
10^4
10^5
Hydrodynamic radius (nm)
C
GP1
N79
N89
N99
N109
N119
N167
N224
GP2
N365
N373
N390
N395
100
%
0
M9
M8
M7
M6
M5
Hybrid
F Hybrid
HexNAc(3)(X)
HexNAc(3)(F)(X)
HexNAc(4)(X)
HexNAc(4)(F)(X)
HexNAc(5)(X)
HexNAc(5)(F)(X)
HexNAc(6+)(X)
HexNAc(6+)(F)(X)
Glycans:
Oligomannose
Hybrid
Complex

Figure S1. Biophysical characterization of GPC-I53-50A and GPC-I53-50NPs, related to figure 1 and 2

(A) SDS-PAGE analysis of GPC-I53-50A and GPC-I53-50NP in the absence (-DTT) and presence (+DTT) of dithiothreitol (DTT). (B) NanoDSF curves of GPC, GPC-I53-50A, and GPC-I53-50NP. The dotted lines indicate the melting tempera-tures (T_m), which is defined as the temperature where 50% of the protein is unfolded. GPC-I53-50NP has two melting temperatures; one for unfolding of GPC (T_{m1}) and one for the I53-50NP core (T_{m2}). Representative melting curve of at least two technical replicates is shown. (C) Site-specific glycan distribution of N-linked glycans on GPC-I53-50A as determined by LC-MS. The bar graphs represent the relative quantities of each glycan group with oligomannose-type glycan series (M9 to M5; Man9GlcNAc2 to Man5GlcNAc2) (green), a fucosylated and fucosylated hybrid glycans (Hybrid & F Hybrid) (dashed pink) and complex glycans grouped according to fucosylation and the number of antennae (HexNAc(3)(X) to HexNAc(6+)(F)(X)) (pink). (D) DLS data of GPC-I53-50NPs with the average polydispersity (%Pd) and hydrodynamic radius (R_h) of 5 runs shown. An overlay of each run is shown. A %Pd < 15 is considered a monodisperse population.

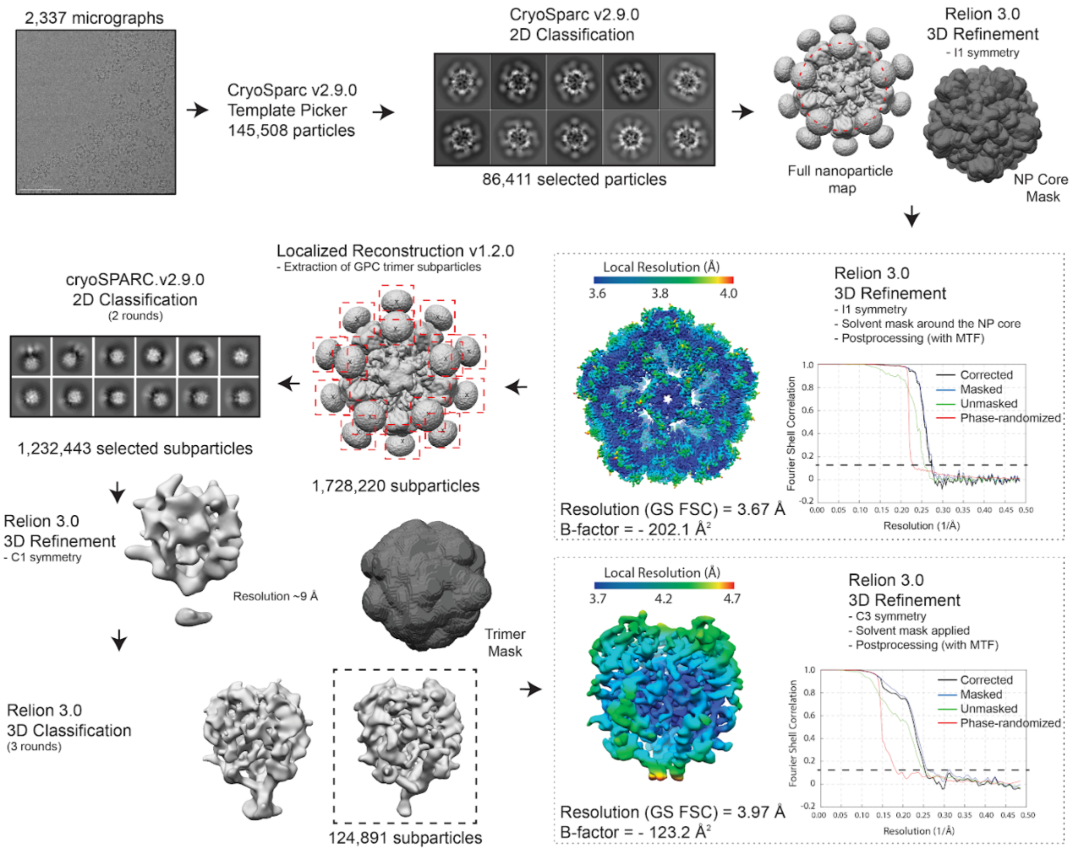

Figure S2. CryoEM data processing workflow for GPC-I53-50 nanoparticles, related to figure 3

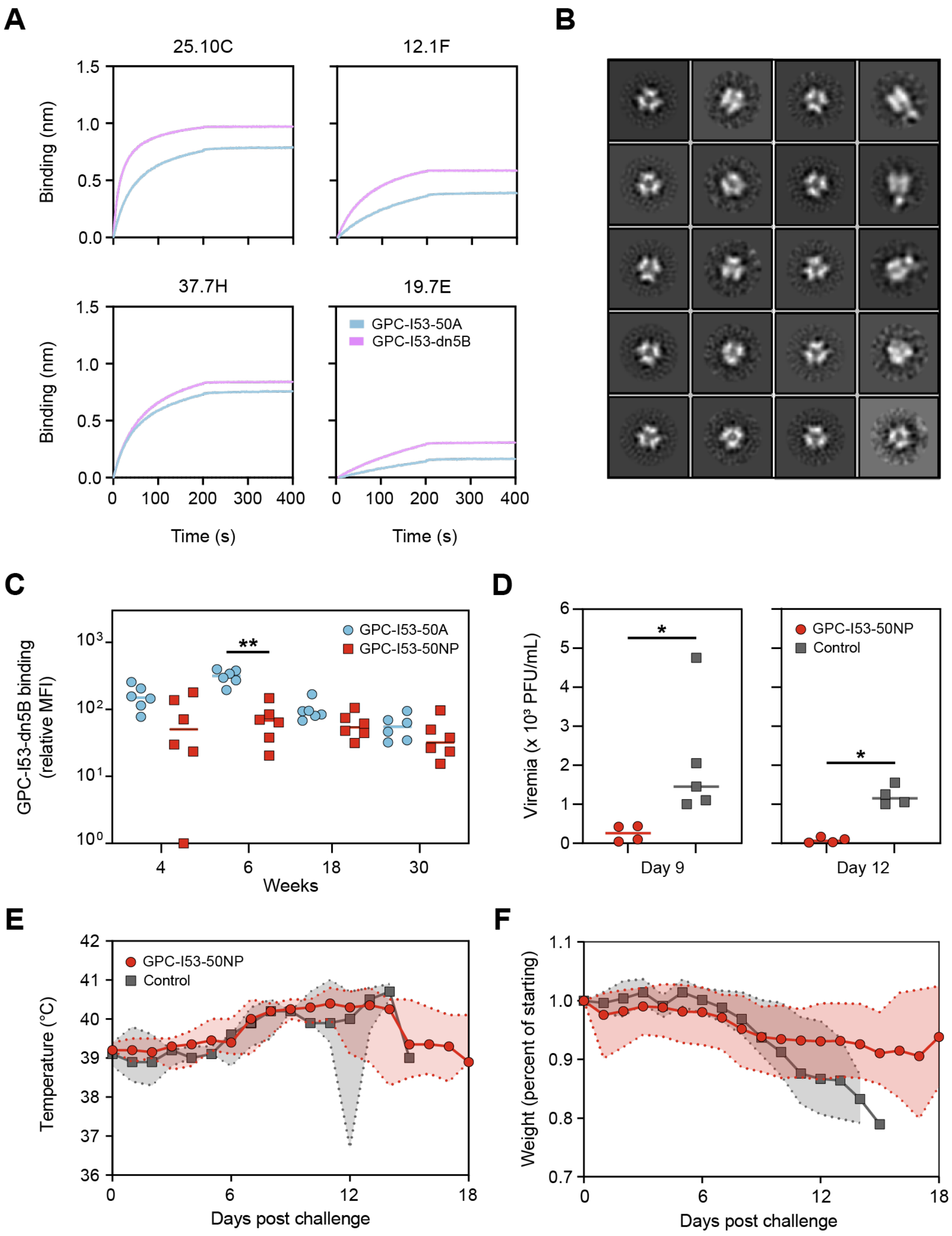

Figure S3. Antigenic and structural characterization of GPC-dn5B, IgM responses in vaccinated rabbits, and viremia, body temperature and weight alteration in vaccinated and control guinea pigs after challenge, related to figure 4

(A) Sensorgrams from BLI experiments comparing the binding to GPC-specific mAbs 25.10C, 12.1F, 37.7H, and 19.7E by GPC-I53-50A and GPC-dn5B. (B) 2D-class averages from nsEM with GPC-dn5B. (C) Relative mean fluorescence intensity (MFI) of serum IgM binding to GPC-I53-dn5B measured with a Luminex-based serology assay. Horizontal bars represent medians. Statistical differences between two groups (n = 6 rabbits) were determined using two-tailed Mann–Whitney U-tests (**p < 0.01). (D) Median RNA viral loads in vaccinated and control guinea pigs after challenge at day 9 and day 12. Statistical differences between two groups (day 9: n = 4 for vaccinated, n = 5 for controls; day 12: n = 4 for vaccinated and controls) were determined using two-tailed Mann–Whitney U-tests (*p < 0.05). (E) Median body

temperature over time of vaccinated and control guinea pigs after challenge. The shaded area indicates the range. (F) Median weight alteration in vaccinated and control guinea pigs over time after challenge. The shaded area indicates the range. The same color coding was used as in panel E.

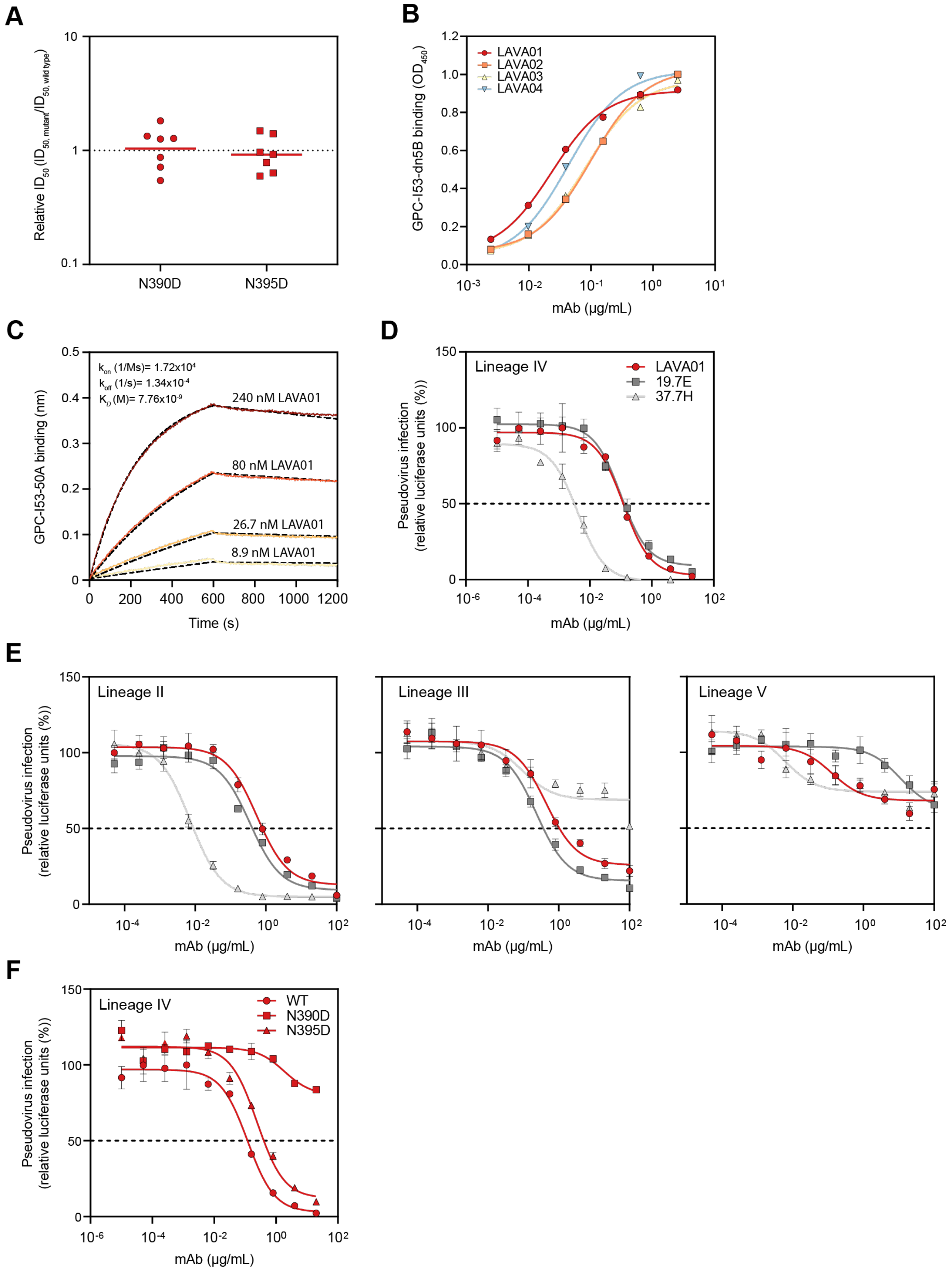

A
Relative ID50 (ID50, mutant/ID50, wild type)
N390D
N395D
B
GPC-I53-dn5B binding (OD450)
LAVA01
LAVA02
LAVA03
LAVA04
mAb (µg/mL)
C
GPC-I53-50A binding (nm)
kon (1/Ms)= 1.72x10⁴
koff (1/s)= 1.34x10⁻⁴
KD (M)= 7.76x10⁻⁹
240 nM LAVA01
80 nM LAVA01
26.7 nM LAVA01
8.9 nM LAVA01
Time (s)
D
Pseudovirus infection (relative luciferase units (%))
Lineage IV
LAVA01
19.7E
37.7H
mAb (µg/mL)
E
Lineage II
Lineage III
Lineage V
mAb (µg/mL)
F
Lineage IV
WT
N390D
N395D
mAb (µg/mL)

Figure S4. Dependency of rabbit serum and LAVA01 neutralization on N390 and N395 glycans, binding of isolated mAbs to GPC, kinetics of LAVA01 binding, and comparison of LASV pseudovirus neutralization between LAVA01, 19.7E and 37.7H, related to figure 5

(A) Relative ID_{50} for the N390D and N395D pseudovirus mutant relative to the parental lineage IV (Josiah) pseudovirus for all rabbit sera that showed an autologous neutralization ID_{50} titer >20. The dotted line indicates a RID_{50} of 1, i.e. the mutation has no effect on neutralization. (B) Binding of LAVA01-LAVA04 to GPC-dn5B as determined by ELISA. (C) Representative sensorgams from a BLI experiment to determine the binding kinetics of LAVA01 to GPC-I53-50A. Fit curves used for calculating kinetics values shown as black lines. (D) Neutralization of lineage IV (Josiah) pseudovirus by LAVA01, 19.7E, and 37.7H, as indicated in the legend. (E) Neutralization of lineage II (NIG08-A41; left), III (CSF; middle), and V (Bamba; right) pseudovirus by LAVA01, 19.7E, and 37.7H. The mean and SEM of at least two technical replicates are shown. (F) Neutralization curves of N390D or N395D pseudovirus mutants compared to the parental lineage IV pseudovirus (WT) by LAVA01. (D)-(F) The dotted line indicates 50% neutralization.

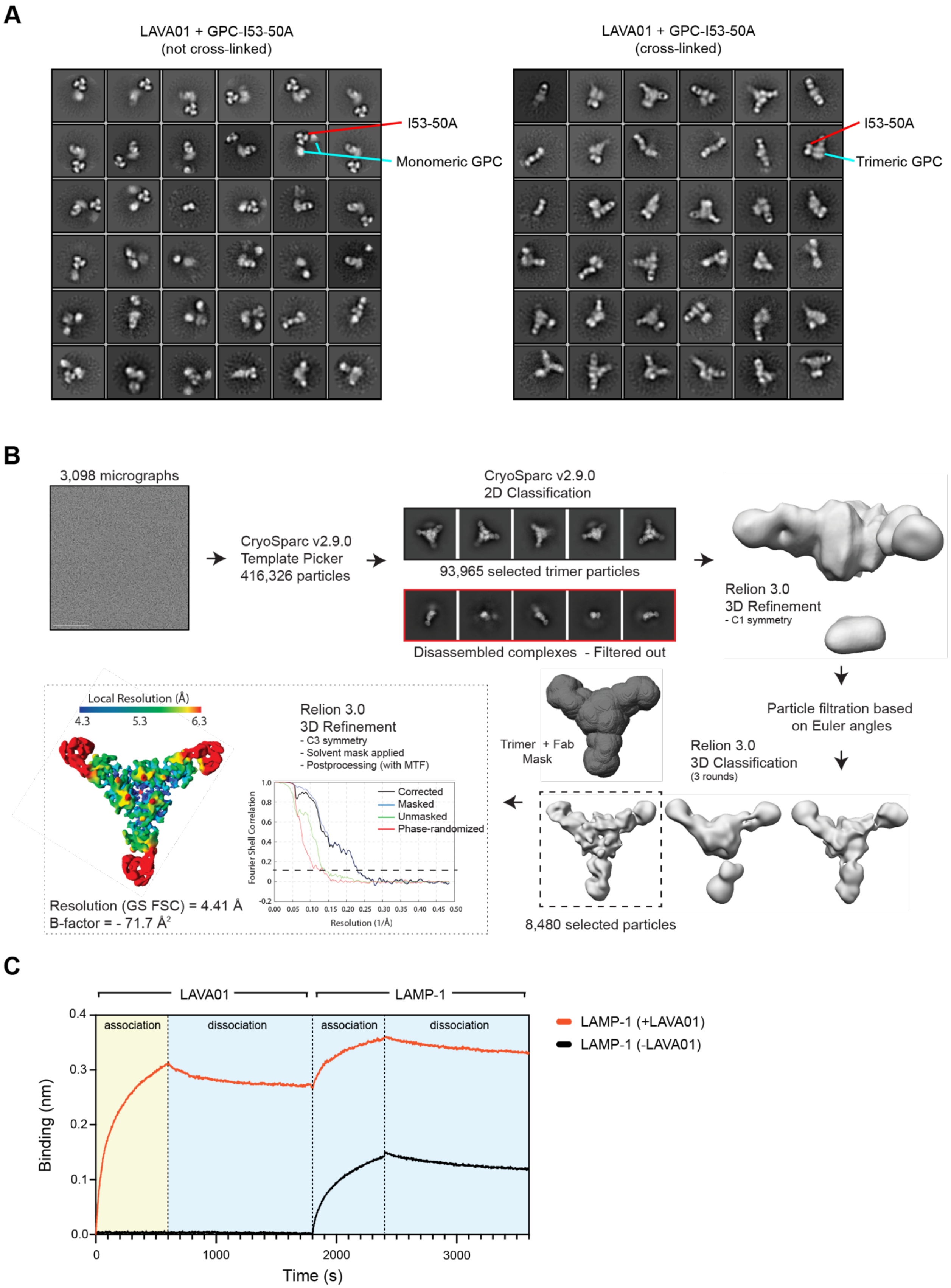

A
LAVA01 + GPC-I53-50A
(not cross-linked)
I53-50A
Monomeric GPC
LAVA01 + GPC-I53-50A
(cross-linked)
I53-50A
Trimeric GPC
B
3,098 micrographs
CryoSparc v2.9.0
Template Picker
416,326 particles
CryoSparc v2.9.0
2D Classification
93,965 selected trimer particles
Disassembled complexes - Filtered out
Relion 3.0
3D Refinement
- C1 symmetry
Particle filtration based
on Euler angles
Relion 3.0
3D Classification
(3 rounds)
Trimer + Fab
Mask
8,480 selected particles
Local Resolution (Å)
4.3
5.3
6.3
Relion 3.0
3D Refinement
- C3 symmetry
- Solvent mask applied
- Postprocessing (with MTF)
Corrected
Masked
Unmasked
Phase-randomized
Fourier Shell Correlation
Resolution (1/Å)
Resolution (GS FSC) = 4.41 Å
B-factor = - 71.7 Å²
C
LAVA01
LAMP-1
association
dissociation
association
dissociation
Binding (nm)
Time (s)
LAMP-1 (+LAVA01)
LAMP-1 (-LAVA01)

Figure S5. CryoEM data processing workflow for complexes of chemically cross-linked GPC-I53-50A with LAVA01, related to figure 5

(A) 2D-class averages from nsEM with complexes of LAVA01 with GPC-I53-50A (left) and chemically cross-linked GPC-I53-50A (right). The I53-50A component and GPC are indicated. (B) CryoEM data processing workflow for complexes of LAVA01 with chemically cross-linked GPC-I53-50A. (C) Representative sensorgram from BLI experiments showing the binding of recombinant LAMP-1 ectodomain to GPC-I53-50A, complexed (+LAVA01) or uncomplexed (-LAVA01) with LAVA01. Loading of the sensor with GPC-I53-50A is not shown. The yellow background represents binding conditions at pH 8.0. The light blue background represents binding conditions at pH 5.0.
