# Supplementary material for: Lassa virus glycoprotein nanoparticles elicit neutralizing antibody responses and protection
Source: Cell Host Microbe. 2022 Dec 14;30(12):1759–1772.e12. doi: 10.1016/j.chom.2022.10.018 (PMC9794196; doi:10.1016/j.chom.2022.10.018)
Supplement: Document S2. Article plus supplemental information [file mmc2.pdf]

Cell Host & Microbe

Article

Lassa virus glycoprotein nanoparticles elicit neutralizing antibody responses and protection

Graphical abstract

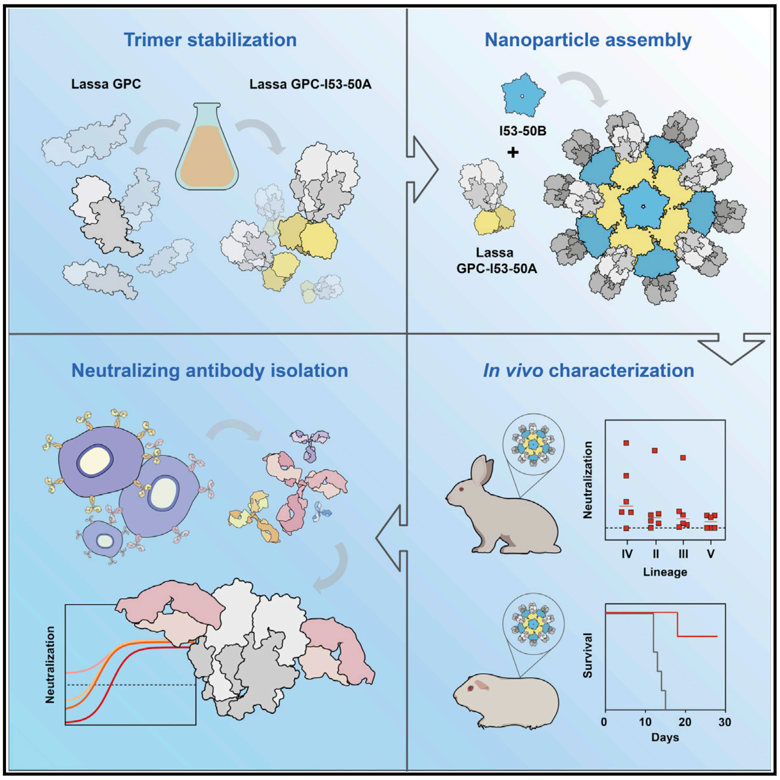

Authors

Philip J.M. Brouwer,
Aleksandar Antanasijevic,
Adam J. Ronk, ..., Alexander Bukreyev,
Andrew B. Ward, Rogier W. Sanders

Correspondence

andrew@scripps.edu (A.B.W.),
r.w.sanders@amsterdamumc.nl (R.W.S.)

In brief

There is no vaccine against Lassa fever. Brouwer et al. generate stabilized Lassa virus glycoproteins presented on two-component protein nanoparticles. *In vivo* characterization of these nanoparticle vaccines revealed that they induce potent antibody responses in rabbits, one targeting a previously undefined epitope, and protect guinea pigs from the Lassa virus challenge.

Highlights

- Lassa virus glycoprotein complex (GPC) trimer stabilization by fusion to a trimeric protein nanoparticle component
- Efficient assembly of two-component nanoparticles presenting twenty GPC trimers
- GPC nanoparticles induce antibody responses in rabbits and protect guinea pigs from Lassa fever
- Isolated NAb from GPC nanoparticle-immunized rabbit defines site of vulnerability on GPC

Brouwer et al., 2022, Cell Host & Microbe *30*, 1759–1772
December 14, 2022 © 2022 The Author(s). Published by Elsevier Inc.
https://doi.org/10.1016/j.chom.2022.10.018

CellPress

Cell Host & Microbe

CellPress OPEN ACCESS

Article

Lassa virus glycoprotein nanoparticles elicit neutralizing antibody responses and protection

Philip J.M. Brouwer,[1,2,9] Aleksandar Antanasijevic,[2,3,9] Adam J. Ronk,[4,5] Helena Müller-Kräuter,[6] Yasunori Watanabe,[7] Mathieu Claireaux,[1] Hailee R. Perrett,[2] Tom P.L. Bijl,[1] Marloes Grobben,[1] Jeffrey C. Umotoy,[1] Angela I. Schriek,[1] Judith A. Burger,[1] Khadija Tejjani,[1] Nicole M. Lloyd,[4,5] Thijs H. Steijaert,[1] Marlies M. van Haaren,[1] Kwinten Sliepen,[1] Steven W. de Taeye,[1] Marit J. van Gils,[1] Max Crispin,[7] Thomas Strecker,[6] Alexander Bukreyev,[4,5] Andrew B. Ward,[2,3,*] and Rogier W. Sanders[1,8,10,*]

[1]Department of Medical Microbiology and Infection Prevention, Amsterdam University Medical Centers, Location AMC, University of Amsterdam, Amsterdam Infection & Immunity Institute, 1105 AZ Amsterdam, the Netherlands

[2]Department of Integrative, Structural and Computational Biology, The Scripps Research Institute, La Jolla, CA 92037, USA

[3]International AIDS Vaccine Initiative Neutralizing Antibody Center, The Scripps Research Institute, La Jolla, CA 92037, USA

[4]Department of Pathology, University of Texas Medical Branch, Galveston, TX 77550, USA

[5]Galveston National Laboratory, University of Texas Medical Branch, Galveston, TX 77550, USA

[6]Institute of Virology, Philipps University Marburg, 35043 Marburg, Germany

[7]School of Biological Sciences, University of Southampton, Southampton SO17 1BJ, UK

[8]Department of Microbiology and Immunology, Weill Medical College of Cornell University, New York, NY 10021, USA

[9]These authors contributed equally

[10]Lead contact

*Correspondence: andrew@scripps.edu (A.B.W.), r.w.sanders@amsterdamumc.nl (R.W.S.)

https://doi.org/10.1016/j.chom.2022.10.018

SUMMARY

The Lassa virus is endemic in parts of West Africa, and it causes hemorrhagic fever with high mortality. The development of a recombinant protein vaccine has been hampered by the instability of soluble Lassa virus glycoprotein complex (GPC) trimers, which disassemble into monomeric subunits after expression. Here, we use two-component protein nanoparticles consisting of trimeric and pentameric subunits to stabilize GPC in a trimeric conformation. These GPC nanoparticles present twenty prefusion GPC trimers on the surface of an icosahedral particle. Cryo-EM studies of GPC nanoparticles demonstrated a well-ordered structure and yielded a high-resolution structure of an unliganded GPC. These nanoparticles induced potent humoral immune responses in rabbits and protective immunity against the lethal Lassa virus challenge in guinea pigs. Additionally, we isolated a neutralizing antibody that mapped to the putative receptor-binding site, revealing a previously undefined site of vulnerability. Collectively, these findings offer potential approaches to vaccine and therapeutic design for the Lassa virus.

INTRODUCTION

Lassa virus (LASV) is an old world mammarenavirus and the causative agent of Lassa fever, a viral hemorrhagic fever that is endemic in parts of West Africa. The virus is shed in the urine and feces of its natural reservoir, the multimammate rat (*Mastomys natalensis*), and spreads to humans by contact or ingestion of the rat's excrement or saliva.[1] While the majority of infections are zoonotic, human-to-human transmissions, primarily in the form of nosocomial transmissions, have also been reported.[2–4] An estimated 100,000–300,000 people are infected with LASV each year, with 5,000–10,000 succumbing to the disease.[5] These numbers are, however, likely an underestimation due to lack of proper diagnostics in the impoverished, mostly rural, endemic areas, and the nonspecific febrile symptoms of Lassa fever. Although the majority of infections are benign, for hospitalized patients, the case-fatality rate is around 25%.[6] Recently, Nigeria has experienced several serious LASV outbreaks, with around 30% of diagnosed patients not surviving the infection.[6,7] Seven distinct phylogenetic lineages of LASV have been identified, which cluster based on their geographic location. Lineages II and III are the most common lineages in Nigeria, whereas lineage IV is the most dominant lineage in Sierra Leone and Guinea.[8,9] A vaccine against LASV would ideally be able to confer protection against all currently known lineages.

Recent animal studies demonstrated that administration of neutralizing antibodies (NAbs) isolated from humans previously infected with LASV conferred 100% protection against a lethal LASV challenge, providing impetus for the development of a vaccine that induces potent NAb responses.[10–12] The glycoprotein complex (GPC) is expressed as a trimer on the viral surface and constitutes the sole target for NAbs. Each GPC protomer consists of two non-covalently bound subunits: the membrane-anchored GP2, which contains the fusion peptide, and

This is an open access article under the CC BY license (http://creativecommons.org/licenses/by/4.0/).

GP1, which possesses receptor-binding sites for both alpha-dystroglycan and lysosome-associated membrane protein-1 (LAMP-1), the two receptors involved in viral entry and lysosomal escape, respectively.[13–15] Following the observations that stabilized prefusion glycoprotein trimers of SARS-CoV-2, HIV-1, and RSV induced strong NAb responses,[16–18] recombinantly expressed GPC that stably maintains a trimeric prefusion state may represent a promising immunogen to induce potent humoral immune responses against LASV. Indeed, the majority of known NAbs target prefusion GPC, of which the most potent ones target an epitope that spans multiple protomers.[19,20] Although the development of a prefusion-stabilized GPC constituted an important first step for recombinant protein vaccine design, several challenges remain.[14] First of all, prefusion GPC trimers rapidly dissociate into monomers upon expression, resulting in the loss of NAb epitopes and exposure of immunodominant non-NAb epitopes on the non-glycosylated GPC interior.[21] Second, GPC is covered by a dense glycan shield making it a poorly immunogenic antigen.[22,23] Finally, GPC is sequence diverse, necessitating the induction of broadly NAb (bNAb) responses to neutralize the several lineages of LASV, which further complicates the development of an effective pan-LASV vaccine.[8]

Presenting prefusion-stabilized glycoproteins on computationally designed two-component protein nanoparticles (NPs) has greatly enhanced vaccine-induced antibody responses to RSV, influenza, and HIV-1.[24–26] The most widely used two-component NP design is I53-50, which is made up of twenty trimeric (I53-50A or variants thereof) and twelve pentameric (I53-50B) subunits that self-assemble *in vitro* to form monodisperse icosahedral particles with a diameter of approximately 30 nm.[27] Computational redesign of the trimeric subunit has generated an exterior-facing N-terminal helical extension on I53-50A that allows facile genetic fusion of trimeric antigens. This has enabled the generation of I53-50 NP (I53-50NP) vaccines featuring RSV-F, HIV-1 Env, and SARS-CoV-2 S glycoproteins.[24,25,28] Here, we describe the design and production of I53-50NPs that present native-like GPC trimer antigens of LASV. We applied electron microscopy (EM), biolayer interferometry (BLI), and differential scanning fluorimetry (nanoDSF) to confirm the appropriate structural and antigenic properties of the designed antigens. Finally, we assessed the immunogenicity of native-like GPC as free trimers and in the context of the NP. The GPC NPs consistently induced strong antibody responses in immunized rabbits. Furthermore, vaccination with these NPs protected guinea pigs from LASV-induced mortality. Finally, through analysis of memory B cells from immunized rabbits, we identified a glycan-dependent NAb targeting the putative LAMP-1 binding site on GPC.

RESULTS

Fusion to I53-50A improves LASV GPC trimerization

Previous work with I53-50NPs presenting HIV-1 Env and RSV-F suggested that fusion to I53-50A can have a stabilizing effect on the displayed glycoprotein.[24,29] Therefore, in an attempt to improve trimerization, we genetically fused the previously described prefusion-stabilized GPCysR4 (Josiah strain, lineage IV[14]) to I53-50A (Figure 1A). The resulting construct, designated GPC-I53-50A for simplicity, was expressed in HEK293 cells and purified by streptactin-based affinity purification followed by size-exclusion chromatography (SEC) to yield a reasonable amount (~0.7 mg/L of cells) of efficiently cleaved fusion proteins (Figure S1A). Non-scaffolded GPC was expressed poorly (<0.1 mg/L of cells). Whereas blue-native polyacrylamide gel electrophoresis (BN-PAGE) showed no clear trimer band for non-scaffolded GPC protein, GPC-I53-50A was exclusively trimeric (Figure 1B). 2D class-averages from negative-stain EM (nsEM) analyses revealed that the large majority of non-scaffolded GPC proteins were monomeric, while the GPC-I53-50A fusion protein was predominantly trimeric with the GPC and I53-50A components readily discernible (Figure 1C). Fusion to I53-50A increased the thermostability of GPC from 62.7°C to 64.5°C (Figure S1B).

To probe the antigenicity of GPC-I53-50A, we performed BLI-based binding experiments. A panel of NAbs was immobilized and binding to an equimolar amount of I53-50A-scaffolded, and non-scaffolded GPC was assessed (Figure 1D). We observed markedly improved binding to GP1-specific bNAbs 12.1F and 19.7E when GPC was fused to I53-50A. This was also the case for the potent and quaternary-dependent NAb 25.10C. Association of the NAb 37.7H, which targets an epitope that spans two protomers but also binds to monomeric GPC, was slightly lower for GPC-I53-50A than non-scaffolded GPC. However, no dissociation was observed for GPC-I53-50A, which is consistent with improved trimerization for this construct.

LASV GPC-I53-50A presents native-like glycans

The GPC from LASV strain Josiah used to generate GPC-I53-50A encodes 11 potential N-linked glycosylation sites (PNGSs).[30] The host-derived glycans hinder antibody recognition of GPC rendering them immunologically challenging targets.[22,23] Previous analysis of the glycan shield of GPC in the context of a native-like virus-like particle (VLP) system revealed the presence of a dense cluster of oligomannose-type glycans spanning the GP1 and GP2 subunits.[23] Since glycan modifications are sensitive reporters of protein architecture, we sought to determine the glycosylation profile of GPC-I53-50A. Glycan analysis using liquid chromatography-mass spectrometry (LC-MS) revealed a remarkable conservation in the glycan compositions between the previously described GPC on VLPs and GPC-I53-50A. Specifically, the oligomannose cluster consisting of the glycans at N79, N89, N99, N365, and N373 was conserved between the two GPC forms (Figures 1E and S1C). This underprocessing of glycans that arises due to the structural clustering of the glycans likely reflects the native-like protein architecture of GPC-I53-50A. In addition, with the exception of the glycan at N224, the remaining glycans sites were predominantly of the complex-type in both GPC-VLPs and GPC-I53-50A.

LASV GPC-I53-50A and I53-50B efficiently assemble into icosahedral nanoparticles

To assemble icosahedral I53-50NPs presenting twenty GPC trimers, SEC-purified GPC-I53-50A was mixed with I53-50B at an equimolar ratio of monomeric subunits and incubated overnight at 4°C. The assembled NPs were then separated from unassembled components by an additional SEC step. Sodium dodecyl sulfate PAGE (SDS-PAGE) of the collected higher molecular weight complexes revealed that all the expected NP components were present (Figure S1A). Only minimal amounts

CellPress
OPEN ACCESS

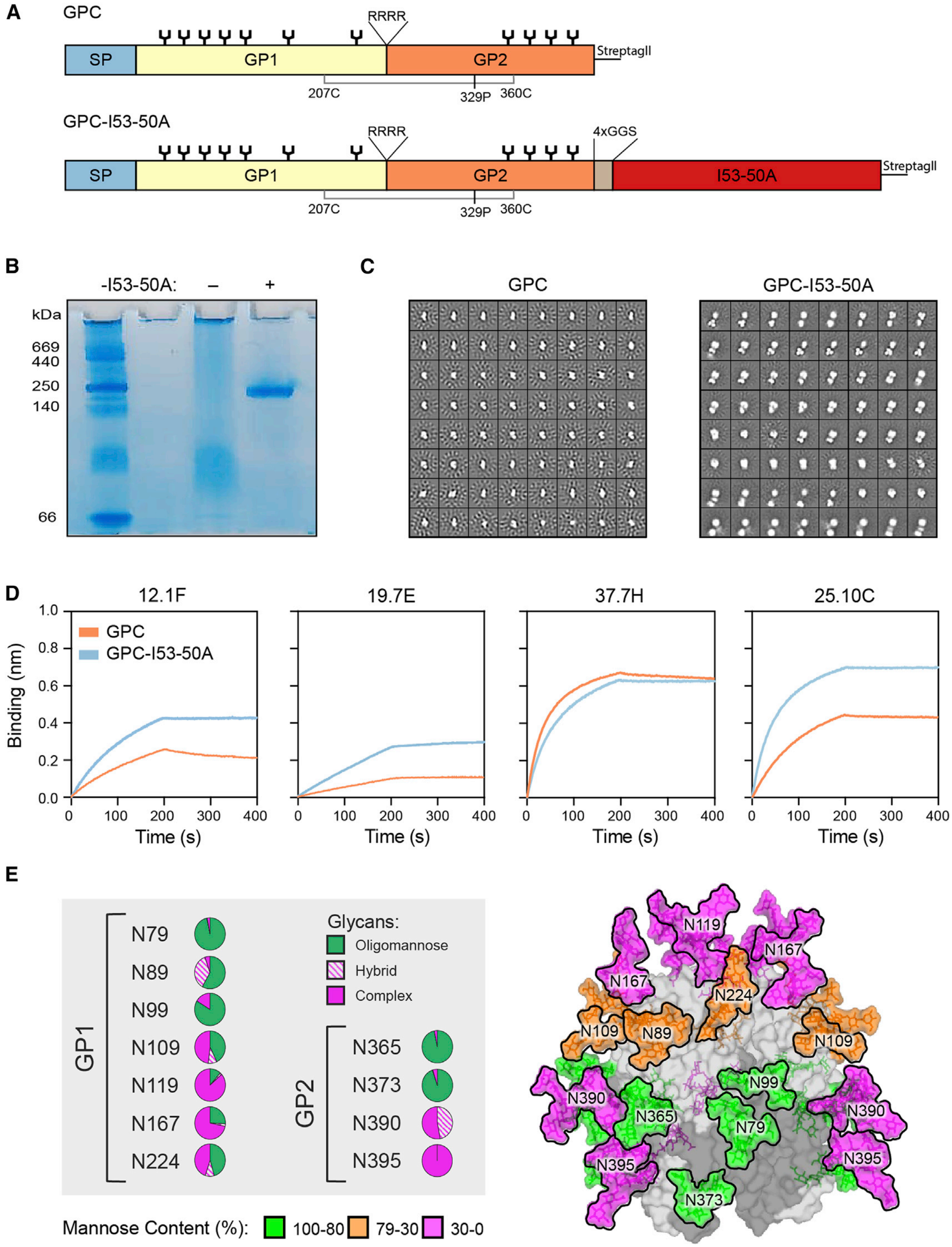

Figure 1. Biophysical characterization, antigenicity, and glycosylation of GPC-I53-50A

(A) Linear schematic of the GPC and GPC-I53-50A constructs with GPCysR4 mutations annotated. The disulfide bond (207C–360C) that connects GP1 to GP2 is shown in gray. SP, signal peptide.

(B) BN-PAGE analysis of GPC (–) and GPC-I53-50A (+).

(C) 2D-class averages from nsEM with GPC (left) and GPC-I53-50A (right).

(D) Sensorgrams from BLI experiments showing binding of GPC and GPC-I53-50A to immobilized human (b)NAbs 12.1F, 19.7E, 37.7H, and 25.10C.

(E) Site-specific glycan analysis of GPC-I53-50A. Each pie chart summarizes the quantification of oligomannose (green), hybrid (dashed pink), and complex glycans (pink) for each glycan site on GP1 or GP2. The experimentally observed glycans are modeled on the trimeric GPC structure (PDB: 5VK2[14]). The glycans are colored according to the oligomannose content as defined in the legend. GP1 and GP2 subunits are colored light gray and dark gray, respectively.

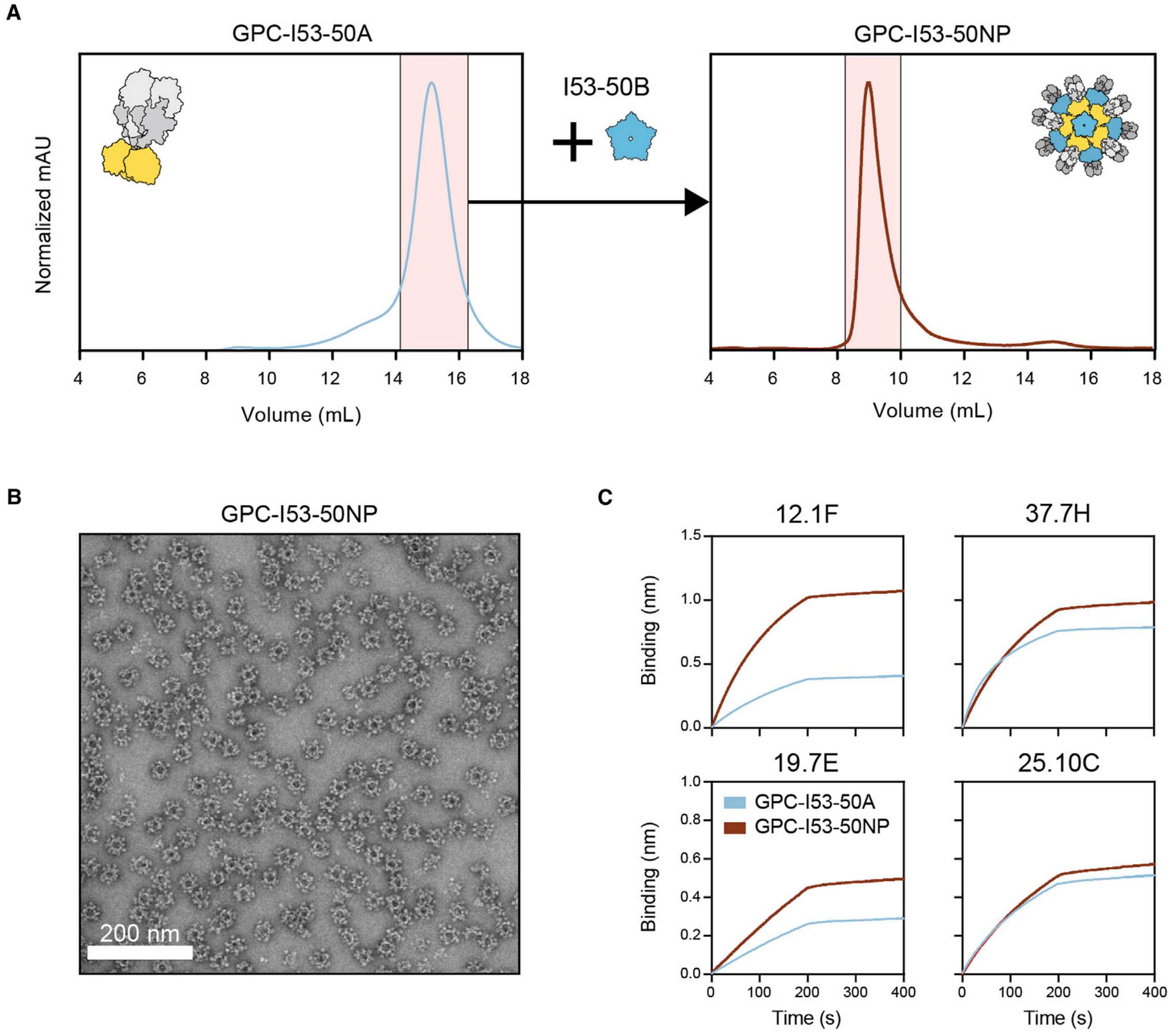

Figure 2. Biophysical and antigenic characterization of GPC-I53-50NPs

(A) Representative size-exclusion chromatograph of GPC-I53-50A (left panel) and GPC-I53-50NPs after assembly with I53-50B (right panel). Collected fractions for particle assembly or of purified GPC-I53-50NPs are shown in pink shading.

(B) Raw nsEM image of the SEC-purified GPC-I53-50NPs. White scale bar corresponds to 200 nm.

(C) Sensorgrams from BLI experiments with GPC-I53-50A and GPC-I53-50NP showing the binding of bNAbs 12.1F, 37.7H, 19.7E, and 25.10C.

of unassembled components were observed in SEC, suggesting efficient assembly of GPC-I53-50NPs (Figure 2A). nsEM experiments with the pooled fractions containing high molecular weight proteins revealed a homogeneous preparation of well-ordered icosahedral particles, with a small percentage of aggregated GPC-I53-50NPs. The latter was confirmed by dynamic light scattering (DLS), which showed a monomodal but slightly polydisperse population of particles (Figures 2B and S1D). Similar to our observations with I53-50NPs presenting native-like HIV-1 Env trimers,[25,29] NanoDSF showed a two-phased melting curve for NPs, with a melting temperature for GPC at 63.6°C and for the I53-50 components at 81°C (Figure S1B). The GPC-I53-50NPs retained the capacity to bind to the monoclonal bNAbs 19.7E, 12.1F, 37.7H, and 25.10C confirming that NP assembly did not compromise the antigenicity of GPC (Figure 2C).

High-resolution structure of unliganded LASV GPC reveals subtle structural differences to liganded LASV GPC

Next, the assembled GPC-I53-50NPs were subjected to structural characterization using cryo-EM (see Table S1 and Figure S2 for details on data collection and processing). Due to the flexible linkage between the GPC and I53-50A, we were unable to reconstruct a single high-resolution model of the entire NP antigen (Figure 3A; note the lack of structural features on the GPC

CellPress
OPEN ACCESS

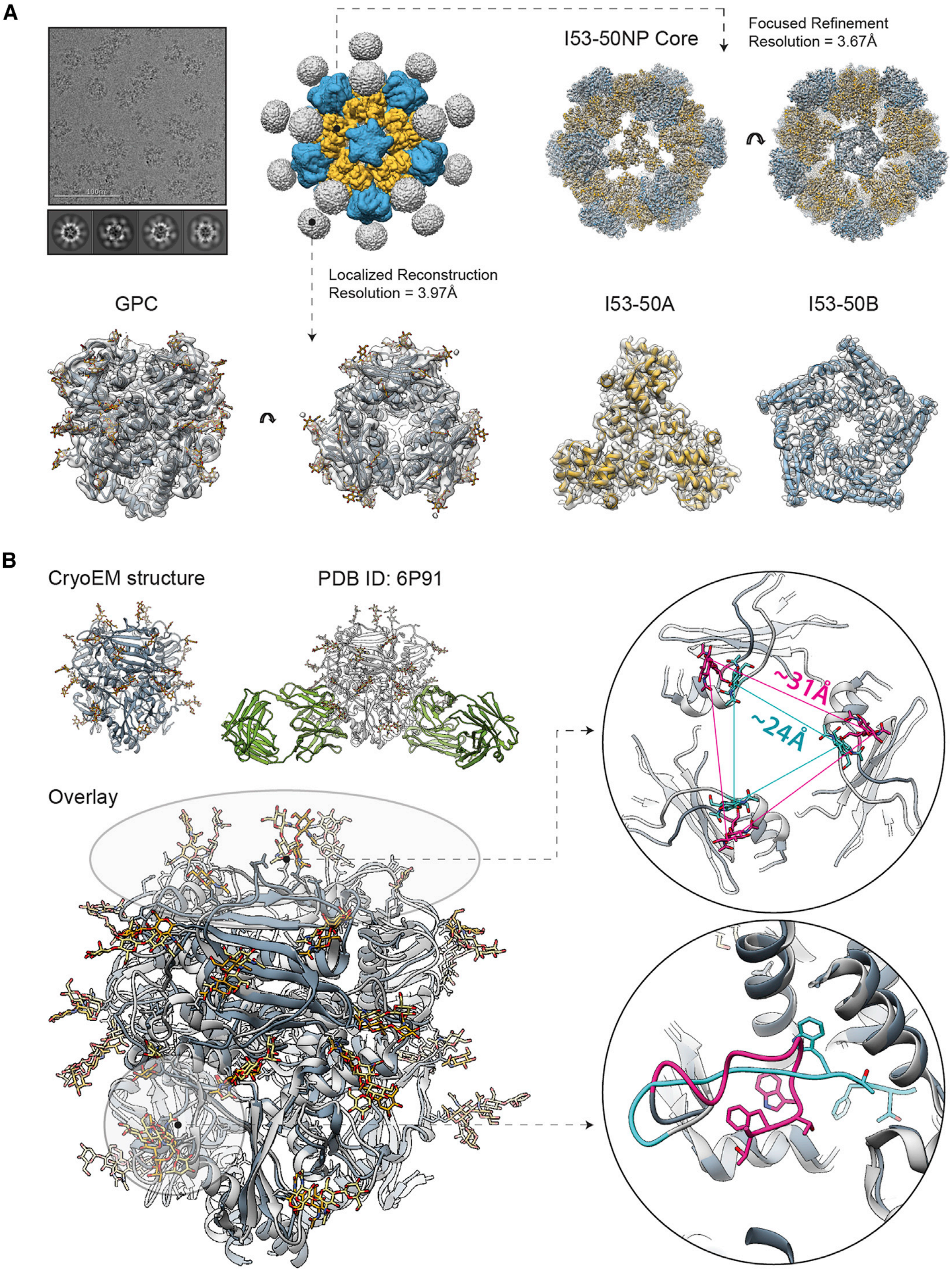

Figure 3. High-resolution cryo-EM structure of GPC-I53-50NPs

(A) Sample micrograph (white scale bar corresponds to 100 nm), 2D class averages, and initial 3D reconstruction of the GPC-I53-50NP are displayed in the top left part of the panel. Focused refinement was applied to reconstruct the 3D map of the nanoparticle core (right). The structure of the I53-50NP is shown inside the map (I53-50A—yellow; I53-50B—blue; map—transparent white surface). Localized reconstruction approach was used for analysis of the presented antigen (bottom left). Refined GPC model is shown in dark gray with glycans displayed in golden yellow.

(B) Comparison of the cryo-EM structure of GPC (dark gray) and the crystal structure of GPC (light gray) in complex with 18.5C antibody (green) (PDB: 6P91[20]) with the overlay of the two structures shown below. Comparison of the fusion peptide conformations is displayed in the bottom right part of this panel (cryo-EM model—pink; crystal structure—turquoise). Side chains of residues G260-W264 are displayed in each case. Comparison of the apex conformations in two structures is displayed in the top right part of this panel. The distances between N119 glycans are shown for each model (cryo-EM model—pink; crystal structure—turquoise).

antigens). Therefore, we separately processed the signal originating from two flexibly linked entities, the GPC trimers and I53-50NP, resulting in 3D maps at 3.97 and 3.67 Å resolution, respectively (Figure 3A). Structural models were relaxed into the reconstructed maps, and model to map fits are shown (Figure 3A, bottom row). The model refinement statistics are presented in Table S1.

The I53-50NP structure is in excellent agreement with the Rosetta-designed model and the published structure obtained with I53-50NP presenting HIV-1 BG505 SOSIP trimers (PDB: 6P6F[29]). The RMSD values for Cα atoms were 0.74 Å for the comparison with the Rosetta model and 0.51 Å for the comparison with HIV-1 SOSIP-I53-50NPs. These findings confirm that GPC fusion did not interfere with the folding of the I53-50A component or the assembly of the NP.

The reconstructed GPC model features a trimer in the prefusion state (Figure 3A). The structure revealed an overall globular protein assembly, uniformly covered in N-linked glycans. In the cryo-EM map, we observed densities corresponding to all 11 N-linked glycans for each monomer. We compared this structure to the previously reported structures obtained by X-ray crystallography and featuring stabilizing antibodies bound to a quaternary epitope at the interface of two protomers, making direct contacts with the fusion peptide (Figure 3B). Due to the high degree of similarity of the three crystal structures (PDB: 6P91, 6P95, and 5VK2[14,20]), we are only showing the comparative analysis results for one of them (PDB: 6P91[20]). Overall, our unliganded cryo-EM model displayed excellent structural homology with the reported antibody-complexed structure, with a Cα RMSD value of 0.92 Å suggesting that the fusion to the I53-50A component and NP assembly did not induce major conformational changes within the GPC.

Closer inspection of the two models revealed local differences in two regions: the fusion peptide and the trimer apex (Figure 3B, bottom right). In the unliganded state (cryo-EM structure; this study), fusion peptide residues G260-T274 expand into the cavity between two protomers with the N terminus (residue G260) facing outward. In the antibody-complexed states (PDB: 6P91, 6P95, and 5VK2[14,20]), the cavity is occupied by the HCDR3 of the 18.5C, 25.6A, or 37.7H antibodies, pushing the fusion peptide toward the center of the trimer. Solvent exposure of the fusion peptide in the unliganded prefusion state is a property shared by several other class 1 glycoproteins (e.g., influenza HA and HIV Env) and may be important for the correct onset of the fusogenic conformational change in the GPC.[31] The trimer apex also exhibited differences between the different structures (Figure 3B, top right). The unliganded structure featured a more open conformation at the apex, particularly for residues between N114 and L128. The distances between the three N119 residues from the respective protomers are ~31 Å in the unliganded structure and ~24 Å in the antibody-complexed structures. This ~7 Å difference is substantial and might affect the position and/or orientation of the glycans attached to the three N119 residues thereby influencing epitope shielding by these glycans. Finally, the local resolution of the unliganded model is lower within the central cavity of the trimer and at the apex (Figure S2), suggesting a higher degree of flexibility in these regions. While the presence of stabilizing antibodies (18.5C, 25.6A, or 37.7H) and crystal packing in the antibody-complexed structure may have resulted in increased stability of this part of the protein and more compact conformation, the current structural data are insufficient to conclude whether this conformational plasticity has any functional or immunological relevance.

LASV GPC-I53-50NPs induce strong antibody responses in rabbits

To assess the immunogenicity of recombinant native-like GPC trimers and evaluate whether presentation on I53-50NPs would provide an immunological benefit, we performed an immunization study in rabbits with GPC-I53-50A and GPC-I53-50NPs. New Zealand white rabbits (n = 6 per group) received 30 μg of GPC-I53-50A or an equimolar amount assembled into GPC-I53-50NPs formulated in squalene emulsion at weeks 0, 4, 16, and 28 (Figure 4A). Bleeds were taken after the prime (week 4), and each boost (weeks 6, 18, and 30) to determine serum GPC-specific binding and pseudovirus neutralization titers.

GPC-specific binding titers were analyzed by ELISA using immobilized GPC fused to the previously described dn5B scaffold to exclude serum Ab binding to I53-50A (Figure 4B). Importantly, GPC-I53-dn5B was almost exclusively trimeric as determined by nsEM and bound efficiently to 25.10C, 12.1F, 37.7H, and 19.7E (Figures S3A and S3B). After the first immunization, GPC-I53-50NPs induced significantly higher GPC binding IgG titers than its soluble counterpart, which did not induce Ab titers above cutoff in 4/6 rabbits (median ED_{50} of 1,557 versus 200, respectively; two-tailed Mann-Whitney *U*-test; $p = 0.0087$). A trend toward higher binding titers was also observed after the second immunization, but after the third and fourth immunizations, GPC-specific binding titers were very similar between GPC-I53-50A and GPC-I53-50NP. To gauge, whether the higher IgG binding titers in the GPC-I53-50NP animals were associated with enhanced class-switching, we analyzed the GPC-specific serum IgM response using a Luminex assay (Figure S3C). Indeed, we observed an overall trend toward lower IgM levels in the rabbits that received GPC NPs, with IgM levels being significantly lower after the second immunization (median mean fluorescence intensity [MFI] of 67 versus 320, respectively; two-tailed Mann-Whitney *U*-test; $p = 0.0022$), consistent with observations that multivalent antigen presentation accelerates class-switching.[32]

We used a previously described lentivirus-based pseudovirus assay to measure the induction of NAb responses.[19] No neutralization was induced after two immunizations (Table S2). However, after three immunizations, 5/6 rabbits in the NP group developed NAb titers with ID_{50} values ranging from 38 to 623. For the two best responders (rabbit 194 and 196), these titers increased to 2,749 and 413 after the final fourth immunization, respectively (Table S2). In contrast, only 2/6 rabbits in the GPC-I53-50A group developed NAb responses after three immunizations, which were boosted to titers of 2,565 and 560 after the final boost (Figure 4C; Table S2). In addition, we assessed the sera's ability to neutralize authentic LASV (Figure 4C; Table S3). In this assay, the virus neutralization titer is calculated as the geometric mean titers (GMTs) of the reciprocal value of the last serum dilution at which inhibition of the cytopathic effect on infected Vero E6 cells is detectable. Titers ranged from 11 to 38 in the rabbit sera that showed some neutralizing activity (2/6 rabbits in the GPC-I53-50A group and 3/6 in the GPC-I53-50NP

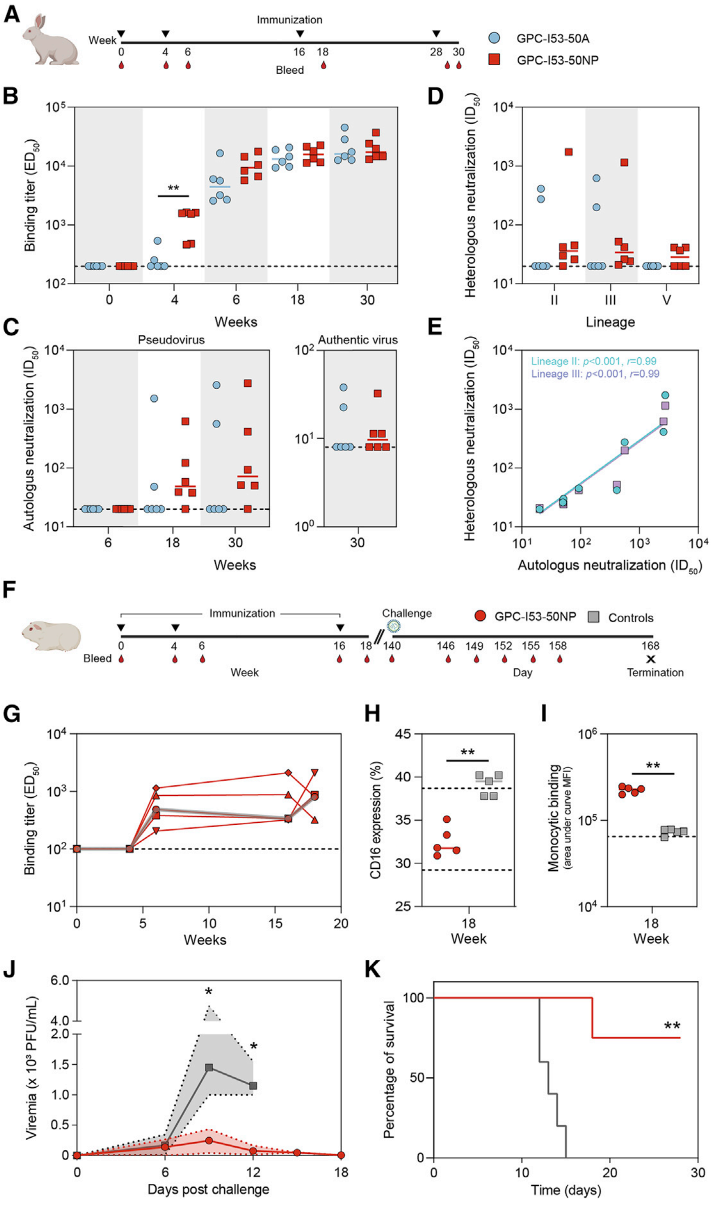

Figure 4. Immunogenicity of GPC-I53-50A and GPC-I53-50NP in rabbits and protective efficacy of GPC-I53-50NP in guinea pigs

(A) Schematic representation of the rabbit immunization schedule with color coding for each immunogen.

(B) Midpoint binding titers against GPC-I53-dn5B at weeks 0, 4, 6, 18, and 30.

(C) Midpoint NAb titers against autologous pseudovirus (lineage IV) at weeks 6, 18, and 30 (left panel). Endpoint NAb titers against authentic LASV (lineage IV) at week 30 (right panel). The dotted line indicates the lower limit of detection.

(D) Midpoint NAb titers against heterologous pseudoviruses (lineages II, III, and V) at week 30.

(E) Correlation plot of autologous NAb titers versus heterologous NAb titers (lineages II or III). The *r* and p values are shown for two-tailed Spearman correlations (n = 7 rabbits; all rabbits with an autologous neutralization $ID_{50} > 20$).

(F) Schematic representation of the guinea pig challenge study schedule.

(G) Midpoint binding titers against GPC-I53-dn5B at weeks 0, 4, 6, 16, and 18. The dark gray line represents the median.

(H) CD16 expression on NK cells after incubation with GPC-specific serum antibodies from vaccinated and control guinea pigs. The lower dotted line represents median CD16 levels after incubation with a combination of phorbol myristate acetate (PMA) and ionomycin (positive control) whereas the upper dotted line represents median CD16 levels in the absence of serum antibodies (negative control).

(I) Binding to monocytes (THP-1 cells) by GPC-specific serum antibodies from vaccinated and control guinea pigs. The lower dotted line represents median monocytes binding in the absence of serum antibodies (negative control).

(J) Median RNA viral loads in vaccinated and control guinea pigs after challenge. The shaded area indicates the range. Statistical differences between two groups (days 6 and 9: n = 4 for vaccinated, n = 5 for controls; day 12: n = 4 for vaccinated and controls) were determined using two-tailed Mann-Whitney *U*-tests (*$p < 0.05$).

(K) Kaplan-Meier curve showing survival over time for vaccinated and control guinea pigs (n = 4 for vaccinated, n = 5 for controls at day 0) following a LASV challenge. Statistical difference between the two groups was determined using log-rank tests (**$p < 0.01$). The same color coding was used as in (H). In (B)–(D), (H), and (I), the median titers are indicated by a bar. Statistical differences between groups were analyzed using the two-tailed Mann-Whitney *U*-test (*$p < 0.05$; **$p < 0.01$; n = 6 rabbits for both groups in B–D; n = 5 guinea pigs for both groups in H and I).

group). Consistent with earlier studies, it is apparent that the authentic virus is considerably more resistant to neutralization than the lentivirus-based pseudovirus assay.[19]

Considering the high degree of sequence diversity of LASV lineages over the large geographical endemic areas, the ability to elicit a broad immunological response is imperative for a successful LASV vaccine. To evaluate the breadth of the NAb response induced by our vaccine based on a LASV lineage IV strain, we generated pseudoviruses representing the heterologous LASV lineages II, III, and V and performed neutralization assays with rabbit serum from week 30 (2 weeks after the fourth vaccination) (Figure 4D; Table S2). Sera from the two rabbits that developed autologous NAb titers in the GPC-I53-50A group were also able to neutralize lineages II and III quite potently but not lineage V pseudovirus. Similarly, in the NP group, sera that neutralized lineage IV also neutralized lineages II and III to varying degrees. Serum of rabbit 194 showed broad and exceptionally high NAb titers to lineage II (ID_{50} of 1,733) and III (ID_{50} of 1,151) and low but detectable neutralizing activity to lineage V. We noted a strong correlation between NAb titers against autologous and heterologous (lineages II and III) pseudovirus, suggesting that our GPC immunogens readily elicit NAb against epitopes that are conserved among LASV lineages (Figure 4E).

LASV GPC-I53-50NP vaccination protects guinea pigs from lethal LASV challenge

To assess the protective potential of GPC-I53-50NPs, we performed a LASV challenge study. Hartley guinea pigs (n = 5) received 30 μg of GPC-I53-50NP adjuvanted in squalene emulsion at weeks 0, 4, and 16 (Figure 4F). Control animals (n = 5) received three doses of squalene emulsion. GPC-I53-50NPs induced GPC-specific Ab responses after two immunizations

(median ED_{50} of 486 at week 6), which increased after the third immunization (median ED_{50} of 803 at week 18) (Figure 4G). These GPC-specific Ab responses seem markedly lower than those observed in rabbits. In contrast to our observations in rabbits, no pseudovirus neutralization was detected after three immunizations with GPC-I53-50NPs in guinea pigs (Table S4). Together, this indicates that guinea pigs elicited an overall poorer humoral response than rabbits. Several studies have reported vaccine-induced protection in the absence of NAb responses, suggesting a role for antibody-mediated effector functions such as antibody-dependent cellular cytotoxicity (ADCC) and antibody-dependent cellular phagocytosis (ADCP).[33,34] We observed marked NK activation by serum GPC-specific antibodies from vaccinated guinea pigs as indicated by a significant decrease of CD16 expression on NK cells over unvaccinated controls (Figure 4H). Furthermore, GPC-specific antibodies displayed significant binding to monocytes, which is a proxy for antibody-induced phagocytic activity (Figure 4I), suggesting that GPC-I53-50NP induced humoral responses that are capable of both ADCC and ADCP.

At week 20, i.e., 4 weeks after the third vaccination, the animals were challenged intraperitoneally with a lethal dose (1×10^4 plaque-forming units [PFUs]) of a guinea-pig adapted LASV Josiah strain (lineage IV[35]) (Figure 4F). One guinea pig in the vaccinated group did not recover from the week 18 bleed and died pre-challenge. With a median of 145 and 170 PFU/mL, both vaccinated and unvaccinated guinea pigs, respectively, had similar viral loads 6 days after the challenge. However, at day 9, viral loads had increased dramatically in the control animals reaching a median viral load of 1,450 PFU/mL, which then decreased slightly to 1,150 PFU/mL at day 12 after challenge. In contrast, the median viral loads in the vaccinated guinea pigs were significantly lower at day 9 (258 versus 1,450 PFU/mL, two-tailed Mann-Whitney *U*-test; $p = 0.0159$) and day 12 (63 versus 1,150; two-tailed Mann-Whitney *U*-test; $p = 0.0286$) (Figures 4J and S3D). By day 15, all (5/5) control animals had met the euthanization criteria (Table S5) and were euthanized, while in the vaccinated guinea pigs viral loads had decreased further to a median of 38 PFU/mL. Nevertheless, on day 18 post-challenge, one of the vaccinated guinea pigs was moribund and had to be euthanized (Figure 4K). The remaining three animals survived to the study endpoint (day 28). All GPC-I53-50NP-vaccinated animals became febrile after challenge and all but one experienced weight loss (Figures S3E and S3F). Thus, GPC-I53-50NP vaccination did not protect the animals from infection and disease, but it did significantly protect them from mortality (log-rank test; $p = 0.0045$) (Figure 4K).

Isolation of a broadly neutralizing LASV GPC-specific mAb reveals a previously undefined site of vulnerability

Most neutralizing mAbs isolated from Lassa fever patients so far reveal GPC-B, a base-proximate epitope that includes glycans at N390 and N395, as an immunodominant site for neutralization.[19,20] Removal of these glycans drastically increased neutralization sensitivity of LASV to these NAbs, consistent with earlier reports showing that glycans are major barriers for the induction of potent NAb responses.[22,23] To assess if GPC-B-type NAbs dominated the induced NAb responses by GPC-I53-50A and GPC-I53-50NP in rabbits, we performed neutralization assays using pseudovirus lacking PNGs at either glycan N390 or N395 (Figure S4A). We observed no clear alteration in NAb titers when these glycans were removed suggesting that GPC-B-type NAbs did not dominate the neutralizing responses induced by GPC-I53-50NPs.

To characterize the NAb response in rabbit 194 in more detail, we sorted B cells and isolated mAbs. Rabbit 194 was immunized with GPC-I53-50NPs and showed the most potent and broad neutralization profile. GPC-specific IgG+ B cells were single cell sorted from peripheral blood mononuclear cells (PBMCs) by dual staining with fluorescently labeled GPC-I53-50A from the Josiah (lineage IV) and NIG08-A41 (lineage II) strain. Of the GPC-specific B cells (~15.5% of the total IgG+ B cells), ~93% was reactive to both probes, consistent with the breadth of the NAb response in this animal (Figure 5A). We cloned four mAbs, termed LAVA01-LAVA04, which bound to GPC-I53-dn5B with a midpoint binding concentration (EC_{50}) varying from 0.02 to 0.10 μg/mL (Figure S4B). LAVA01 bound with a dissociation constant of ~8 nM and was able to neutralize autologous pseudovirus with an IC_{50} of approximately 0.12 μg/mL; on par with that of 19.7E but approximately 10-fold lower than 37.7H (Figures 5B, S4C, and S4D). LAVA01 also showed neutralization potency to NIG08-A41 (lineage II) and CSF (lineage III) pseudovirus with an IC_{50} of 0.78 and 1.08 μg/mL, respectively (Figure 5C). In contrast, the prototypic bNAb 37.7H failed to reach 50% neutralization of CSF. Neither of the three mAbs, LAVA01, 37.7H, or 19.7E were able to reach 50% neutralization of the Bamba strain (lineage V) (Figure S4E). We also analyzed the potential of LAVA01 to neutralize authentic Josiah LASV. LAVA01 neutralized authentic LASV at a titer of 21 μg/mL (Figure 5D). 19.7E was less potent than LAVA01 showing a neutralization titer of 42 μg/mL, whereas 37.7H was more potent, neutralizing authentic LASV with a titer of 5 μg/mL.

To map the epitope of LAVA01, we performed neutralization assays with pseudovirus (lineage IV) that had the N390 or N395 glycan knocked out. Whereas removing the N395 glycan slightly decreased LAVA01 neutralization, removal of the N390 glycan completely abrogated it, suggesting that the N390 glycan is a key component of the LAVA01 epitope (Figure S4F).

To obtain detailed structural information of the epitope of this new NAb, we performed cryo-EM studies using GPC-I53-50A in complex with the LAVA01 Fab. Initial attempts revealed that most trimers disassembled into monomers upon complex formation, resulting in highly heterogeneous 2D-classes, which could not be used to reconstruct a 3D-map (Figure S5A). To resolve this problem, we chemically cross-linked GPC-I53-50A with glutaraldehyde prior to complexing with the LAVA01 Fab. This increased the relative amount of trimeric GPC, although monomers were still visible in the micrographs and 2D classes (Figures S5A and S5B). Data processing resulted in a 4.41 Å map of the complex, from which we derived an atomic model (Figures 5E and S5B). Due to the relatively modest resolution, amino acid side chains of the LAVA01 Fab were not built past Cβ. Inspection of the model showed that LAVA01 binds to a previously unknown neutralizing epitope comprising elements of GP1 and GP2 within a single protomer and can bind the GPC trimer at the maximum stoichiometry of 3. The main peptidic components of the epitope are β5, β6, L1, and HR1 (Figure 5F). Moreover, the antibody makes extensive contacts with the N109 glycan, through the HCDR2 and LCDR1 loops, and with the N390

CellPress
OPEN ACCESS

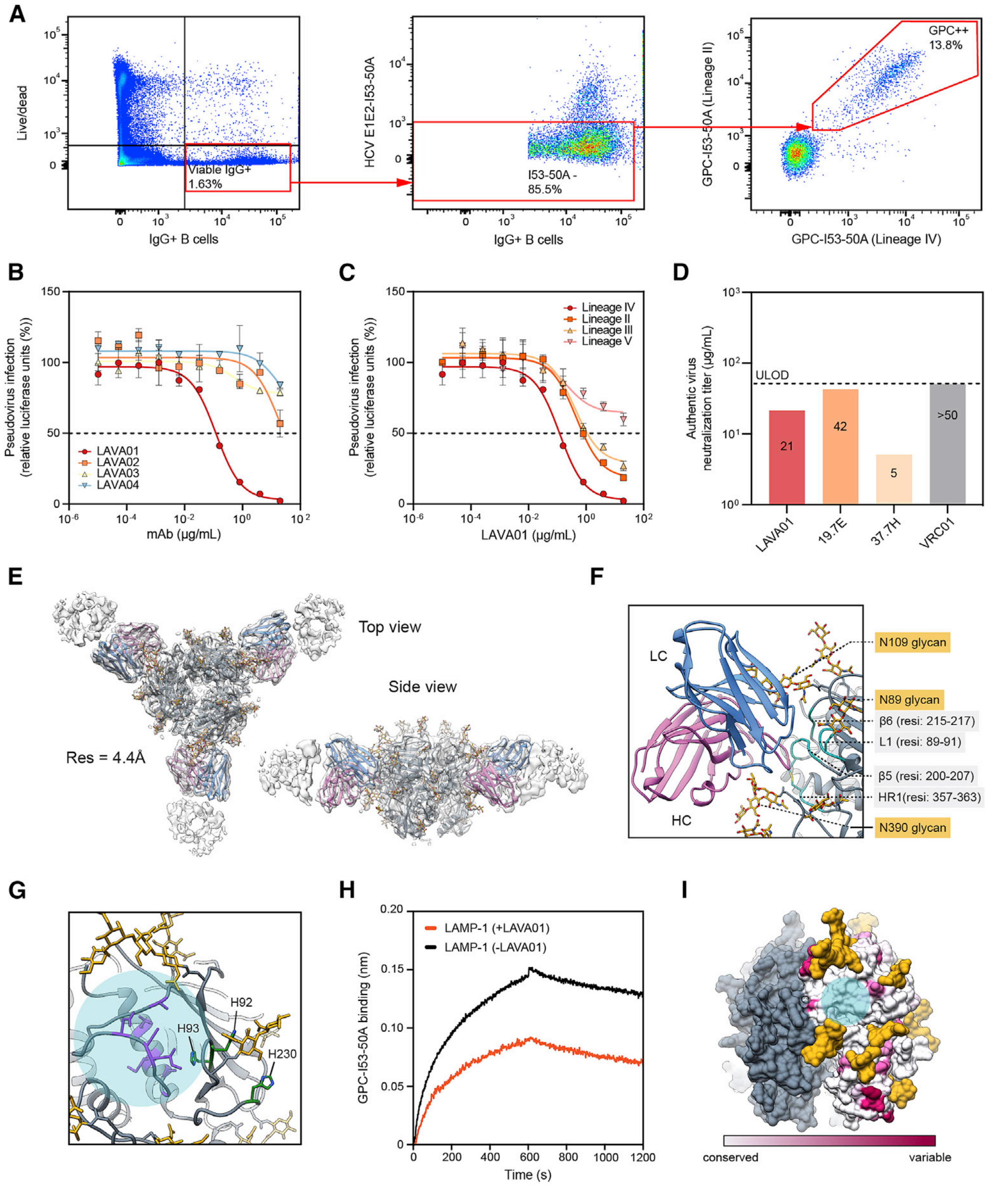

Figure 5. Isolation of a monoclonal NAb that targets a previously undefined site of vulnerability on GPC

(A) Representative gating strategy for the isolation of GPC-specific B cells. The lymphocyte population was selected, and doublets were excluded (not shown). From live IgG+ B cells (left), cells were selected that showed low reactivity to I53-50A (middle) after which the double-positive GPC-specific B cells were sorted (right).

(B) Pseudovirus neutralization of lineage IV (Josiah) by LAVA01-LAVA04. The dotted line indicates 50% neutralization. Shown are the mean and SEM of two technical replicates.

(C) Pseudovirus neutralization of lineages II, III, IV, and V by LAVA01. The dotted line indicates 50% neutralization. Shown are the mean and SEM of two technical replicates.

(D) Endpoint neutralization titers against authentic LASV (lineage IV) by LAVA01, 19.7E, 37.7H, and VRC01 (HIV-1 Env-specific mAb; negative control). The dotted line indicates the upper limit of detection.

(E) Reconstructed 3D map (transparent white surface) and relaxed model (GPC—gray, glycans—golden, LAVA01 heavy chain—purple, LAVA01 light chain—blue) shown in top and side view.

(F) Peptide (turquoise) and glycan (golden) epitope components for LAVA01 antibody.

(G) Overlay of the LAVA01 footprint (transparent light blue circle) with the histidine triad (green) and proposed LAMP-1 receptor-binding site residues determined by mutagenesis (purple).

(H) Representative sensorgram from BLI experiments showing the binding of recombinant LAMP-1 ectodomain to GPC-I53-50A, in the presence (+LAVA01) or absence (−LAVA01) of LAVA01.

(I) Sequence conservation of GPC with the LAVA01 footprint shown (transparent light blue circle).

glycan, through the HCDR2 and HCDR3 loops. The latter explains the strong glycan-dependent neutralization of LAVA01. Even though side chains were not explicitly built, the high amount of tyrosine residues was readily discernible on the LAVA01 heavy chain, mediating interactions with glycan residues, similar to several HIV-1 bNAbs.[36,37] The location of the epitope provides the basis for a potential neutralization mechanism for LAVA01. First, this site is in immediate proximity to the histidine triad consisting of H92, H93, and H230 that is proposed to regulate the onset of the pH-induced conformational change in GP1 that precedes LAMP-1 binding and membrane fusion. Second, mutagenesis studies have identified β5 residues 200–207 as being critical for LAMP-1 receptor binding[38] (Figure 5G). Consistent with the epitope location, LAVA01 competed with LAMP-1 binding in a BLI experiment (Figure 5H). Binding of LAMP-1 was, however, not fully abrogated by LAVA01, which may be explained by the antibody's slight dissociation from GPC at pH 5 (Figure S5C). The putative LAMP-1 binding site features a relatively high sequence conservation across known LASV strains (Figure 5I), consistent with the critical role it has in viral entry. These data are in agreement with the pseudovirus neutralization experiments described above where we observed cross-neutralization of LAVA01 with different lineages of LASV. Collectively, neutralization assays and cryo-EM work showed that we have isolated a relatively broad NAb, elicited after vaccination, that targets a previously undefined site of vulnerability on LASV GPC.

DISCUSSION

Even though the generation of a prefusion-stabilized GPC represents an important advancement for the development of a recombinant protein vaccine, its propensity to dissociate into monomers compromises its potential to induce potent NAb responses.[14,20] The majority of the NAb epitopes require a trimeric GPC, while the exposed interior of monomeric GPCs presumably constitutes a highly immunogenic non-neutralizing epitope as it is the only surface that is not covered by glycans. So far, the detailed *in vitro* and *in vivo* characterization of a soluble prefusion-stabilized trimeric GPC immunogen has not been reported. Here, we showed that fusing GPC to I53-50A facilitates trimer stabilization and enables the formation of NPs presenting native-like trimers. This work allowed us to acquire an antibody-free high-resolution structure of LASV GPC while potentially generating a pipeline for solving GPC structures from other LASV lineages as well as antibody-complexed structures. An immunogenicity study in rabbits demonstrated that GPC-I53-50NPs can induce strong humoral immune responses, including neutralizing responses. In addition, we showed that these NP immunogens can protect guinea pigs from LASV-induced mortality. Furthermore, isolation of mAbs from immunized rabbits revealed that these NPs are able to elicit antibody responses with broad neutralizing potency that target a previously unreported epitope on the putative LAMP-1 binding site.

The absence of the trimer-stabilizing helical bundle that is positioned in the center of most viral type 1 fusion glycoproteins, as well as the large cavities in the trimeric interface, likely explains the intrinsic instability of recombinantly expressed prefusion GPC trimers.[14,21] Fusion to I53-50A did not strengthen the relatively weak interactions between the GPC protomers but functioned essentially as a trimerization domain. This allowed GPC to be expressed at feasible yields while improving antigenicity. Even though EM studies clearly demonstrated that fusion to I53-50A facilitates the generation of native-like GPC trimers, there is still room for improvement. Splayed open GPC trimers kept together by I53-50A were visible in the 2D-class averages, which became more abundant when complexed with LAVA01 Fab. Additional stabilization strategies might be necessary to further improve trimerization. We showed here that chemically cross-linking GPC-I53-50A is a promising avenue. Alternatively, or concomitantly, shortening or rigidifying the linker between GPC and the trimeric scaffold may represent straightforward approaches to further stabilize trimers. Indeed, GPC-I53-dn5B, which we generated during this study for serum ELISAs and contained only a 3-amino acid linker, showed a much higher trimeric homogeneity than GPC-I53-50A by nsEM.

Three immunizations with GPC-I53-50A were necessary to induce pseudovirus neutralization in a subset of animals, which increased to potent NAb titers after the final and fourth boost. Presentation on I53-50NPs improved GPC's ability to induce NAb responses with only 1 rabbit showing no neutralization after four immunizations. In addition, we also observed that GPC-I53-50NPs induced heterologous neutralization to lineages II and III pseudovirus, even in the context of low autologous NAb titers. Moreover, sera from GPC-I53-50NP recipients, in contrast to those from animals immunized with trimer only, were able to neutralize a lineage V pseudovirus albeit with low potency. Consistent with our study, four immunizations with a LASV GPC-VLP were required to induce potent and broad neutralization, with NAb titers being the lowest to lineage V.[39] However, despite potent pseudovirus neutralization in some animals, authentic LASV neutralization was considerably weaker. The large difference in sensitivity between the two assays stresses the importance of including assays that use authentic LASV when assessing NAb responses. The absence of a NAb response in the immunized guinea pigs was surprising. This may relate to the overall weaker responses, differences in the B cell repertoire, or to other factors. Further stabilization of GPC, by aforementioned or alternative trimer engineering strategies, may improve the immunogenicity of these NP immunogens. Increasing the overall immunogenicity of GPC NPs might also be necessary.

Monoclonal Ab isolation yielded LAVA01, which neutralized lineage IV pseudovirus as well as lineages II and III, the two lineages that currently dominate LASV outbreaks in Nigeria.[40] The neutralization potency against these pseudoviruses was similar to that of the GP1-targeting human NAb 19.7E. Interestingly, the bNAb 37.7H was unable to neutralize lineage III pseudovirus in our hands. This is in contrast to previous results with the same pseudovirus assay,[19] but consistent with other reports.[39] Furthermore, whereas 19.7E was previously unable to neutralize lineage III,[19] it did neutralize our lineage III pseudovirus. These results may be explained by the sequence variation between the different lineage III strains used and highlight the need for a standardized panel of reference strains for assessment of neutralization breath. The relatively broad neutralizing phenotype of LAVA01 may be the result of this mAb targeting highly

CellPress
OPEN ACCESS

conserved residues on the LAMP-1 binding site and making extensive contacts with the conserved glycans at sites N109 and N390. The dependency of pseudovirus neutralization on glycan N390 is an interesting feature as it reveals that the glycan shield may not only shield GPC from NAb responses but can also be a target, akin to the glycan shield of HIV-1 Env.[41,42] The epitope of LAVA01 represents a new target to be exploited by vaccines and antibody therapeutics.

The relevance of NAbs as a protective endpoint for LASV vaccines has only recently been made clear through the success of several passive immunization studies with monoclonal bNAbs in animal models.[10–12] Yet, various studies have also shown protection to LASV challenge in the absence of measurable (pseudovirus) NAb responses, suggesting a role for cellular immunity or non-neutralizing Ab effector functions in vaccine-induced protection.[33,34,43] Our guinea pig study provides additional evidence that NAb responses may not be the sole determinant for protection. However, GPC-I53-50NP's inability to prevent illness and fully protect against a lethal LASV challenge in guinea pigs suggests that GPC-I53-50NP's efficacy may be improved when able to induce a potent NAb response. This study represents a step forward in generating a LASV vaccine that induces strong and protective humoral immunity.

STAR★METHODS

Detailed methods are provided in the online version of this paper and include the following:

- KEY RESOURCES TABLE
- RESOURCE AVAILABILITY
  - Lead contact
  - Materials availability
  - Data and code availability
- EXPERIMENTAL MODEL AND SUBJECT DETAILS
  - Cell lines
  - Rabbits
  - Guinea pigs
- METHOD DETAILS
  - Construct design
  - Protein expression and purification
  - GPC-I53-50NP assembly
  - BN-PAGE and SDS-PAGE analysis
  - Negative stain electron microscopy
  - Bio-layer interferometry
  - LAMP-1 competition bio-layer interferometry assay
  - Differential scanning fluorimetry
  - Site-specific glycan analysis
  - Dynamic light scattering
  - Preparation of LASV glycoprotein immune complex with LAVA01 Fab
  - CryoEM grid preparation and imaging
  - CryoEM data processing
  - Model building and refinement
  - Rabbit immunizations
  - Guinea pig immunizations and challenge
  - Focus-forming assay
  - Serum antibody ELISA
  - Luminex assay for GPC-specific IgM
  - Antibody-dependent NK activation assay
  - Antibody-dependent cellular phagocytosis assay
  - B cell sorting
  - Antibody cloning
  - Fab preparation
  - Monoclonal antibody ELISA
  - Generation of LASV pseudovirus
  - Pseudovirus neutralization assay
  - Neutralization assay using authentic LASV
- QUANTIFICATION AND STATISTICAL ANALYSIS

SUPPLEMENTAL INFORMATION

Supplemental information can be found online at https://doi.org/10.1016/j.chom.2022.10.018.

ACKNOWLEDGMENTS

We thank Robin Shattock and Paul McKay at Imperial College London for kindly sharing the full-length lineage IV (Josiah) GPC vector, Dietmar Katingen and Philipp Mundsperger at Polymun Scientific for providing the SE adjuvant, Rashmi Ravichandran and Neil King at the Institute for Protein Design for sharing purified I53-50B, and Thijn Brummelkamp for sharing an Fc-tagged LAMP-1 expression plasmid. We thank Gotthard Ludwig and Sebastian Schmidt from the biosafety level 4 facility at the Philipps-University of Marburg for technical assistance. The authors also thank Bill Anderson and Hannah Turner from The Scripps Research Institute for their help with electron microscopy experiments. The rabbit and guinea pig image in Figure 4 was created with BioRender.com. This work was supported by a Rubicon fellowship from the Netherlands Organisation for Scientific Research (#452020226 NWO; to P.J.M.B.), the Vici fellowship from the Netherlands Organisation for Scientific Research (NWO; to R.W.S.), the Fondation Dormeur, Vaduz (to R.W.S. and to M.J.v.G.), and the Bill and Melinda Gates Foundation through grant OPP1170236 (to A.B.W.). A.A. was supported by the amfAR Mathilde Krim Fellowship in Biomedical Research (#110182-69-RKVA). T.S. received funding from the Deutsche Forschungsgemeinschaft (DFG, German Research Foundation)—projektnummer 197785619/SFB1021. M. Crispin is supported by the International AIDS Vaccine Initiative (IAVI) through the Collaboration for AIDS Vaccine Discovery (CAVD) grant, INV-008352/OPP1153692 funded by the Bill and Melinda Gates Foundation.

AUTHOR CONTRIBUTIONS

P.J.M.B., A.A., A.J.R., H.M.-K., T.S., A.B., A.B.W., and R.W.S. conceived and designed experiments. P.J.M.B., A.A., A.J.R., H.M.-K., Y.W., M. Claireaux, H.R.P., T.P.L.B., M.G., J.C.U., A.I.S., J.A.B., K.T., N.M.L., and T.H.S. performed the experiments. P.J.M.B., A.A., A.J.R., H.M.-K., Y.W., M. Claireaux, M.M.v.H., S.W.d.T., and M.J.v.G. set up experimental assays. P.J.M.B., A.A., A.J.R., H.M.-K, Y.W., M. Claireaux, M. Crispin, A.B.W., and R.W.S. analyzed and interpreted data. K.S. shared the HCV E1E2-I53-50A reagent for FACS sorting. P.J.M.B., A.A., A.J.R., H.M.-K., Y.W., M.G., J.C.U., A.I.S., T.S., A.B.W., and R.W.S. wrote the manuscript with input from all authors.

DECLARATION OF INTERESTS

Y.W. has taken up a position at AstraZeneca; all experimental work was performed prior to this development.

INCLUSION AND DIVERSITY

We support inclusive, diverse, and equitable conduct of research.

Received: April 8, 2022
Revised: September 7, 2022
Accepted: October 25, 2022
Published: November 17, 2022

REFERENCES

1. Günther, S., and Lenz, O. (2004). Lassa virus. Crit. Rev. Clin. Lab. Sci. *41*, 339–390. https://doi.org/10.1080/10408360490497456.
2. Dan-Nwafor, C.C., Ipadeola, O., Smout, E., Ilori, E., Adeyemo, A., Umeokonkwo, C., Nwidi, D., Nwachukwu, W., Ukponu, W., Omabe, E., et al. (2019). A cluster of nosocomial Lassa fever cases in a tertiary health facility in Nigeria: description and lessons learned, 2018. Int. J. Infect. Dis. *83*, 88–94. https://doi.org/10.1016/j.ijid.2019.03.030.
3. Fisher-Hoch, S.P., Tomori, O., Nasidi, A., Perez-Oronoz, G.I., Fakile, Y., Hutwagner, L., and McCormick, J.B. (1995). Review of cases of nosocomial Lassa fever in Nigeria: the high price of poor medical practice. BMJ *311*, 857–859. https://doi.org/10.1136/bmj.311.7009.857.
4. Andersen, K.G., Shapiro, B.J., Matranga, C.B., Sealfon, R., Lin, A.E., Moses, L.M., Folarin, O.A., Goba, A., Odia, I., Ehiane, P.E., et al. (2015). Clinical sequencing uncovers origins and evolution of Lassa virus. Cell *162*, 738–750. https://doi.org/10.1016/j.cell.2015.07.020.
5. McCormick, J.B., Webb, P.A., Krebs, J.W., Johnson, K.M., and Smith, E.S. (1987). A prospective study of the epidemiology and ecology of Lassa fever. J. Infect. Dis. *155*, 437–444. https://doi.org/10.1093/infdis/155.3.437.
6. Kenmoe, S., Tchatchouang, S., Ebogo-Belobo, J.T., Ka'e, A.C., Mahamat, G., Guiamdjo Simo, R.E., Bowo-Ngandji, A., Demeni Emoh, C.P., Che, E., Tchami Ngongang, D., et al. (2020). Systematic review and meta-analysis of the epidemiology of Lassa virus in humans, rodents and other mammals in sub-Saharan Africa. PLoS Negl. Trop. Dis. *14*, e0008589. https://doi.org/10.1371/journal.pntd.0008589.
7. Bagcchi, S. (2020). Lassa fever outbreak continues across Nigeria. Lancet Infect. Dis. *20*, 543. https://doi.org/10.1016/S1473-3099(20)30299-1.
8. Ibukun, F.I. (2020). Inter-lineage variation of Lassa virus glycoprotein epitopes: a challenge to Lassa virus vaccine development. Viruses *12*, 386. https://doi.org/10.3390/v12040386.
9. Ehichioya, D.U., Dellicour, S., Pahlmann, M., Rieger, T., Oestereich, L., Becker-Ziaja, B., Cadar, D., Ighodalo, Y., Olokor, T., Omomoh, E., et al. (2019). Phylogeography of Lassa virus in Nigeria. J. Virol. *93*, e00929-19. https://doi.org/10.1128/JVI.00929-19.
10. Mire, C.E., Cross, R.W., Geisbert, J.B., Borisevich, V., Agans, K.N., Deer, D.J., Heinrich, M.L., Rowland, M.M., Goba, A., Momoh, M., et al. (2017). Human-monoclonal-antibody therapy protects nonhuman primates against advanced Lassa fever. Nat. Med. *23*, 1146–1149. https://doi.org/10.1038/nm.4396.
11. Cross, R.W., Hastie, K.M., Mire, C.E., Robinson, J.E., Geisbert, T.W., Branco, L.M., Ollmann Saphire, E., and Garry, R.F. (2019). Antibody therapy for Lassa fever. Curr. Opin. Virol. *37*, 97–104. https://doi.org/10.1016/j.coviro.2019.07.003.
12. Cross, R.W., Mire, C.E., Branco, L.M., Geisbert, J.B., Rowland, M.M., Heinrich, M.L., Goba, A., Momoh, M., Grant, D.S., Fullah, M., et al. (2016). Treatment of Lassa virus infection in outbred guinea pigs with first-in-class human monoclonal antibodies. Antiviral Res. *133*, 218–222. https://doi.org/10.1016/j.antiviral.2016.08.012.
13. Katz, M., Weinstein, J., Eilon-Ashkenazy, M., Gehring, K., Cohen-Dvashi, H., Elad, N., Fleishman, S.J., and Diskin, R. (2022). Structure and receptor recognition by the Lassa virus spike complex. Nature *603*, 174–179. https://doi.org/10.1038/s41586-022-04429-2.
14. Hastie, K.M., Zandonatti, M.A., Kleinfelter, L.M., Heinrich, M.L., Rowland, M.M., Chandran, K., Branco, L.M., Robinson, J.E., Garry, R.F., and Saphire, E.O. (2017). Structural basis for antibody-mediated neutralization of Lassa virus. Science *356*, 923–928. https://doi.org/10.1126/science.aam7260.
15. Jae, L.T., Raaben, M., Herbert, A.S., Kuehne, A.I., Wirchnianski, A.S., Soh, T.K., Stubbs, S.H., Janssen, H., Damme, M., Saftig, P., et al. (2014). Virus entry. Lassa virus entry requires a trigger-induced receptor switch. Science *344*, 1506–1510. https://doi.org/10.1126/science.1252480.
16. Guebre-Xabier, M., Patel, N., Tian, J.-H., Zhou, B., Maciejewski, S., Lam, K., Portnoff, A.D., Massare, M.J., Frieman, M.B., Piedra, P.A., et al. (2020). NVX-CoV2373 vaccine protects cynomolgus macaque upper and lower airways against SARS-CoV-2 challenge. Vaccine *38*, 7892–7896. https://doi.org/10.1016/j.vaccine.2020.10.064.
17. Pauthner, M.G., Nkolola, J.P., Havenar-Daughton, C., Murrell, B., Reiss, S.M., Bastidas, R., Prévost, J., Nedellec, R., von Bredow, B., Abbink, P., et al. (2019). Vaccine-induced protection from homologous tier 2 SHIV challenge in nonhuman primates depends on serum-neutralizing antibody titers. Immunity *50*. 241–252.e6. https://doi.org/10.1016/j.immuni.2018.11.011.
18. McLellan, J.S., Chen, M., Joyce, M.G., Sastry, M., Stewart-Jones, G.B.E., Yang, Y., Zhang, B., Chen, L., Srivatsan, S., Zheng, A., et al. (2013). Structure-based design of a fusion glycoprotein vaccine for respiratory syncytial virus. Science *342*, 592–598. https://doi.org/10.1126/science.1243283.
19. Robinson, J.E., Hastie, K.M., Cross, R.W., Yenni, R.E., Elliott, D.H., Rouelle, J.A., Kannadka, C.B., Smira, A.A., Garry, C.E., Bradley, B.T., et al. (2016). Most neutralizing human monoclonal antibodies target novel epitopes requiring both Lassa virus glycoprotein subunits. Nat. Commun. *7*, 11544. https://doi.org/10.1038/ncomms11544.
20. Hastie, K.M., Cross, R.W., Harkins, S.S., Zandonatti, M.A., Koval, A.P., Heinrich, M.L., Rowland, M.M., Robinson, J.E., Geisbert, T.W., Garry, R.F., et al. (2019). Convergent structures illuminate features for germline antibody binding and Pan-Lassa virus neutralization. Cell *178*. 1004.e14–1015.e14. https://doi.org/10.1016/j.cell.2019.07.020.
21. Hastie, K.M., and Saphire, E.O. (2018). Lassa virus glycoprotein: stopping a moving target. Curr. Opin. Virol. *31*, 52–58. https://doi.org/10.1016/j.coviro.2018.05.002.
22. Sommerstein, R., Flatz, L., Remy, M.M., Malinge, P., Magistrelli, G., Fischer, N., Sahin, M., Bergthaler, A., Igonet, S., Ter Meulen, J., et al. (2015). Arenavirus glycan shield promotes neutralizing antibody evasion and protracted infection. PLoS Pathog. *11*, e1005276. https://doi.org/10.1371/journal.ppat.1005276.
23. Watanabe, Y., Raghwani, J., Allen, J.D., Seabright, G.E., Li, S., Moser, F., Huiskonen, J.T., Strecker, T., Bowden, T.A., and Crispin, M. (2018). Structure of the Lassa virus glycan shield provides a model for immunological resistance. Proc. Natl. Acad. Sci. USA *115*, 7320–7325. https://doi.org/10.1073/pnas.1803990115.
24. Marcandalli, J., Fiala, B., Ols, S., Perotti, M., de van der Schueren, W., Snijder, J., Hodge, E., Benhaim, M., Ravichandran, R., Carter, L., et al. (2019). Induction of potent neutralizing antibody responses by a designed protein nanoparticle vaccine for respiratory syncytial virus. Cell *176*. 1420–1431.e17. https://doi.org/10.1016/j.cell.2019.01.046.
25. Brouwer, P.J.M., Antanasijevic, A., de Gast, M., Allen, J.D., Bijl, T.P.L., Yasmeen, A., Ravichandran, R., Burger, J.A., Ozorowski, G., Torres, J.L., et al. (2021). Immunofocusing and enhancing autologous tier-2 HIV-1 neutralization by displaying Env trimers on two-component protein nanoparticles. NPJ Vaccines *6*, 24. https://doi.org/10.1038/s41541-021-00285-9.
26. Boyoglu-Barnum, S., Ellis, D., Gillespie, R.A., Hutchinson, G.B., Park, Y.-J., Moin, S.M., Acton, O.J., Ravichandran, R., Murphy, M., Pettie, D., et al. (2021). Quadrivalent influenza nanoparticle vaccines induce broad protection. Nature *592*, 623–628. https://doi.org/10.1038/s41586-021-03365-x.
27. Bale, J.B., Gonen, S., Liu, Y., Sheffler, W., Ellis, D., Thomas, C., Cascio, D., Yeates, T.O., Gonen, T., King, N.P., and Baker, D. (2016). Accurate design of megadalton-scale two-component icosahedral protein complexes. Science *353*, 389–394. https://doi.org/10.1126/science.aaf8818.
28. Brouwer, P.J.M., Brinkkemper, M., Maisonnasse, P., Dereuddre-Bosquet, N., Grobben, M., Claireaux, M., de Gast, M., Marlin, R., Chesnais, V., Diry, S., et al. (2021). Two-component spike nanoparticle vaccine protects macaques from SARS-CoV-2 infection. Cell *184*. 1188–1200.e19. https://doi.org/10.1016/j.cell.2021.01.035.
29. Brouwer, P.J.M., Antanasijevic, A., Berndsen, Z., Yasmeen, A., Fiala, B., Bijl, T.P.L., Bontjer, I., Bale, J.B., Sheffler, W., Allen, J.D., et al. (2019). Enhancing and shaping the immunogenicity of native-like HIV-1 envelope

CellPress
OPEN ACCESS

trimers with a two-component protein nanoparticle. Nat. Commun. *10*, 4272. https://doi.org/10.1038/s41467-019-12080-1.

30. Eichler, R., Lenz, O., Garten, W., and Strecker, T. (2006). The role of single N-glycans in proteolytic processing and cell surface transport of the Lassa virus glycoprotein GP-C. Virol. J. *3*, 41. https://doi.org/10.1186/1743-422X-3-41.
31. White, J.M., Delos, S.E., Brecher, M., and Schornberg, K. (2008). Structures and mechanisms of viral membrane fusion proteins: multiple variations on a common theme. Crit. Rev. Biochem. Mol. Biol. *43*, 189–219. https://doi.org/10.1080/10409230802058320.
32. Pone, E.J., Hernandez-Davies, J.E., Jan, S., Silzel, E., Felgner, P.L., and Davies, D.H. (2022). Multimericity amplifies the synergy of BCR and TLR4 for B cell activation and antibody class switching. Front. Immunol. *13*, 882502. https://doi.org/10.3389/fimmu.2022.882502.
33. Abreu-Mota, T., Hagen, K.R., Cooper, K., Jahrling, P.B., Tan, G., Wirblich, C., Johnson, R.F., and Schnell, M.J. (2018). Non-neutralizing antibodies elicited by recombinant Lassa-rabies vaccine are critical for protection against Lassa fever. Nat. Commun. *9*, 4223. https://doi.org/10.1038/s41467-018-06741-w.
34. Mateo, M., Reynard, S., Journeaux, A., Germain, C., Hortion, J., Carnec, X., Picard, C., Baillet, N., Borges-Cardoso, V., Merabet, O., et al. (2021). A single-shot Lassa vaccine induces long-term immunity and protects cynomolgus monkeys against heterologous strains. Sci. Transl. Med. *13*, eabf6348. https://doi.org/10.1126/scitranslmed.abf6348.
35. Safronetz, D., Rosenke, K., Westover, J.B., Martellaro, C., Okumura, A., Furuta, Y., Geisbert, J., Saturday, G., Komeno, T., Geisbert, T.W., et al. (2015). The broad-spectrum antiviral favipiravir protects guinea pigs from lethal Lassa virus infection post-disease onset. Sci. Rep. *5*, 14775. https://doi.org/10.1038/srep14775.
36. Lee, J.H., Andrabi, R., Su, C.-Y., Yasmeen, A., Julien, J.-P., Kong, L., Wu, N.C., McBride, R., Sok, D., Pauthner, M., et al. (2017). A broadly neutralizing antibody targets the dynamic HIV envelope trimer apex via a long, rigidified, and anionic β-hairpin structure. Immunity *46*, 690–702. https://doi.org/10.1016/j.immuni.2017.03.017.
37. McLellan, J.S., Pancera, M., Carrico, C., Gorman, J., Julien, J.-P., Khayat, R., Louder, R., Pejchal, R., Sastry, M., Dai, K., et al. (2011). Structure of HIV-1 gp120 V1/V2 domain with broadly neutralizing antibody PG9. Nature *480*, 336–343. https://doi.org/10.1038/nature10696.
38. Israeli, H., Cohen-Dvashi, H., Shulman, A., Shimon, A., and Diskin, R. (2017). Mapping of the Lassa virus LAMP1 binding site reveals unique determinants not shared by other old world arenaviruses. PLoS Pathog. *13*, e1006337. https://doi.org/10.1371/journal.ppat.1006337.
39. Müller, H., Fehling, S.K., Dorna, J., Urbanowicz, R.A., Oestereich, L., Krebs, Y., Kolesnikova, L., Schauflinger, M., Krähling, V., Magassouba, N., et al. (2020). Adjuvant formulated virus-like particles expressing native-like forms of the Lassa virus envelope surface glycoprotein are immunogenic and induce antibodies with broadly neutralizing activity. NPJ Vaccines *5*, 71. https://doi.org/10.1038/s41541-020-00219-x.
40. Kafetzopoulou, L.E., Pullan, S.T., Lemey, P., Suchard, M.A., Ehichioya, D.U., Pahlmann, M., Thielebein, A., Hinzmann, J., Oestereich, L., Wozniak, D.M., et al. (2019). Metagenomic sequencing at the epicenter of the Nigeria 2018 Lassa fever outbreak. Science *363*, 74–77. https://doi.org/10.1126/science.aau9343.
41. Seabright, G.E., Doores, K.J., Burton, D.R., and Crispin, M. (2019). Protein and glycan mimicry in HIV vaccine design. J. Mol. Biol. *431*, 2223–2247. https://doi.org/10.1016/j.jmb.2019.04.016.
42. Seabright, G.E., Cottrell, C.A., van Gils, M.J., D'addabbo, A., Harvey, D.J., Behrens, A.-J., Allen, J.D., Watanabe, Y., Scaringi, N., Polveroni, T.M., et al. (2020). Networks of HIV-1 envelope glycans maintain antibody epitopes in the face of glycan additions and deletions. Structure *28*. 897–909.e6. https://doi.org/10.1016/j.str.2020.04.022.
43. Fischer, R.J., Purushotham, J.N., van Doremalen, N., Sebastian, S., Meade-White, K., Cordova, K., Letko, M., Jeremiah Matson, M., Feldmann, F., Haddock, E., et al. (2021). ChAdOx1-vectored Lassa fever vaccine elicits a robust cellular and humoral immune response and protects guinea pigs against lethal Lassa virus challenge. NPJ Vaccines *6*, 32. https://doi.org/10.1038/s41541-021-00291-x.
44. Walls, A.C., Fiala, B., Schäfer, A., Wrenn, S., Pham, M.N., Murphy, M., Tse, L.V., Shehata, L., O'Connor, M.A., Chen, C., et al. (2020). Elicitation of potent neutralizing antibody responses by designed protein nanoparticle vaccines for SARS-CoV-2. Cell *183*. 1367–1382.e17. https://doi.org/10.1016/j.cell.2020.10.043.
45. Goddard, T.D., Huang, C.C., Meng, E.C., Pettersen, E.F., Couch, G.S., Morris, J.H., and Ferrin, T.E. (2018). UCSF ChimeraX: meeting modern challenges in visualization and analysis. Protein Sci. *27*, 14–25. https://doi.org/10.1002/pro.3235.
46. Punjani, A., Rubinstein, J.L., Fleet, D.J., and Brubaker, M.A. (2017). cryoSPARC: algorithms for rapid unsupervised cryo-EM structure determination. Nat. Methods *14*, 290–296. https://doi.org/10.1038/nmeth.4169.
47. Suloway, C., Pulokas, J., Fellmann, D., Cheng, A., Guerra, F., Quispe, J., Stagg, S., Potter, C.S., and Carragher, B. (2005). Automated molecular microscopy: the new Leginon system. J. Struct. Biol. *151*, 41–60. https://doi.org/10.1016/j.jsb.2005.03.010.
48. Zheng, S.Q., Palovcak, E., Armache, J.-P., Verba, K.A., Cheng, Y., and Agard, D.A. (2017). MotionCor2: anisotropic correction of beam-induced motion for improved cryo-electron microscopy. Nat. Methods *14*, 331–332. https://doi.org/10.1038/nmeth.4193.
49. Zhang, K. (2016). Gctf: real-time CTF determination and correction. J. Struct. Biol. *193*, 1–12. https://doi.org/10.1016/j.jsb.2015.11.003.
50. Zivanov, J., Nakane, T., Forsberg, B.O., Kimanius, D., Hagen, W.J., Lindahl, E., and Scheres, S.H. (2018). New tools for automated high-resolution cryo-EM structure determination in RELION-3. eLife *7*, e42166. https://doi.org/10.7554/eLife.42166.
51. Ilca, S.L., Kotecha, A., Sun, X., Poranen, M.M., Stuart, D.I., and Huiskonen, J.T. (2015). Localized reconstruction of subunits from electron cryomicroscopy images of macromolecular complexes. Nat. Commun. *6*, 8843. https://doi.org/10.1038/ncomms9843.
52. Leem, J., Dunbar, J., Georges, G., Shi, J., and Deane, C.M. (2016). ABodyBuilder: automated antibody structure prediction with data-driven accuracy estimation. mAbs *8*, 1259–1268. https://doi.org/10.1080/19420862.2016.1205773.
53. Casañal, A., Lohkamp, B., and Emsley, P. (2020). Current developments in coot for macromolecular model building of electron cryo-microscopy and crystallographic data. Protein Sci. *29*, 1069–1078. https://doi.org/10.1002/pro.3791.
54. Barad, B.A., Echols, N., Wang, R.Y.-R., Cheng, Y., DiMaio, F., Adams, P.D., and Fraser, J.S. (2015). EMRinger: side chain-directed model and map validation for 3D cryo-electron microscopy. Nat. Methods *12*, 943–946. https://doi.org/10.1038/nmeth.3541.
55. Williams, C.J., Headd, J.J., Moriarty, N.W., Prisant, M.G., Videau, L.L., Deis, L.N., Verma, V., Keedy, D.A., Hintze, B.J., Chen, V.B., et al. (2018). MolProbity: more and better reference data for improved all-atom structure validation. Protein Sci. *27*, 293–315. https://doi.org/10.1002/pro.3330.
56. Lander, G.C., Stagg, S.M., Voss, N.R., Cheng, A., Fellmann, D., Pulokas, J., Yoshioka, C., Irving, C., Mulder, A., Lau, P.-W., et al. (2009). Appion: an integrated, database-driven pipeline to facilitate EM image processing. J. Struct. Biol. *166*, 95–102. https://doi.org/10.1016/j.jsb.2009.01.002.
57. Ueda, G., Antanasijevic, A., Fallas, J.A., Sheffler, W., Copps, J., Ellis, D., Hutchinson, G.B., Moyer, A., Yasmeen, A., Tsybovsky, Y., et al. (2020). Tailored design of protein nanoparticle scaffolds for multivalent presentation of viral glycoprotein antigens. eLife *9*, e57659. https://doi.org/10.7554/eLife.57659.
58. Brouwer, P.J.M., Caniels, T.G., van der Straten, K., Snitselaar, J.L., Aldon, Y., Bangaru, S., Torres, J.L., Okba, N.M.A., Claireaux, M., Kerster, G., et al. (2020). Potent neutralizing antibodies from COVID-19 patients define multiple targets of vulnerability. Science *369*, 643–650. https://doi.org/10.1126/science.abc5902.

59. de Taeye, S.W., Ozorowski, G., Torrents de la Peña, A., Guttman, M., Julien, J.-P., van den Kerkhof, T.L.G.M., Burger, J.A., Pritchard, L.K., Pugach, P., Yasmeen, A., et al. (2015). Immunogenicity of stabilized HIV-1 envelope trimers with reduced exposure of non-neutralizing epitopes. Cell *163*, 1702–1715. https://doi.org/10.1016/j.cell.2015.11.056.

60. Antanasijevic, A., Ueda, G., Brouwer, P.J.M., Copps, J., Huang, D., Allen, J.D., Cottrell, C.A., Yasmeen, A., Sewall, L.M., Bontjer, I., et al. (2020). Structural and functional evaluation of de novo-designed, two-component nanoparticle carriers for HIV Env trimer immunogens. PLoS Pathog. *16*, e1008665. https://doi.org/10.1371/journal.ppat.1008665.

61. Wang, R.Y.-R., Song, Y., Barad, B.A., Cheng, Y., Fraser, J.S., and DiMaio, F. (2016). Automated structure refinement of macromolecular assemblies from cryo-EM maps using Rosetta. eLife *5*, e17219. https://doi.org/10.7554/eLife.17219.

62. Romee, R., Foley, B., Lenvik, T., Wang, Y., Zhang, B., Ankarlo, D., Luo, X., Cooley, S., Verneris, M., Walcheck, B., et al. (2013). NK cell CD16 surface expression and function is regulated by a disintegrin and metalloprotease-17 (ADAM17). Blood *121*, 3599–3608. https://doi.org/10.1182/blood-2012-04-425397.

63. Schriek, A.I., van Haaren, M.M., Poniman, M., Dekkers, G., Bentlage, A.E.H., Grobben, M., Vidarsson, G., Sanders, R.W., Verrips, T., Geijtenbeek, T.B.H., et al. (2022). Anti-HIV-1 nanobody-IgG1 constructs with improved neutralization potency and the ability to mediate Fc effector functions. Front. Immunol. *13*, 893648. https://doi.org/10.3389/fimmu.2022.893648.

64. van Haaren, M.M., McCoy, L.E., Torres, J.L., Lee, W., Cottrell, C.A., Copps, J.L., van der Woude, P., Yasmeen, A., de Taeye, S.W., Torrents de la Peña, A., et al. (2021). Antibodies from rabbits immunized with HIV-1 Clade B SOSIP trimers can neutralize multiple Clade B viruses by destabilizing the envelope glycoprotein. J. Virol. *95*, e0009421. https://doi.org/10.1128/JVI.00094-21.

65. McCoy, L.E., van Gils, M.J., Ozorowski, G., Messmer, T., Briney, B., Voss, J.E., Kulp, D.W., Macauley, M.S., Sok, D., Pauthner, M., et al. (2016). Holes in the glycan shield of the native HIV envelope are a target of trimer-elicited neutralizing antibodies. Cell Rep. *16*, 2327–2338. https://doi.org/10.1016/j.celrep.2016.07.074.

66. Bianchi, M., Turner, H.L., Nogal, B., Cottrell, C.A., Oyen, D., Pauthner, M., Bastidas, R., Nedellec, R., McCoy, L.E., Wilson, I.A., et al. (2018). Electron-microscopy-based epitope mapping defines specificities of polyclonal antibodies elicited during HIV-1 BG505 envelope trimer immunization. Immunity *49*. 288–300.e8. https://doi.org/10.1016/j.immuni.2018.07.009.

CellPress
OPEN ACCESS

STAR★METHODS

KEY RESOURCES TABLE

REAGENT or RESOURCE	SOURCE	IDENTIFIER
Antibodies		
19.7E	Robinson et al.[19]	Patent WO2018106712A1
12.1F	Robinson et al.[19]	Patent WO2018106712A1
25.10C	Robinson et al.[19]	Patent WO2018106712A1
37.7H	Robinson et al.[19]	Patent WO2018106712A1
LAVA01	This study	N/A
LAVA02	This study	N/A
LAVA03	This study	N/A
LAVA04	This study	N/A
Goat anti-mouse	Seracare	Cat# 5450-0011
HRP-labeled goat anti-rabbit IgG	Jackson Immunoresearch	Cat# 111-035-144
HRP-labeled donkey anti-guinea pig IgG	Jackson Immunoresearch	Cat# 706-035-148
Donkey anti-rabbit IgG-PE	Biolegend	Cat# 406421
Goat-anti-rabbit IgM-PE	Southern Biotech	Cat# 4020-09
PE-labeled anti-human CD16 antibody	Biolegend	Cat# 302008
Fixable Viability Dye eF780	eBioscience	Cat# 65-0865-14
Bacterial and virus strains		
Lassa virus (Josiah strain)	Philipps-University Marburg	GenBank: P08669
Guinea-pig adapted Lassa virus (Josiah strain)	Safronetz et al.[35]	GenBank: P08669
NEB 5-alpha Competent E. coli (High Efficiency)	New England Biolabs	Cat# C2987H
Biological samples		
Sera immunized rabbits	This study	N/A
Sera immunized guinea pigs	This study	N/A
PBMCs immunized rabbits	This study	N/A
PBMCs naïve humans	Sanquin Blood bank Amsterdam	N/A
Chemicals, peptides, and recombinant proteins		
I53-50B.4PT1	Walls et al.[44]	N/A
IL-15	Thermo Scientific	Cat# BMS319
Tween20	Sigma-Aldrich	Cat# P1379-500ML
Bovine Serum Albumin (fraction V)	Thermo Scientific	Cat# 9048-46-8
Milk powder	PanReac AppliChem	Cat# A0830
1% 3,3′,5,5′-tetranethylbenzidine	Sigma-Aldrich	Cat# 88748
EDTA (Titriplex III)	Sigma-Aldrich	Cat# 1.08418.1000
Saquinavir	NIH-ARP	Cat# 4658
DEAE-dextran	Sigma-Aldrich	Cat# D9885
Mass spectrometry grade trypsin	Promega	Cat# V5280
Sequencing grade chymotrypsin	Promega	Cat# V1061
Alpha lytic protease	Sigma-Aldrich	Cat# A6362
Acetonitrile, 80%, 20% Water with 0.1% Formic Acid, Optima LC/MS	Thermo Scientific	Cat# 15431423
Water with 0.1% Formic Acid (v/v), Optima™ LC/MS Grade	Thermo Scientific	Cat# LS118-212
Acetonitrile	Thermo Scientific	Cat# 10489553
Trifluoroacetic acid	Thermo Scientific	Cat# 10155347
Dithiothreitol	Sigma-Aldrich	Cat# 43819

(*Continued on next page*)

Continued

REAGENT or RESOURCE	SOURCE	IDENTIFIER
Iodacetamide	Sigma-Aldrich	Cat# I1149
Phorbol myristate acetate (PMA)	Sanbio	Cat# P8139-1MG
Urea	Sigma-Aldrich	U5378-1KG
Glu-C	Promega	Cat# V1651
BioLock solution	IBA Lifesciences	Cat# 2-0205-050
Reporter Lysis buffer	Promega	Cat# E3971
PBS	Thermo Scientific	Cat# 10010023
TBS	Alfa Aesar	Cat# J60764.K2
Glutaraldehyde	Sigma-Aldrich	Cat#
PEI MAX	Polysciences	Cat# 24765-1
3,3',5,5'-tetranethylbenzidine	Sigma-Aldrich	Cat# T4444
Squalene Emulsion adjuvant	Polymun Scientific	N/A
AEC chromogen	Enquire Bioreagents	Cat# AB64252
Galanthus Nivalis Lectin	Vector Laboratories	Cat# L-1240-5
Amersham High Molecular Weight marker	Sigma-Aldrich	Cat# 17-0445-01
1-Ethyl-3-(3-dimethylaminopropyl) carbodiimide	Thermo Scientific	Cat# A35391
Sulfo-N-Hydroxysulfosuccinimide	Thermo Scientific	Cat# A39269
Casein buffer	Thermo Scientific	Cat# 37528
Penicillin	Sigma-Aldrich	Cat# P3032-10MI
Streptomycin	VWR	Cat# 382-EU-100G
Penicillin-Streptomycin (5,000 U/mL)	Gibco	Cat# 15070063
Gentamicin	Corning	Cat# 30-005-CR
Fugene	Promega	Cat# E5911
Carboxymethylcellulose	Thermo Scientific	Cat# M352-500
Neutral buffered formalin	Thermo Scientific	Cat# 245-684
Ionomycin	Sanbio	Cat# 10004974-5
Streptavidin BB515	BD	Cat# 564453
Streptavidin AF647	Biolegend	Cat# 405237
Streptavidin BV421	Biolegend	Cat# 405226
Lauryl maltose neopentyl glycol	Anatrace	Cat# NG310
Uranyl Formate	Electron Microscopy Sciences	Cat #D310 25 GM
**Critical commercial assays**		
Bright-Glo Luciferase Assay System	Promega	Cat# E2620
**Deposited data**		
3D map of GPCysR4(Josiah) from localized reconstruction of GPCysR4(Josiah)-I53-50NP	This paper	EMDB: EMD-25107
3D model of GPCysR4(Josiah) from localized reconstruction of GPCysR4(Josiah)-I53-50NP	This paper	PDB: 7SGD
3D map of I53-50NP from focused refinement of GPCysR4(Josiah)-I53-50NP	This paper	EDMB: EMD-25108
3D model of I53-50NP from focused refinement of GPCysR4(Josiah)-I53-50NP	This paper	PDB: 7SGE
3D map of GPCysR4(Josiah)-I53-50A.1NT1 in complex with LAVA01 antibody	This paper	EMDB: EMD-25109
3D model of GPCysR4(Josiah)-I53-50A.1NT1 in complex with LAVA01 antibody	This paper	PDB: 7SGF
**Experimental models: Cell lines**		
FreeStyle 293F cells	Thermo Scientific	Cat# R79007
HEK 293T cells	ATCC	Cat# CRL-11268
Vero cells	ATCC	Cat# CCL-81

(*Continued on next page*)

Continued		
REAGENT or RESOURCE	SOURCE	IDENTIFIER
Vero E6 cells	ATCC	Cat# CRL-i586
TZM-bl cells	NIH ARRRP	Cat# 8129
THP-1 cells	ATCC	Cat# TIB-202
Experimental models: Organisms/strains		
New Zealand White rabbits	Covance Research Products, Inc	N/A
Hartley Guinea Pigs	UTMB	N/A
Recombinant DNA		
GPCysR4(Josiah)-StreptagII pPPI4 plasmid	This study	N/A
GPCysR4(Josiah)-I53-50A.1NT1-Strep-tagII pPPI4 plasmid	This study	N/A
GPCysR4(Josiah)-I53-dn5B-StreptagII pPPI4 plasmid	This study	N/A
GPCysR4(Josiah)-I53-50A.1NT1-Avi-His pPPI4 plasmid	This study	N/A
GPCysR4(NIG08-A41)-I53-50A.1NT1-Avi-His pPPI4 plasmid	This study	N/A
Furin pPPI4 plasmid	Brouwer et al.[29]	N/A
Fc-tagged LAMP-1 ectodomain	Jae et al.[15]	N/A
19.7E HC, 19.7E LC, 12.1F HC, 12.1F LC, 25.10C HC, 25.10C LC, 37.7H HC, 37.7H LC gene fragments	Integrated DNA Technologies	N/A
Software and algorithms		
Dynamics	Wyatt Technology Corporation	N/A
ByosTM (Version 4.0)	Protein Metrics Inc.	https://www.proteinmetrics.com/products/byonic/
GraphPad Prism v8	GraphPad	N/A
XCalibur Version v4.2	Thermo Scientific	N/A
Orbitrap Fusion Tune application v3.1	Thermo Scientific	N/A
FlowJo v.10.7.1	Flowjo	N/A
UCSF ChimeraX	Goddard et al.[45]	N/A
cryoSPARC.v2	Punjani et al.[46]	N/A
Leginon	Suloway et al.[47]	N/A
MotionCor2	Zheng et al.[48]	N/A
GCTF	Zhang[49]	N/A
Relion/3.0	Zivanov et al.[50]	N/A
Localized Reconstruction v1.2.0	Ilca et al.[51]	N/A
AbodyBuilder	Leem et al.[52]	N/A
Coot	Casañal et al.[53]	N/A
EMRinger	Barad et al.[54]	N/A
MolProbity	Williams et al.[55]	N/A
Appion	Lander et al.[56]	N/A
Other		
EasySpray PepMap RSLC C18 column (75 µm x 75 cm)	Thermo Scientific	Cat# ES805
Fetal bovine serum	R&D Biosystems	Cat# S11150H
Fetal calf serum	Gibco	Cat# 10270/106
C18 ZipTip	Merck Milipore	Cat# ZTC18S008
Immobilized papain resin	Thermo Scientific	Cat# 20341
Protein A resin	Thermo Scientific	Cat# 20333
CD56 MicroBeads, human	Miltenyi Biotec	Cat# 130-050-401
CaptureSelect IgG-Fc resin	Thermo Scientific	Cat# 2942852010
FluoSpheres NeutrAvidin-Labeled Microspheres	Thermo Scientific	Cat# F8776
Vivaspin 6, 10.000 Da MWCO, polyethersulfone	Sigma-Aldrich	Cat# GE28-9322-96
Vivaspin 20, 50.000 Da MWCO, polyethersulfone	Sigma-Aldrich	Cat# GE28-9323-62

(*Continued on next page*)

Continued

REAGENT or RESOURCE	SOURCE	IDENTIFIER
Vivaspin 500, 3.000 Da MWCO, polyethersulfone	Sigma-Aldrich	Cat# GE28-9322-18
Amicon 15, 10.000 Da MWCO, cellulose	Sigma-Aldrich	Cat# UFC901024
Slide-A-Lyzer Mini dialysis device 10 kDa MWCO	Thermo Scientific	Cat# 88401
PepMap100 C18 3UM 75UMx2CM Nanoviper	Thermo Scientific	Cat# 164946
Ni-NTA agarose	QIAGEN	Cat# 30210
Novex Wedgewell 10-20% Tris-Glycine gels	Thermo Scientific	Cat# XP10202BOX
NuPAGE 4-12% Bis-Tris gels	Thermo Scientific	Cat# NP0321BOX
Superose 6 increase 10/300 GL	Sigma-Aldrich	Cat# GE29-0915-96
HiLoad 16/600 Superdex pg200	Sigma-Aldrich	Cat# GE28-9893-35
Econo-column chromatography columns	BIO RAD	Cat# 7371512
NGC chromatography system	BIO RAD	N/A
Octet K2 system	Sartorius (ForteBio)	N/A
Octet Biosensors: Streptavidin	Sartorius (ForteBio)	Cat# 18-5019
Octet Biosensors: Protein A	Sartorius (ForteBio)	Cat# 18-5010
Octet Biosensors: Anti-Human Fc Capture (AHC)	Sartorius (ForteBio)	Cat# 18-5060
Nucleobond Xtra Maxi kit	Macherey-Nagel	Cat# 740414.50
Fast Digest BamHI	Thermo Scientific	Cat# FD0054
Fast Digest Green buffer 10x	Thermo Scientific	Cat# B72
Fast Digest PstI	Thermo Scientific	Cat# FD0614
Fast Digest NotI	Thermo Scientific	Cat# FD0593
Random Hexamer Primers	Thermo Scientific	Cat# SO142
dNTP mix	New England Biolabs	Cat# N0447S
HotStarTaq Plus DNA polymerase	Qiagen	Cat# 203603
Biotin protein ligase	GeneCopoeia	Cat# BI001
T5 exonuclease	New England Biolabs	Cat# M0363
Taq DNA ligase	New England Biolabs	Cat# M0208S
NAD^+	New England Biolabs	Cat# B9007S
Superscript III RTase	Thermo Fischer	Cat# 18080044
Phusion High-Fidelity DNA Polymerase	New England Biolabs	Cat# M0530S
Magplex Microspheres	Luminex	Cat# MC10065-01
FreeStyle 293 Expression medium	Thermo Scientific	Cat# 12338018
Ficoll-Paque™ PLUS	Sigma-Aldrich	Cat# GE17-1440-02
DMEM	Gibco	Cat# 11965092
OptiMEM	Gibco	Cat# 31985-070
Iscove's Modified Dulbecco's Medium	Gibco	Cat# 12440053
RPMI 1640 Medium	Gibco	Cat# 11875093
Glutamax supplement	Thermo Scientific	Cat# 35050061
L-Glutamine	Gibco	Cat# 25030149
High-binding plates: Half-area 96-well polystyrene high-binding microplate	Greiner	Cat# 675061
Steritop Filter Units	Merck Millipore	Cat# C3239
QuantiFoil R 2/1 grids (400-mesh)	Thermo Scientific	Cat# 50-190-2583
UltrAuFoil R 1.2/1.3 grids (300-mesh)	Quantifoil Micro Tools GmbH	N/A
Glomax reader	Turner BioSystems	Model# 9101-002
Microplate 96 well half area white	Greiner bio-one	Cat# 675074
FACSymphony A1 Cell Analyzer	BD biosciences	N/A
FACS-ARIA-SORP	BD biosciences	N/A
Greiner CELLSTAR® 96 well plates round bottom clear wells	Merck Millipore	Cat# M9436
Strep-TactinXT Superflow high capacity resin	IBA Life Sciences	Cat# 2-4010-010
NanoDrop 2000C	Thermo Scientific	Cat# ND-2000C

(*Continued on next page*)

Continued

REAGENT or RESOURCE	SOURCE	IDENTIFIER
Prometheus NT.48 NanoDSF	NanoTemper Technologies	N/A
Dynapro Nanostar	Wyatt Technology Corporation	N/A
Orbitrap Eclipse mass spectrometer	Thermo Scientific	N/A
Ultimate 3000 HPLC	Thermo Scientific	N/A
Vitrobot mark IV	Thermo Scientific	N/A
Solarus 950 plasma system	Gatan	N/A
FEI Titan Krios	Thermo Scientific	N/A
K2 Summit direct electron detector camera	Gatan	N/A
MAGPIX system	Luminex	Model# MAGPIX-XPON4.1-CEIVD

RESOURCE AVAILABILITY

Lead contact

Further information and requests for resources and reagents should be directed to and will be fulfilled by the lead contact, Rogier W. Sanders (r.w.sanders@amsterdamumc.nl).

Materials availability

All reagents will be made available on request after completion of a Materials Transfer Agreement.

Data and code availability

- 3D maps from electron microscopy experiments have been deposited to the Electron Microscopy Databank (http://www.emdatabank.org/) under accessions EMDB: EMD-25107, EMD-25108, EMD-25109. 3D models from electron microscopy experiments have been deposited to the Protein Data Bank (http://www.rcsb.org/) under accessions PDB: 7SGD, 7SGE, 7SGF. The raw data reported in this study will be shared by the corresponding authors upon request.
- This paper does not report original code.
- Any additional information required to reanalyze the data reported in this work paper is available from the lead contact upon request.

EXPERIMENTAL MODEL AND SUBJECT DETAILS

Cell lines

FreeStyle293F (Thermo Scientific) and HEK293T (ATCC) are human embryonic kidney cell lines transformed for increased production of recombinant protein or retrovirus. HEK293F cells are adapted to grow in suspension and are cultured at 37°C with 8% CO_2 and shaking at 125 rpm in 293FreeStyle expression medium (Thermo Scientific). HEK 293T cells are maintained in Dulbecco's Modified Eagle's Medium (DMEM; Gibco) supplemented with 10% fetal calf serum (FCS) (Gibco), penicillin (100 U/mL), and streptomycin (100 μg/mL) and grown statically at 37°C with 8% CO_2. The THP-1 (ATCC) cell line is a human monocytic cell line that represents primary monocytes and macrophages. THP-1 cells were cultured at 37°C, 5% CO_2 in RPMI 1640 Medium (Thermo Scientific) supplemented with 10% FCS (Gibco), penicillin (100 U/mL), and streptomycin (100 μg/mL). Cells were passaged 3 times a week to maintain a density of $0.5–1\times10^6$ cells/mL. The TZM-bl cell line is an indicator cell line that is highly sensitive to infection with diverse isolates of HIV-1 and enables quantitative analysis of HIV infection using either ß-galactosidase or luciferase as a reporter. The parental cell line (JC.53) stably expresses large amounts of CD4 and CCR5 and constitutively expresses CXCR4. TZM-bl cells were maintained in DMEM, supplemented with 10% FCS, penicillin (100 U/mL), and streptomycin (100 μg/mL). VeroE6 cells were cultured at 37°C, 5% CO2 in DMEM (Gibco) supplemented with 10% FCS (Gibco), 50 U/mL penicillin-streptomycin (Gibco) and 2 mM L-glutamine (Gibco). Cells were passaged 2 times per week to maintain a density of $1\text{-}1.3\times10^6$ cells/mL.

Rabbits

Female New Zealand White rabbits of 2.5-3 kg from multiple litters were used. Animals were sourced and housed at Covance Research Products, Inc. (Denver, PA, USA) and immunizations were performed under permits with approval number C0096-19. Immunization procedures complied with all relevant ethical regulations and protocols of the Covance Institutional Animal Care and Use Committee.

Guinea pigs

Female Hartley guinea pigs of around 400 g from multiple litters were used. Animals were sourced and housed at UTMB. The guinea pig studies were carried out in accordance with the recommendations per the Guide for the Care and Use of Laboratory Animals of

the National Research Council. UTMB is an AAALAC-accredited institution, and all animal work was approved by the Institutional Animal Care and Use Committee of UTMB under approval number 1911089. All efforts were made to minimize animal suffering and all procedures involving potential pain were performed under general anaesthesia.

METHOD DETAILS

Construct design

To generate the prefusion stabilized GPC construct, a gene encoding residues 1-424 of GPC from the Josiah strain (GenBank: ADY11068.1) with the GPCysR4 mutations (R207C, E329P, L258R, L259R, G360C[14]) following C-terminal extension, GSGSLEWSHPQFEK (GS encodes a BamHI site), was cloned into a PstI-NotI-digested pPPI4 plasmid by Gibson assembly. GPC-I53-50A was created by digesting the GPC plasmid with BamHI and NotI and subsequent Gibson assembly with a gene encoding the previously described I53-50A.1NT1 sequence (including the glycine-serine linker) with a GSLEWSHPQFEK extension on the C-terminus.[28] For the GPC-I53-dn5B construct a gene encoding the recently described I53_dn5B sequence (EEAE...MREE[57]) preceded by a GSG sequence and followed by a GGWSHPQFEK sequence was ordered and cloned into a BamHI-NotI digested GPC-I53-50A plasmid. The lineage IV probe for B cell sorting was generated by Gibson assembly of the previously mentioned prefusion GPC gene into a PstI-BamHI-digested pPPI4 plasmid encoding a I53-50A.1NT1 sequence that had an Avi- and histidine-tag after the final residue. To generate a lineage II probe a gene encoding residues 1-423 of GPC from the NIG08-A41 strain (GenBank: ADU56626) containing the GPCysR4 mutations were introduced. Plasmids encoding the mAbs 19.7E, 37.7H, 12.1F, and 25.10C were generated by ordering genes encoding the variable regions of the corresponding heavy and light chains and cloning them in expression vectors containing the constant regions of the human IgG1 for the heavy or light chain using Gibson assembly. For pseudovirus neutralization assays, pPPI4 plasmid was digested with PstI-NotI and a gene encoding full-length GPC of lineage II (NIG08-A41), lineage III (CSF; GenBank: AAL13212.1), or lineage V (Bamba; GenBank: AHC95555.1) was inserted by Gibson assembly. The N390D and N395D mutants were generated by Q5 site-directed mutagenesis using a plasmid encoding full-length Josiah strain as a template.

Protein expression and purification

Proteins were expressed by transient transfection of HEK 293F cells (0.8-1.2 million cells/mL) maintained in Freestyle medium (Thermo Scientific). A 3:1 ratio of PEImax and expression plasmids (312.5 μg/L cells) were added to the cells. For GPC fusion proteins a 2:1 ratio of GPC-I53-50A/GPC-I53-dn5B and furin was used to ensure optimal furin cleavage. For non-scaffolded GPC this was 3:1. MAbs were transfected using a 1:1 ration of heavy and light chain expression plasmid. After six days, supernatants were harvested by centrifugation (30 min at 4000 rpm) and filtration using a 0.22 μm Steritop filter (Merck Millipore). Supernatants containing mAbs were purified as described previously.[58] GPC constructs were purified using StrepTactinXT Superflow high capacity 50% suspension in accordance with the manufacturer's protocol for gravity flow (IBA Life Sciences). Prior to loading on the column, Biolock solution and 10X buffer W (1 M Tris/HCl, 1.5 M NaCl, 10 mM EDTA, pH 8.0) were diluted 1:1000 and 1:10, respectively, in the filtered supernatant. Eluted GPC constructs were buffer exchanged in Tris-buffer-saline (TBS), supplemented with 5% glycerol, using Vivaspin filters with a 50 kDa molecular weight cutoff (Sigma-Aldrich). GPC-I53-50A for rabbit immunization studies were subjected to an additional SEC step using a Superose 6 increase 10/300 GL column in TBS, 5% glycerol. Fractions between 14 and 16.5 mL were collected, pooled and concentrated using Vivaspin filters with a 50 kDa molecular weight cutoff (Sigma-Aldrich). To generate recombinant LAMP-1, 312.5 μg of a rabbit Fc-tagged LAMP-1 plasmid (encoding residues A29-S351; a kind gift from Thijn Brummelkamp[15]), were transfected in HEK 293F cells. LAMP-1 was purified from culture supernatant using CaptureSelect IgG-Fc resin (Thermo Scientific) over a gravity flow column. Elution was performed with 0.1 M glycine, pH 3.0, into the neutralization buffer 1 M Tris, pH 8.0, at a 1:9 ratio. Purified LAMP-1 was then buffer exchanged to TBS, pH 8.0, using Vivaspin filters with a 10 kDa molecular weight cutoff (Sigma-Aldrich). The Nanodrop was used to determine the concentrations of expressed proteins applying the proteins peptidic molecular weight and extinction coefficient as calculated by the online ExPASy software (ProtParam).

GPC-I53-50NP assembly

GPC-I53-50NPs were generated by collecting the previously mentioned SEC fractions of non-aggregated GPC-I53-50A (14 - 16.5 mL) and adding I53-50B.4PT1 pentamer (expressed as described previously[44]) at an equimolar amount of monomeric subunits. After an overnight incubation at 4°C, the mix was run over a Superose 6 increase 10/300 GL column in TBS, 5% glycerol to remove non-assembled components. Fractions between 8.5 and 10 mL were pooled and concentrated by centrifugation at 350 x *g* using Vivaspin filters with a 10 kDa molecular weight cutoff (Sigma-Aldrich). Concentrated GPC-I53-50NPs were then diluted 1:1 with TBS, 5% glycerol, 400mM glycine, and the protein concentration was determined by Nanodrop method using the peptidic molecular weight and extinction coefficient as calculated by the online ExPASy software (ProtParam).

BN-PAGE and SDS-PAGE analysis

BN-PAGE and SDS-PAGE were performed as described previously.[59] Briefly, for BN-PAGE analysis, 2.5 μg of GPC or GPC-I53-50A was mixed with loading dye and run on a 4%–12% Bis-Tris NuPAGE gel (Thermo Scientific). For SDS-PAGE analysis, 2.5 ug of GPC-I53-50A or GPC-I53-50NP were mixed with loading buffer in the presence or absence of dithiothreitol and denatured, before loading on a 10-20% Tris-Glyine gel (Thermo Scientific).

Negative stain electron microscopy

Negative stain electron microscopy experiments were performed as described previously.[60] GPC, GPC-I53-50A and GPC-I53-50NP samples were diluted to 10-20 μg/mL, and 3 μL were loaded onto carbon-coated Cu grids (400-mesh). Prior to sample application the grids were glow-discharged at 15 mA for 25 s. Following a 10 s incubation period the samples were blotted off and the grids were negatively stained with 3 μL of 2% (w/v) uranyl-formate solution for 60 s. The grids were imaged on a Tecnai Spirit electron microscope (operating at 120 keV, nominal magnification was 52,000 X, resulting pixel size at the specimen plane was 2.05 Å). Electron dose was set to 25 $e^-/Å^2$ and the nominal defocus for imaging was -1.50 μm. Micrographs were recorded with a Tietz 4k x 4k TemCam-F416 CMOS camera. For data acquisition we used Leginon automated imaging software.[47] The Appion data processing suite[56] was applied for all processing steps (particle picking, extraction and 2D classification).

Bio-layer interferometry

The mAbs 19.7E, 12.1F, 37.7H or 25.10C diluted in running buffer (PBS, 0.02% Tween20, 0.1% BSA) were loaded on a Protein A sensor (Sartorius) to a signal of 1.0 nm using an Octet K2 system (ForteBio). After removal of excess mAb by a short dip in running buffer the sensor was dipped for 200 s in a concentration of 100 nM GPC, GPC-I53-50A or 5 nM of GPC-I53-50NP, diluted in running buffer. After this association step, the sensor was dipped for 200 s in running buffer to measure protein dissociation. The same procedure was used for the comparison of GPC-I53-50A with GPC-I53-dn5B except that Anti-Human Fc Capture (AHC) sensors (Sartorius) were used, the sensors were loaded to a signal of 1.5 nm, and a 200 nM concentration of GPC-I53-50A or GPC-I53-dn5B was used. To analyze the binding kinetics of LAVA01, biotinylated GPC-I53-50A in running buffer was immobilized on a Streptavidin sensor (Sartorius) to a signal of 1.5 nm. After removal of excess GPC-I53-50A by a short dip in running buffer, the sensor was dipped for 600 s in a concentration of 240, 80, 26.7 or 8.89 nM of LAVA01 Fab or a well containing only running buffer (background well). The sensor was then dipped in running buffer for 600 s to measure Fab dissociation. Binding kinetics were determined for background-subtracted data using the ForteBio Data Analysis 9.0 tool. Curve fitting was performed assuming a 1:1 model and on-rate, off-rate, and K_D values were determined with a global fit.

LAMP-1 competition bio-layer interferometry assay

Biotinylated GPC-I53-50A diluted in running buffer (PBS, 0.02% Tween20, 0.1% BSA) were loaded on a Streptavidin sensor (Sartorius) to a signal of 1.0 nm using an Octet K2 system (ForteBio). After removal of excess mAb by a short dip in running buffer the sensor was dipped for 600 s in a concentration of 400 nM LAVA01 diluted in running buffer, or just running buffer alone. After this association step, the sensor was dipped for 1200 s in pH 5.0 running buffer (50 mM NaCitrate, 150 mM NaCl, pH 5.0, 0.02% Tween20, 0.1% BSA) to measure protein dissociation. Next, the sensor was dipped for 600 s in 200 μg/mL of recombinant LAMP-1 ectodomain in pH 5.0 running buffer, after which the sensor was dipped in pH 5.0 running buffer for 1200 s to measure LAMP-1 dissociation.

Differential scanning fluorimetry

Prometheus NT.48 NanoDSF instrument (NanoTemper Technologies) was used for the DSF experiments, as described previously.[60] GPC, GPC-I53-50A and GPC-I53-50NP samples were diluted to 1 mg/mL in the TBS buffer (Alfa Aesar) and ~10 μL of each diluted sample (in duplicates) was loaded into NanoDSF capillaries (NanoTemper Technologies). The temperature was raised from 20°C to 95°C at 1°C/min rate. The T_m value was determined from the first derivative curves using the NT.48 NanoDSF instruments software. The average value from the duplicate measurements is reported as the T_m value in the manuscript.

Site-specific glycan analysis

Three 30 μg aliquots of GPC-I53-50A were denatured for 1h in 50 mM Tris/HCl, pH 8.0 containing 6 M of urea and 5 mM dithiothreitol (DTT). Next, the glycoproteins were reduced and alkylated by adding 20 mM iodoacetamide (IAA) and incubated for 1h in the dark, followed by a 1h incubation with 20 mM DTT to eliminate residual IAA. The alkylated glycoproteins were buffer-exchanged into 50 mM Tris/HCl, pH 8.0 using Vivaspin columns with a 3 kDa molecular weight cutoff and digested separately overnight using trypsin chymotrypsin or Glu-C (Mass Spectrometry Grade, Promega) at a ratio of 1:30 (w/w). The next day, the peptides were dried and extracted using C18 Zip-tip (Merck Milipore). The peptides were dried again, re-suspended in 0.1% formic acid and analyzed by nanoLC-ESI MS with an Easy-nLC 1200 (Thermo Scientific) system coupled to a Fusion mass spectrometer (Thermo Scientific) using higher energy collision-induced dissociation (HCD) fragmentation. Peptides were separated using an EasySpray PepMap RSLC C18 column (75 μm × 75 cm). A trapping column (PepMap 100 C18 3μM 75μM x 2cm) was used in line with the LC prior to separation with the analytical column. The LC conditions were as follows: 275 minute linear gradient consisting of 0-32% acetonitrile in 0.1% formic acid over 240 minutes followed by 35 minutes of 80%acetonitrile in 0.1% formic acid. The flow rate was set to 200 nL/min. The spray voltage was set to 2.7 kV and the temperature of the heated capillary was set to 40°C. The ion transfer tube temperature was set to 275°C. The scan range was 400−1600 m/z. The HCD collision energy was set to 50%, appropriate for fragmentation of glycopeptide ions. Precursor and fragment detection were performed using an Orbitrap at a resolution MS1=100,000. MS2=30,000. The AGC target for MS1=4e5 and MS2=5e4 and injection time: MS1=50ms MS2=54ms.

Glycopeptide fragmentation data were extracted from the raw file using ByonicTM (Version 3.5) and ByologicTM software (Version 3.5; Protein Metrics Inc.). The glycopeptide fragmentation data were evaluated manually for each glycopeptide; the peptide was scored as true-positive when the correct b and y fragment ions were observed along with oxonium ions corresponding to the glycan identified. The MS data was searched using the Protein Metrics N-glycan library. The relative amounts of each glycan at each site as

well as the unoccupied proportion were determined by comparing the extracted chromatographic areas for different glycotypes with an identical peptide sequence. All charge states for a single glycopeptide were summed. The precursor mass tolerance was set at 4 ppm and 10 ppm for fragments. A 1% false discovery rate (FDR) was applied. The relative amounts of each glycan at each site as well as the unoccupied proportion were determined by comparing the extracted ion chromatographic areas for different glycopeptides with an identical peptide sequence. Glycans were categorized according to the composition detected. HexNAc(2)Hex(9−5) was classified as M9 to M5. HexNAc(3)Hex(5−6)(X) was classified as Hybrid with HexNAc(3)Fuc(1)(X) classified as Fhybrid. Complex-type glycans were classified according to the number of processed antenna and fucosylation, as HexNAc(3)(X), HexNAc(3)(F)(X), HexNAc(4)(X), HexNAc(4)(F)(X), HexNAc(5)(X), HexNAc(5)(F)(X), HexNAc(6+)(X), HexNAc(6+)(F)(X).

Dynamic light scattering

DLS was used to assess the hydrodynamic radius (R_h) and polydispersity of the assembled SOSIP-I53-50NPs. The particles were diluted to 0.025 μg/mL in PBS and loaded into a Dynapro Nanostar instrument (Wyatt Technology Corporation). R_h and polydispersity values were measured with ten acquisitions of 5 s each at 25°C and analyzed using the manufacturer's software (Dynamics, Wyatt Technology Corporation), while assuming particles with a spherical shape.

Preparation of LASV glycoprotein immune complex with LAVA01 Fab

To further stabilize the trimeric conformation, purified GPC-I53-50A was chemically cross-linked using glutaraldehyde. A concentration of 0.75 μg/mL GPC-I53-50A in PBS was mixed in a 1:1 volume ratio with PBS, 60 mM glutaraldehyde and incubated for 5 min at RT. The cross-linking reaction was then stopped by adding Tris, pH 7.4 to a final concentration of 75 mM, followed by an incubation step of 10 min at RT. Subsequently, GPC-I53-50A was dialysed twice to TBS and then twice to PBS using the Slide-A-Lyzer Mini dialysis devices with a 10 kDa molecular weight cutoff (Thermo Scientific). Finally, GPC-I53-50A was concentrated to at least 2 μg/mL using Vivaspin centrifugal filters 10 kDa molecular weight cutoff (Sigma-Aldrich). To generate complexes, 400 μg of LAVA01 antibody (as Fab fragment) was incubated with 200 μg of cross-linked GPC-I53-50A trimers for 1 h at room temperature. The complex was purified from excess/unassembled material using size-exclusion chromatography on a HiLoad® 16/600 Superdex® pg200 (Sigma-Aldrich) column running TBS buffer (Alfa Aesar), and concentrated to 3 mg/mL using the Amicon ultrafiltration units with a 10 kDa molecular weight cutoff (Sigma-Aldrich).

CryoEM grid preparation and imaging

For the preparation of cryoEM grids the GPC-I53-50NP sample (1) and the GPC-I53-50A immune complex with LAVA01 Fab (2) were concentrated to 2 mg/mL and 3 mg/mL, respectively. Vitrobot mark IV was used for the preparation of cryoEM grids. The settings were as follows: temperature inside the chamber was 10 °C, humidity was 100%, blotting force was 0, wait time was 10 s, blotting time was varied within a 3-6 s range. Lauryl maltose neopentyl glycol (LMNG) detergent at a final concentration of 0.005 mM was added to each sample and 3 μL of that solution was immediately loaded onto plasma-cleaned QuantiFoil R 2/1 (400-mesh; Thermo Scientific) and UltrAuFoil R 1.2/1.3 grids (300-mesh; Quantifoil Micro Tools GmbH). The plasma cleaning step was performed in the Solarus 950 plasma system (Gatan) with Ar/O_2 gas mix for 7 s. The sample was blotted off and the grids were plunge-frozen into liquid-nitrogen-cooled liquid ethane. Cryo-grids were imaged on an FEI Titan Krios (Thermo Scientific) microscope operating at 300 keV, equipped with a sample autoloader and the K2 Summit direct electron detector camera (Gatan). Exposure magnification was set to 29,000 and the resulting pixel size at the specimen plane was 1.03 Å. Automated image collection was performed using the Leginon software suite.[47] Data collection information can be found in Table S1.

CryoEM data processing

GPC-I53-50 nanoparticle. The GPC-I53-50 cryoEM data was processed as described previously.[60] Raw micrograph frames were aligned and dose-weighted using MotionCor2.[48] CTF parameters were estimated with GCTF.[49] Initial data processing steps were performed in cryoSPARC.v2.[46] 145,508 particles were template-picked, extracted and run through 2 rounds of 2D classification. 86,411 clean particles after 2D classification were subjected to Ab initio refinement in cryoSPARC to generate the starting model for 3D refinement steps (icosahedral symmetry was imposed). All further processing steps were performed in Relion/3.0.[50] 3D refinement with imposed icosahedral symmetry was run using the particles from the previous step. The refined particles were used for CTF refinement in Relion/3.0 (to improve the estimates for defocus and beam-tilt). To reconstruct the I53-50NP, particles from the previous step were 3D refined with a soft solvent mask around the nanoparticle core, masking out the density corresponding to the GPC trimers (icosahedral symmetry restraints imposed, local angular searches only). The resulting map was subsequently post-processed using the same soft mask to determine the B-factor (-202.1 $Å^2$) and the final resolution (3.67 Å). GPC trimers were connected to I53-50A components with a flexible linker, preventing a joint analysis with the I53-50NP core. We applied localized reconstruction v1.2.0[51] to extract GPC trimer subparticles connected to I53-50A. 20 trimer subparticles were extracted from each GPC-I53-50NP using the pre-defined vector settings for icosahedral symmetry (–vector 0.382,0,1, –length 180). The final number of extracted GPC subparticles was 1,728,220 (20 × 86,411). The subparticle subset was subjected to two rounds of 2D classification and three rounds of 3D classification to eliminate the low-resolution classes of subparticles as well as subparticles that had issues aligning due to the signal from the nanoparticle. The final GPC subset consisted of 124,891 subparticles and was subjected to 3D auto-refinement with C3 symmetry. A soft solvent mask around the GPC trimers was applied for the refinement and post-processing steps. Final map resolution was 3.97 Å and the estimated B-factor was -123.2 $Å^2$. The workflow and relevant data processing parameters are displayed in Figure S2.

CellPress
OPEN ACCESS

GPC-I53-50A + LAVA01 Fab. The frame alignment, dose weighting and CTF estimation steps were performed as described above for the nanoparticle dataset. 416,326 particles were templated picked from the micrographs in cryoSPARC.v2[46] and subjected to 2D classification to remove bad particles and monomers; LAVA01 Fab has been found to induce partial trimer disassembly. 93,965 particles were selected after the 2D step and subjected to 3D refinement step in Relion/3.0. A low-pass filtered map of GPC-I53-50A trimer without the LAVA01 Fab was used as a starting model for the initial 3D refinement. The resulting particles were then subjected to another round of 3D refinement with a soft solvent mask around the GPC + LAVA01 complex. This is done to mask out the signal from the flexibly linked I53-50A nanoparticle component. This solvent mask was used for all subsequent 3D refinement and classification steps. Additionally, the particle alignment was restricted to local angular searches only (–healpix_order 3, –auto_local_healpix_order 3) to complement the usage of the solvent mask and prevent major changes in particle orientation. C1 symmetry was used for all initial 3D steps. The dataset suffered from preferred orientation problems and it was dominated by top and bottom views of the complex. We applied an in house made program to gradually remove excess particles from over-populated views (based on Euler angle values). This was followed by iterative rounds of 3D classification (–skip_align, T=16, –sym=C1) and 3D refinement (–healpix_order 3, –auto_local_healpix_order 3, –sym=C1). The final subset consisting of 8,480 particles was 3D refined with C3 symmetry and post-processed using the GPC + LAVA01 solvent mask. Final map resolution of the GPC + LAVA01 complex was 4.41 Å and the estimated B-factor was -71.7 $Å^2$. The workflow and relevant data processing parameters are displayed in Figure S5.

Model building and refinement

The post-processed cryoEM maps corresponding to GPC trimer antigen, I53-50NP core and GPC + LAVA01 Fab complex were used for model building and refinement. As initial models we used the published I53-50NP core (PDB ID: 6P6F[29]) and GPC crystal structure (PDB ID: 5VK2[14]). Initial model for the LAVA01 Fab was generated using ABodyBuilder server.[52] Starting models were docked into the corresponding densities and relaxed using iterative rounds of manual refinement in Coot[53] and automated refinement in Rosetta.[61] Appropriate symmetry restraints (C3 for GPC and GPC + LAVA01 Fab complex and I for I53-50NP) were applied for automated refinement steps. EMRinger[54] and MolProbity[55] were used to evaluate the refined models and generate the statistics reported in Table S1. Due to the relatively low resolution of the GPC + LAVA01 Fab complex map the amino-acid side-chains of the LAVA01 Fab were not built past Cβ.

Rabbit immunizations

Female and naive New Zealand White rabbits (2.5–3 kg), were arbitrarily distributed in two groups of 6 rabbits and received an intramuscular immunization in each quadricep at weeks 0, 4, 16, and 28. Rabbits were immunized with either 30 μg of GPC-I53-50A or the equimolar amount presented on I53-50NPs (36 μg), both formulated in Squalene Emulsion adjuvant (Polymun, Klosterneuburg, Austria). Rabbits were sourced and housed at Covance Research Products Inc. (Denver, PA, USA) and immunizations were performed under compliance of all relevant ethical regulations for animal testing and research. The study received ethical approval from the Covance Institutional Animal Care and Use Committee with approval number C0096-019. Calculations of the dose were based on the peptidic molecular weight of the proteins which were obtained as described earlier. Bleeds were performed at weeks 0, 4, 6, 18 and 30. A larger blood draw at week 29 was also taken for isolation of peripheral blood mononuclear cells.

Guinea pig immunizations and challenge

Naive female Hartley guinea pigs (6 weeks old) were arbitrarily distributed in two groups of 5, after which they received either 30 μg of GPC-I53-50NP adjuvanted in Squalene Emulsion adjuvant (Polymun, Klosterneuburg, Austria) or Squalene Emulsion adjuvant alone (control animals), as an intramuscular injection distributed over both quadriceps at weeks 0, 4 and 16. On day 140 (week 20) guinea pigs were challenged intraperitoneally with a targeted dose of 1×10^4 PFU of guinea pig-adapted LASV Josiah strain.[35] Animals were then monitored at least daily for fever (via transponder chip), weight loss and clinical signs of disease. Animals were euthanized via CO_2 asphyxiation when humane euthanasia criteria were met, or at day 28 post-challenge. Humane euthanasia criteria were met when animals lost more than 20% of their starting weight or displayed clinical signs indicating that they had entered a moribund state (Table S5).

Focus-forming assay

Guinea pig serum was collected in serum separator tubes, centrifuged, and frozen. On the day of the assay, a tenfold dilution series was prepared and titrated on Vero (CCL-81) cells (ATCC) with a minimal essential medium overlay containing 0.5% carboxymethylcellulose, 2% fetal bovine serum, and 0.1% gentamicin in 48-well plates. Three days post-infection, plates were fixed in 10% neutral buffered formalin, and immunostained. Immunostaining was performed using anti-LASV mouse hyperimmune ascites fluid (a gift from Dr. Tom Ksiazek), and a goat anti-mouse secondary antibody (Seracare). Assays were developed using AEC chromogen (Enquire Bioreagents).

Serum antibody ELISA

Half-well 96-well plates were coated overnight with Galanthus nivalis lectin (Vector laboratories) at 20 μg/mL in 0.1 M $NaHCO_3$ pH 8.6. The next day the plates were washed three times with TBS and blocked for 30 min with Casein blocking buffer (Thermo Scientific). After washing the plates with TBS, 2 μg/mL of GPC-I53-dn5B diluted in Casein was added for 2 h. After a wash-step with TBS, three-fold dilutions of rabbit serum diluted 1:200 in TBS, 2% skimmed milk, 20% sheep serum were added to the plates.

After a 2 h incubation, the plates were washed with TBS and a 1:3000 dilution of HRP-labeled goat anti-rabbit IgG (Jackson Immunoresearch) in Casein was added for 1 h. The plates were then washed five times with TBS, 0.05% Tween-20 and developing solution (1% 3,3′,5,5′-tetranethylbenzidine (Sigma-Aldrich), 0.01% H_2O_2, 100 mM sodium acetate and 100 mM citric acid) was added. After 1.5 min the colorimetric detection reaction was stopped by adding 0.8 M H_2SO_4. All procedures were performed at RT. The midpoint binding titer (ED_{50}) was determined by calculating the dilution of serum that gave 50% of the maximal response from the sigmoidal binding curve. The same protocol was used for the guinea pig sera although a 1:100 start dilution was used, a 1:5000 dilution of HRP-labeled donkey anti-guinea pig IgG (Jackson Immunoresearch) and a colorimetric detection reaction of 2 min.

Luminex assay for GPC-specific IgM

GPC-I53-dn5B was covalently coupled to beads and Luminex assays were performed as described previously.[28] Briefly, Luminex Magplex beads were coupled using a two-step carbodiimide reaction with 1-Ethyl-3-(3-dimethylaminopropyl) carbodiimide and Sulfo-N-Hydroxysulfosuccinimide at a ratio of 19 μg GPC-I53-dn5B to 12,5 million beads. 15 beads per μL were incubated in a 1:1 ratio with 1:125000 diluted rabbit serum overnight at 4°C. Detection was performed with Goat-anti-rabbit IgM-PE (Southern Biotech). Read-out was performed on a Magpix instrument (Luminex). Resulting median fluorescence intensity values were corrected by subtraction of MFI values from buffer and beads only wells and baseline correction was performed by additional subtraction of the MFI value of each animal in the sample prior to immunization.

Antibody-dependent NK activation assay

High-binding ELISA microplates were precoated with GNL prior to the immobilization of 5 μg/mL GPC-I53-dn5B, incubated at 4°C overnight, washed with 1X Tris-Buffered Saline, and blocked with 1% BSA in PBS. PBMC were obtained from leukapheresis products of healthy donors by Ficoll-Paque density gradient following the manufacturer's protocol. NK cells were enriched by positive selection from human PBMCs using Miltenyi's CD56 MicroBeads following manufacturer's protocol and stimulated with 10 ng/mL IL-15 in Iscove's Modified Dulbecco's Medium supplemented with 10% FCS and 100 U/mL penicillin/streptomycin. Stimulated NK cells were incubated at 37°C overnight. On the day of the experiment, a 1:50 dilution of guinea pig serum in 1% BSA in PBS was added onto the well and incubated for 1 h at 37°C. NK cells were added onto the plate at 35,000 cells per well and incubated for 3 h at 37°C. After incubation, NK cells were transferred into a 96 well V-bottom microplate and stained with anti-CD16-PE (Biolegend) at a 1:1000 dilution (CD16 is a marker for NK cell activation[62]). As a positive control, phorbol myristate acetate (PMA) (50 ng/mL) and ionomycin (500 ng/mL) was added, and no serum wells was used as a negative control. Samples were read using a BD FACSymphony A1 Cell Analyzer.

Antibody-dependent cellular phagocytosis assay

This assay was performed as described previously.[63] In short, Fluorescent NeutrAvidin beads (Thermo Scientific) were incubated overnight at 4°C with biotinylated GPC-I53-50A protein (10μg/5μL beads suspension). After incubation, the beads were washed twice using PBS 2% bovine serum albumin (BSA). 50 μL of the bead suspension were placed in a V-bottom 96-well plate and incubated with 10-fold serial dilutions of guinea pig serum at a start dilution of 1:1000 in PBS 2%BSA. After 2 h at 37°C, the plates were washed and 5×10^4 THP-1 effector cells (monocytes; ATCC) were added to each well. To promote beads to cell contact, plates were quickly spun down before incubation for 5 h at 37°C. After incubation, the cells were washed and resuspended in PBS 2% FCS. Cells were analyzed by flow cytometry and the phagocytic activity was determined by the area under curve of the MFI (beads positive cells x mean MFI FITC).

B cell sorting

To generate probes for B cell sorting, biotinylated GPC-I53-50A from lineage II and IV as well as a biotinylated HCV E1E2-I53-50A (K.S. et al., unpublished data) were conjugated to the streptavidin-bound fluorophores AF647, BV421, and BB515, respectively. Conjugation was performed by incubating the biotinylated proteins for a minimum of 1 h at 4°C with the streptavidin-conjugates at a 1:2 protein to fluorochrome ratio. To saturate unconjugated streptavidin-fluorochrome complexes the fluorescent probes were next incubated for at least 10 min with 10mM biotin (GeneCopoeia) to saturate the unconjugated streptavidin-fluorochrome complexes. Week 29 rabbit PBMCs were then counted and $5\text{x}10^6$ cells were stained for 30 min at 4 with the fluorescent probes, a viability marker (LiveDead-eF780, eBiosciences), and a rabbit PE-conjugated anti-IgG marker (Biolegend). Prior to their acquisition on the FACS-ARIA-SORP 4 laser (BD-Biosciences), cells were washed twice with FACS buffer. Viable IgG+ B cells that were negative for HCV E1E2-I53-50A and showed dual staining for both GPC-I53-50A from lineage II and IV were sorted. Analysis was performed on FlowJo v.10.7.1.

Antibody cloning

First, a reverse transcription-PCR was performed to convert the mRNA of the lysed GPC-specific single B cells into cDNA. To do so, 6 μL of a reverse transcriptase-mix ((200 ng Random Hexamer Primers (Thermo Scientific), 2mM dNTP mix (New England Biolabs), 50U Superscript III RTase (Thermo Scientific) and MQ) was added to the lysed cells followed by a single cycle of 10 min at 42°C, 10 min at 25°C, 60 min at 50°C, 5 min at 95°C, and infinity 4°C. Next, the V(D)J variable regions of the cDNA were amplified by a series of PCR reactions which are distinct for heavy and light chains. The PCR mix consisted of MQ, 1x PCR reaction buffer, dNTPs (10 mM), HotStarTaq Plus polymerase (0.25 U; Qiagen), forward primer (25 mM) and reverse primer (25 mM).[64,65] For the first PCR reaction

CellPress
OPEN ACCESS

(PCR1), 13 μL of PCR mix was added to 2 μL of RT-PCR product and subjected to 5 min at 95°C, 50 cycles of [30 s at 94°C, 30 s at 58°C for light chain/48°C for heavy chain, and 1 min at 72°C], and 10 min at 72°C. Next, for PCR2, 13 μL of PCR mix was added to 2 μL of PCR1 product and subjected to a reaction of 5 min at 95°C, 50 cycles of [30 s at 94°C, 30 s at 55°C, and 1 min at 72°C], and 10 min at 72°C. Finally, 1 μL of PCR2 product was mixed with MQ, 1x Phusion PCR buffer, dNTPs (10 mM), forward primers (25 mM), reverse primers (25 mM), Phusion high fidelity polymerase (0.2 U; New England Biolabs) and subjected to a PCR reaction of 30 s at 98°C, 35 cycles of [5 s at 98°C, 15 s at 68°C, 20 s at 72°C], and 5 min at 72°C.

Gibson cloning was then used to integrate the amplified heavy and light chain V(D) J variable regions in mammalian cell expression vectors containing the rabbit constant regions.[65] This was done by mixing 1 μL of expression vector, 1 μL of PCR3 product, and 2 μL of home-made Gibson mix (T5 exonuclease (0.2U; New England Biolabs), Phusion polymerase (12.5U; New England Biolabs), Taq DNA ligase (2000U; New England Biolabs), Gibson reaction buffer (0.5 grams PEG-8000; Sigma Life Sciences), 1 M Tris/ HCl pH 7.5, 1 M MgCl2, 1 M DTT, 100 mM dNTPs, 50 mM NAD^+ (New England Biolabs), MQ) and incubating the mix for 60 min at 50°C.

Fab preparation

To generate LAVA01 Fab fragments, LAVA01 was subjected to a 5 h incubation at 37°C in PBS, 10 mM EDTA, 20 mM cysteine, pH 7.4 in the presence of 50 μL settled papain resin/mg of LAVA01. Next, Fc and non-digested mAbs were removed from the flow-through by a 2 h incubation at RT with 200 μL of protein A resin per mg of initial mAb (Thermo Scientific). Finally, the flow-through containing Fab fragments was buffer exchanged to TBS using Vivaspin filters with a 10 kDa molecular weight cutoff (Sigma-Aldrich).

Monoclonal antibody ELISA

A 2 μg/mL concentration of GPC-I53-dn5B diluted in Casein blocking buffer (Thermo Scientific) was added for 2 h on high-binding 96-well plates (Greiner). Four-fold serial dilutions of mAbs diluted to 2.5 μg/mL in Casein were then added for 2 h. A 1:3000 dilution of HRP-labeled goat anti-rabbit IgG (Jackson Immunoresearch) in Casein was added for 1 h. Up to now, between each step, plates were washed three times with TBS. Next, plates were washed five times with TBS, 0.05% Tween-20. Colorimetric detection was performed as described above in Serum antibody ELISA. All procedures were performed at RT. The midpoint binding concentration (IC_{50}) was determined by calculating the concentration of mAb that gave 50% of the maximal response from the sigmoidal binding curve.

Generation of LASV pseudovirus

LASV pseudoviruses were generated as described previously.[19] HEK 293T cells maintained in Dulbecco's Modified Eagle's Medium (DMEM; Gibco) supplemented with 10% FCS, penicillin (100 U/mL), and streptomycin (100 μg/mL) were plated in 6-well plates and grown overnight to 80% confluence. The next day for each well a 1:25 dilution of Fugene in OptiMEM (final volume 250 μL; Gibco) was mixed with 0.6 μg of a full-length GPC expression plasmid and 2.4 μg of SG3Δenv (a plasmid encoding the envelope-deficient core of HIV-1) diluted in OptiMEM (final volume 250 μL; Gibco). After a 20 min incubation at RT, the mix was added to the well. After 72 h the supernatant was harvested, sterile filtered with a 0.2 μm filter, aliquoted, and stored at -80°C. To determine virus titers, a $TCID_{50}$ experiment was performed. TZM-bl cells were maintained in DMEM, supplemented with 10% FCS, penicillin (100 U/mL), and streptomycin (100 μg/mL) and grown overnight in a 96-well plate to a confluency of 70-80%. The next day, pseudovirus stocks were serially diluted in triplicate, incubated at RT for 1 h, and added to the TZM-bl cells. Shortly prior to addition of pseudovirus dilutions to the cells, the medium was supplemented with DEAE-dextran and Saquinavir (Sigma-Aldrich), to a final concentration of 40 μg/mL and 400 nM, respectively. Cells were then incubated at 37°C for 72 h after which they were lysed for 20 min on a shaker platform at RT by addition of Reporter Lysis Buffer (Promega). Luciferase signal was determined from cell lysates by adding the Bright-Glo Luciferase buffer (Promega) and subsequent analysis using a Glomax plate reader. The pseudovirus input for neutralization assays was determined as the dilution that gave luciferase counts of 500,000 (i.e. >10x above background).

Pseudovirus neutralization assay

TZM-bl cells maintained in DMEM (Gibco), supplemented with 10% FCS, penicillin (100 U/mL), and streptomycin (100 μg/mL) were grown overnight in a 96-well plate to a confluency of 70-80%. The next day serial dilutions of rabbit serum or mAbs were incubated for 1 h with pseudovirus. All dilutions were performed in DMEM (Gibco), supplemented with 10% FCS, penicillin (100 U/mL), and streptomycin (100 μg/mL). The starting dilution of rabbit serum was 1:20, which was serially diluted three-fold. As guinea pig serum gave high background neutralization, IgG had to be purified from the serum as described previously.[66] The purified IgG, diluted to the same volume as the initial serum in PBS, was diluted three-fold from a 1:20 starting dilution. For mAbs the starting concentration was 20 μg/mL or 100 μg/mL and serial dilutions were five-fold. The virus:serum/IgG/mAb mix was added to TZM-bl cells which were supplemented prior with DEAE-dextran and Saquinavir (Sigma-Aldrich), as described above. After a 72 h incubation, plates were lysed and luciferase was measured as described above. Luciferase counts were normalized to those obtained for cells infected with pseudovirus without the presence of serum/IgG/mAbs. ID_{50} and IC_{50} values were determined as the dilution/concentration where 50% inhibition of infectivity was achieved.

Neutralization assay using authentic LASV

Neutralization assays with authentic LASV (strain Josiah, lineage IV) were performed in the BSL-4 laboratory of the Institute of Virology, Philipps University Marburg, Germany. Rabbit sera were complement inactivated for 30 min at 56 °C and diluted in a

two-fold dilution series with a starting dilution of 16. Diluted sera, LAVA01, 19.7E or 37.7H (initial concentration of mAbs was 100 μg/mL), were mixed with 100 $TCID_{50}$ virus and incubated for 60 min at 37 °C. Following incubation, Vero E6 cell (ATCC) suspension was added and plates were then incubated at 37°C with 5% CO_2. Cytopathic effects (CPE) were evaluated at seven days post infection and neutralization titers were calculated as geometric mean titer (GMT) of four replicates.

QUANTIFICATION AND STATISTICAL ANALYSIS

The number of animals (*n*), the statistical test used, the definition of center, and dispersion measures (if applicable) can be found in the figure legends. Due to the small sample size and the resulting inability to perform D'Agostino-Pearson tests for normality, two-tailed Mann-Whitney *U*-tests were performed to compare between two independent data sets. For the same reasons, non-parametric tests were used to analyze correlations (two-tailed Spearman's rank test) and survival (log-rank test). Statistical differences were considered significant for P values < 0.05. Dose-response binding/neutralization curves were subjected to non-linear regression analysis. All statistical analyses as well as calculations of ED_{50}, ID_{50}, and IC_{50} values were performed using Graphpad Prism 8.0.

Cell Host & Microbe, Volume *30*

Supplemental information

Lassa virus glycoprotein nanoparticles elicit neutralizing antibody responses and protection

Philip J.M. Brouwer, Aleksandar Antanasijevic, Adam J. Ronk, Helena Müller-Kräuter, Yasunori Watanabe, Mathieu Claireaux, Hailee R. Perrett, Tom P.L. Bijl, Marloes Grobben, Jeffrey C. Umotoy, Angela I. Schriek, Judith A. Burger, Khadija Tejjani, Nicole M. Lloyd, Thijs H. Steijaert, Marlies M. van Haaren, Kwinten Sliepen, Steven W. de Taeye, Marit J. van Gils, Max Crispin, Thomas Strecker, Alexander Bukreyev, Andrew B. Ward, and Rogier W. Sanders

Table S1. CryoEM data collection information, model building and refinement information, related to figure 3 and 5

	GPC-I53-50 nanoparticle	GPC-I53-50A + LAVA01 Fab		GPC	I53-50 nanoparticle (full assembly)	GPC-I53-50A + LAVA01 Fab
Microscope	Titan Krios	Titan Krios	Final map resolution (Å)	3.97	3.67	4.41
Voltage (kV)	300	300	EMDB ID	EMD-25107	EMD-25108	EMD-25109
Detector	Gatan K2 Summit	Gatan K2 Summit	Residues	1089	21060	1728
Recording mode	Counting	Counting	Amino-acids	1014	21060	1641
Magnification	29,000	29,000	Carbohydrates	75	0	87
Movie micrograph pixel size	1.03	1.03	RMSD Bond Length (4σ)	0.023	0.020	0.021
Dose rate ($e^-/Å^2/s$)	4.8	5.3	RMSD Bond Angles (4σ)	1.733	1.688	1.971
No. of frames per movie micrograph	42	38	Ramachandran			
Frame exposure time (ms)	250	250	Outliers (%)	0.0	0.0	0.0
Movie micrograph exposure time (s)	10.5	9.5	Allowed (%)	1.8	2.3	1.9
Total dose ($e^-/Å^2$)	50.4	50.3	Favored (%)	98.2	97.7	98.1
Grid Type	QuantiFoil R 2/1	UltrAuFoil R 1.2/1.3	Rotamer Outliers (%)	0.0	0.0	0.0
Under focus range (µm)	0.6 - 1.6	0.6 - 1.6	Clash Score	1.47	0.19	1.10
Number of movie micrographs	2,337	3,098	MolProbity Score	0.88	0.64	0.82
			EMRinger Score	2.57	2.81	1.37
			PDB ID	7SGD	7SGE	7SGF

Table S2. Midpoint pseudovirus neutralization titers from rabbits that received GPC-I53-50A or GPC-I53-50NPs, tested against a panel of LASV-pseudoviruses, related to figure 4

ID_{50} values, i.e. the serum dilution at which infectivity was inhibited by 50%, are shown and color coded: white = no neutralization, ID_{50} < 20; yellow = weak neutralization, 20 > ID_{50} >100; orange = moderate neutralization, 101 > ID_{50} > 1000; red = strong neutralization, ID_{50} > 1000. BG505 = HIV pseudovirus (negative control).

Rabbit immunization

		Week 0	Week 4	Week 6	Week 18		Week 30				
		Autologous	Autologous	Autologous	Control	Autologous	Control	Autologous	Heterologous		
	Strain	Josiah	Josiah	Josiah	BG505	Josiah	BG505	Josiah	NIG08-A41	LassaCSF	Bamba
	Lineage	IV	IV	IV	-	IV	-	IV	II	III	V
Immunogen	ID										
GPCysR4-I53-50A	187	<20	<20	<20	<20	48	<20	580	275	199	<20
	188	<20	<20	<20	<20	<20	<20	<20	<20	<20	<20
	189	<20	<20	<20	<20	1518	<20	2565	411	622	<20
	190	<20	<20	<20	<20	<20	<20	<20	<20	<20	<20
	191	<20	<20	<20	<20	<20	<20	<20	<20	<20	<20
	192	<20	<20	<20	<20	<20	<20	<20	<20	<20	<20
GPCysR4-I53-50NP	193	<20	<20	<20	<20	38	<20	51	30	24	<20
	194	<20	<20	<20	<20	623	<20	2749	1733	1151	41
	195	<20	<20	<20	<20	39	<20	93	45	42	41
	196	<20	<20	<20	<20	121	<20	413	42	52	37
	197	<20	<20	<20	<20	58	<20	50	26	26	<20
	198	<20	<20	<20	<20	<20	<20	<20	<20	21	<20

Table S3. Endpoint neutralization titers from rabbits that received GPC-I53-50A or GPC-I53-50NPs, tested against authentic LASV, related to figure 4

The virus neutralization titer is calculated as the geometric mean titers (GMT) of the reciprocal value of the last serum dilution at which inhibition of the cytopathic effect on infected Vero E6 cells is detectable. The initial dilution was 1:16 so a titer of 8 was noted when no inhibition was observed.

Rabbit immunization

		Week 30
		Autologous
	Strain	Josiah
	Clade	IV
Immunogen	ID	
GPCysR4-I53-50A	187	38
	188	8
	189	23
	190	8
	191	8
	192	8
GPCysR4-I53-50NP	193	8
	194	32
	195	11
	196	8
	197	11
	198	8

Table S4. Midpoint pseudovirus neutralization titers from guinea pigs that received GPC-I53-50NPs, tested against autologous LASV-pseudovirus, related to figure 4
ID_{50} values, i.e. the purified IgG dilution at which infectivity was inhibited by 50%, are shown and color coded: white = no neutralization, ID_{50} < 20.

Guinea pig immunization

		Week 18
		Autologous
	Strain	Josiah
	Clade	IV
Immunogen	ID	
GPCysR4-I53-50NP	NP-V1	<20
	NP-V2	<20
	NP-V3	<20
	NP-V4	<20
	NP-V5	<20

Table S5. Scoring scheme to assess the health of guinea pigs post-challenge, related to figure 4

**Score (1-4)**	**Description of Animal**
**1**	Healthy
**2**	Ruffled fur and Hunched posture (Triggers 2nd observation)
**3**	A score of 2 plus 1 additional clinical sign such as, Lethargy, Orbital tightening, and/or >15% weight loss (Triggers 3rd observation)
**4**	A score of 3 plus 1 additional clinical sign such as, Refusal to stampede, or any neurologic signs (rectal prolapse, seizures, tremors, head tilt, paralysis, etc.) OR >20% weight loss - Immediate Euthanasia

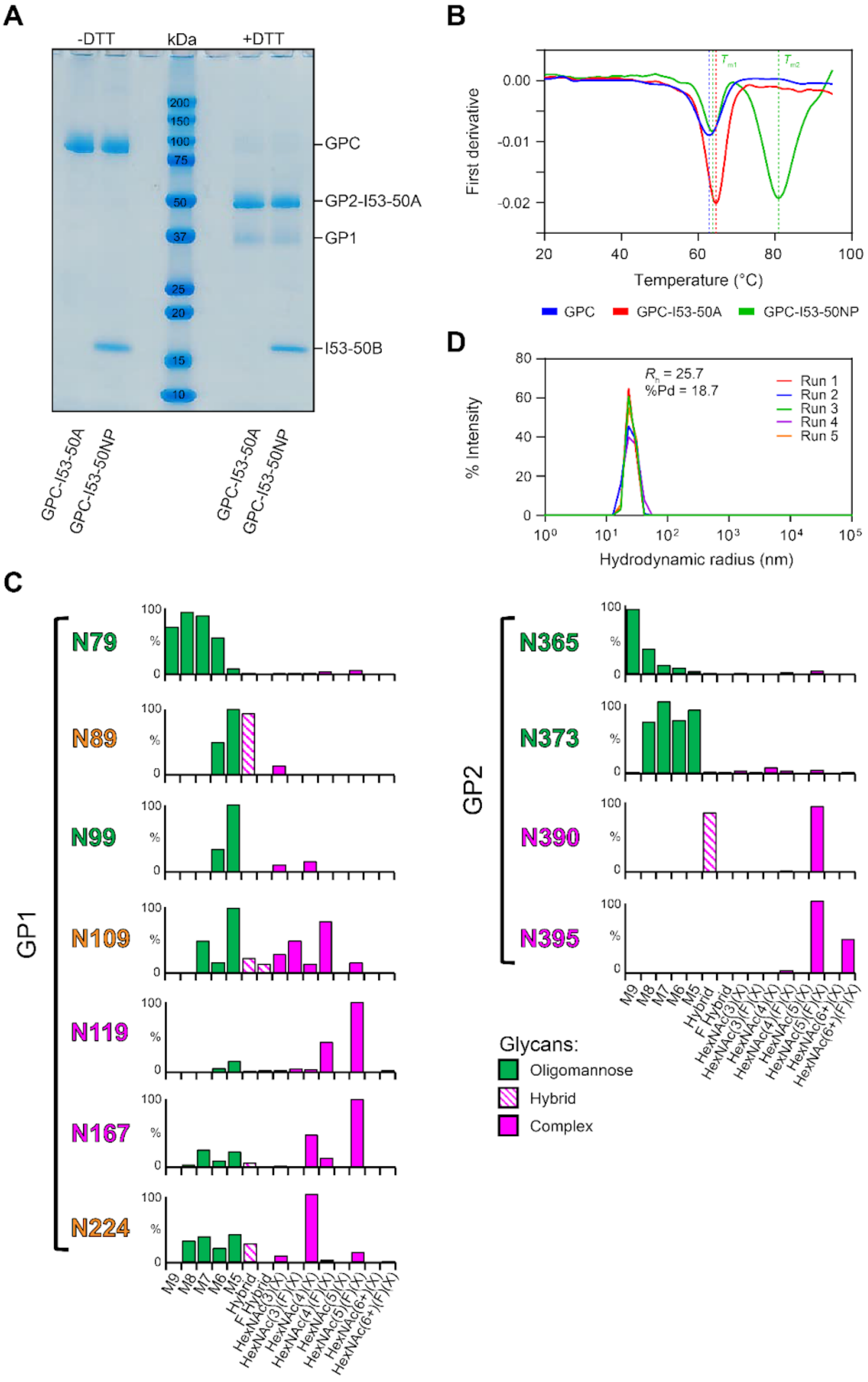

A
-DTT
kDa
+DTT
200
150
100
75
50
37
25
20
15
10
GPC
GP2-I53-50A
GP1
I53-50B
GPC-I53-50A
GPC-I53-50NP
GPC-I53-50A
GPC-I53-50NP
B
First derivative
Temperature (°C)
GPC
GPC-I53-50A
GPC-I53-50NP
D
% Intensity
Hydrodynamic radius (nm)
R_h = 25.7
%Pd = 18.7
Run 1
Run 2
Run 3
Run 4
Run 5
C
GP1
N79
N89
N99
N109
N119
N167
N224
GP2
N365
N373
N390
N395
M9
M8
M7
M6
M5
Hybrid
F Hybrid
HexNAc(3)(X)
HexNAc(3)(F)(X)
HexNAc(4)(X)
HexNAc(4)(F)(X)
HexNAc(5)(X)
HexNAc(5)(F)(X)
HexNAc(6+)(X)
HexNAc(6+)(F)(X)
Glycans:
Oligomannose
Hybrid
Complex

Figure S1. Biophysical characterization of GPC-I53-50A and GPC-I53-50NPs, related to figure 1 and 2

(A) SDS-PAGE analysis of GPC-I53-50A and GPC-I53-50NP in the absence (-DTT) and presence (+DTT) of dithiothreitol (DTT). (B) NanoDSF curves of GPC, GPC-I53-50A, and GPC-I53-50NP. The dotted lines indicate the melting tempera-tures (T_m), which is defined as the temperature where 50% of the protein is unfolded. GPC-I53-50NP has two melting temperatures; one for unfolding of GPC (T_{m1}) and one for the I53-50NP core (T_{m2}). Representative melting curve of at least two technical replicates is shown. (C) Site-specific glycan distribution of N-linked glycans on GPC-I53-50A as determined by LC-MS. The bar graphs represent the relative quantities of each glycan group with oligomannose-type glycan series (M9 to M5; Man9GlcNAc2 to Man5GlcNAc2) (green), a fucosylated and fucosylated hybrid glycans (Hybrid & F Hybrid) (dashed pink) and complex glycans grouped according to fucosylation and the number of antennae (HexNAc(3)(X) to HexNAc(6+)(F)(X)) (pink). (D) DLS data of GPC-I53-50NPs with the average polydispersity (%Pd) and hydrodynamic radius (R_h) of 5 runs shown. An overlay of each run is shown. A %Pd < 15 is considered a monodisperse population.

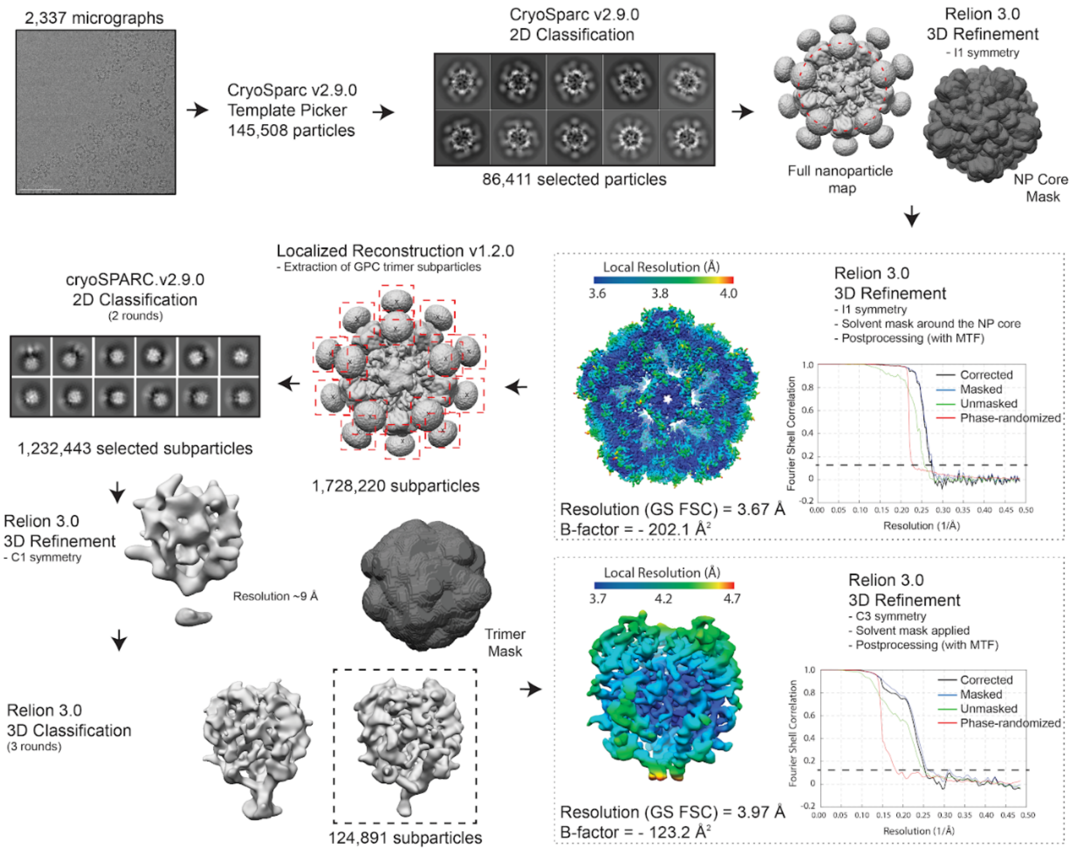

Figure S2. CryoEM data processing workflow for GPC-I53-50 nanoparticles, related to figure 3

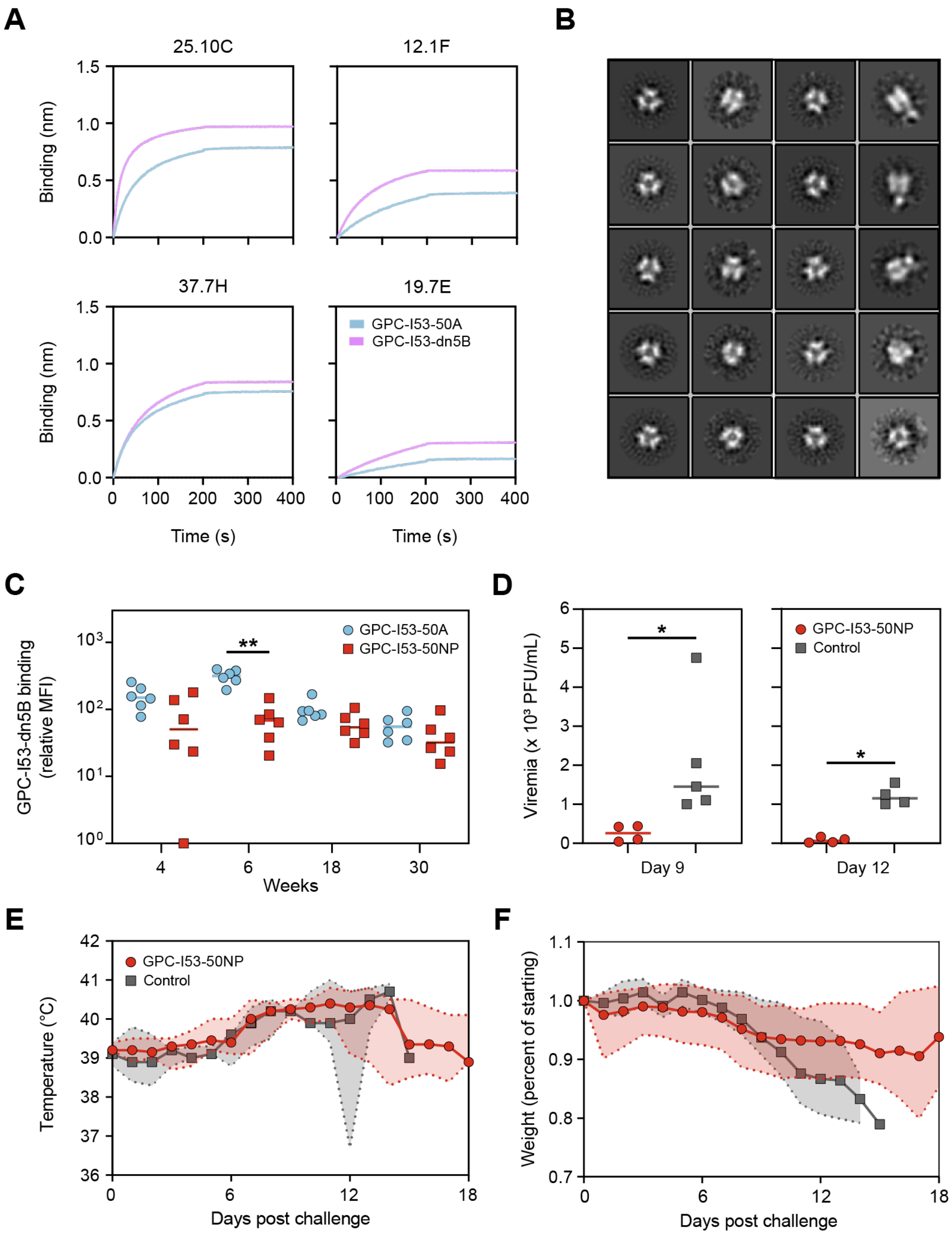

Figure S3. Antigenic and structural characterization of GPC-dn5B, IgM responses in vaccinated rabbits, and viremia, body temperature and weight alteration in vaccinated and control guinea pigs after challenge, related to figure 4

(A) Sensorgrams from BLI experiments comparing the binding to GPC-specific mAbs 25.10C, 12.1F, 37.7H, and 19.7E by GPC-I53-50A and GPC-dn5B. (B) 2D-class averages from nsEM with GPC-dn5B. (C) Relative mean fluorescence intensity (MFI) of serum IgM binding to GPC-I53-dn5B measured with a Luminex-based serology assay. Horizontal bars represent medians. Statistical differences between two groups (n = 6 rabbits) were determined using two-tailed Mann–Whitney U-tests (**$p < 0.01$). (D) Median RNA viral loads in vaccinated and control guinea pigs after challenge at day 9 and day 12. Statistical differences between two groups (day 9: n = 4 for vaccinated, n = 5 for controls; day 12: n = 4 for vaccinated and controls) were determined using two-tailed Mann–Whitney U-tests (*$p < 0.05$). (E) Median body

temperature over time of vaccinated and control guinea pigs after challenge. The shaded area indicates the range. (F) Median weight alteration in vaccinated and control guinea pigs over time after challenge. The shaded area indicates the range. The same color coding was used as in panel E.

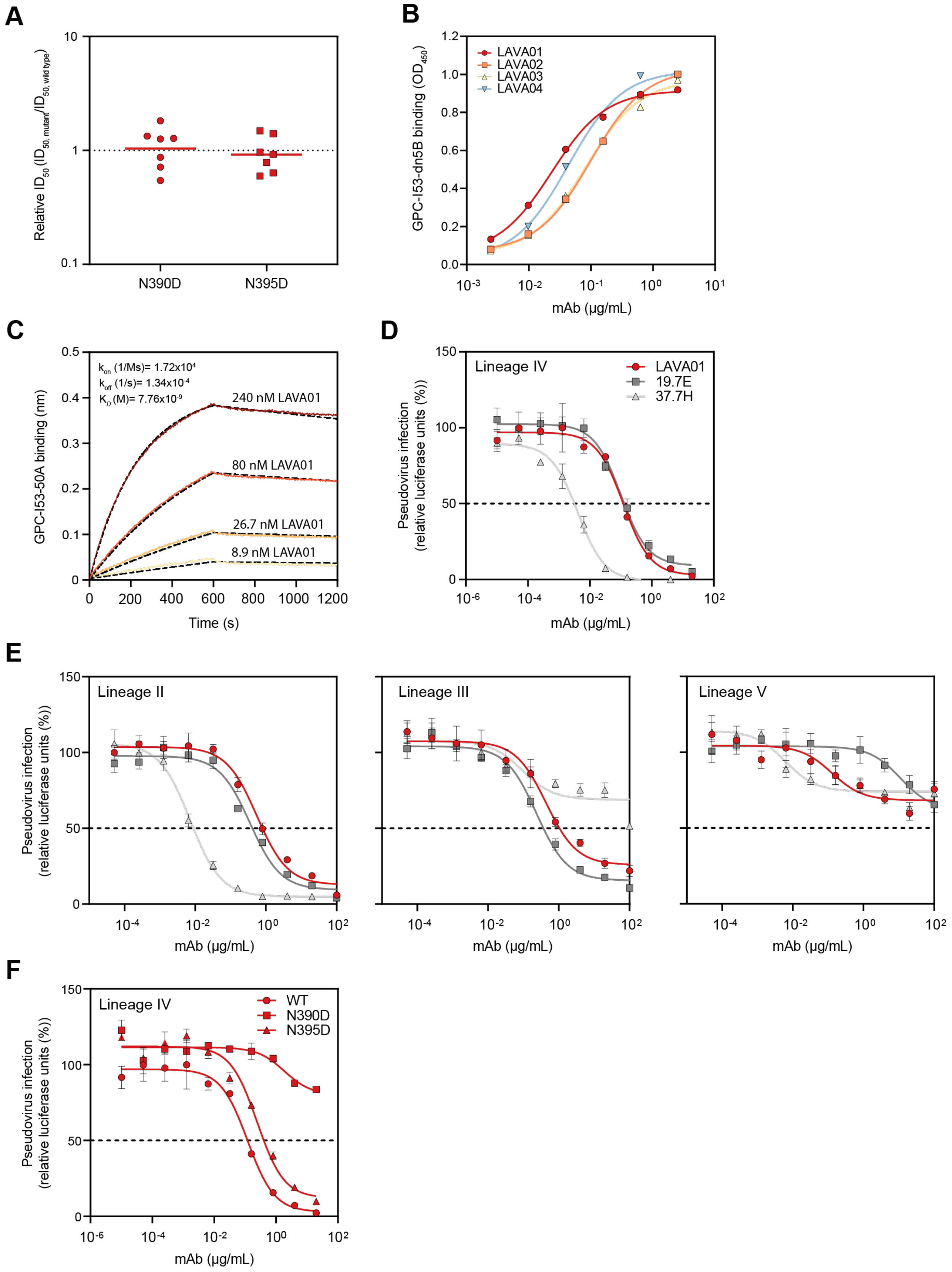
A
Relative ID50 (ID50, mutant/ID50, wild type)
10
1
0.1
N390D
N395D
B
GPC-I53-dn5B binding (OD450)
1.2
1.0
0.8
0.6
0.4
0.2
0.0
LAVA01
LAVA02
LAVA03
LAVA04
10-3
10-2
10-1
100
101
mAb (µg/mL)
C
GPC-I53-50A binding (nm)
kon (1/Ms)= 1.72x104
koff (1/s)= 1.34x10-4
KD (M)= 7.76x10-9
240 nM LAVA01
80 nM LAVA01
26.7 nM LAVA01
8.9 nM LAVA01
0.5
0.4
0.3
0.2
0.1
0
0
200
400
600
800
1000
1200
Time (s)
D
Pseudovirus infection
(relative luciferase units (%))
Lineage IV
LAVA01
19.7E
37.7H
150
100
50
0
10-6
10-4
10-2
100
102
mAb (µg/mL)
E
Lineage II
Lineage III
Lineage V
10-4
10-2
100
102
F
Lineage IV
WT
N390D
N395D

Figure S4. Dependency of rabbit serum and LAVA01 neutralization on N390 and N395 glycans, binding of isolated mAbs to GPC, kinetics of LAVA01 binding, and comparison of LASV pseudovirus neutralization between LAVA01, 19.7E and 37.7H, related to figure 5

(A) Relative ID_{50} for the N390D and N395D pseudovirus mutant relative to the parental lineage IV (Josiah) pseudovirus for all rabbit sera that showed an autologous neutralization ID_{50} titer >20. The dotted line indicates a RID_{50} of 1, i.e. the mutation has no effect on neutralization. (B) Binding of LAVA01-LAVA04 to GPC-dn5B as determined by ELISA. (C) Representative sensorgams from a BLI experiment to determine the binding kinetics of LAVA01 to GPC-I53-50A. Fit curves used for calculating kinetics values shown as black lines. (D) Neutralization of lineage IV (Josiah) pseudovirus by LAVA01, 19.7E, and 37.7H, as indicated in the legend. (E) Neutralization of lineage II (NIG08-A41; left), III (CSF; middle), and V (Bamba; right) pseudovirus by LAVA01, 19.7E, and 37.7H. The mean and SEM of at least two technical replicates are shown. (F) Neutralization curves of N390D or N395D pseudovirus mutants compared to the parental lineage IV pseudovirus (WT) by LAVA01. (D)-(F) The dotted line indicates 50% neutralization.

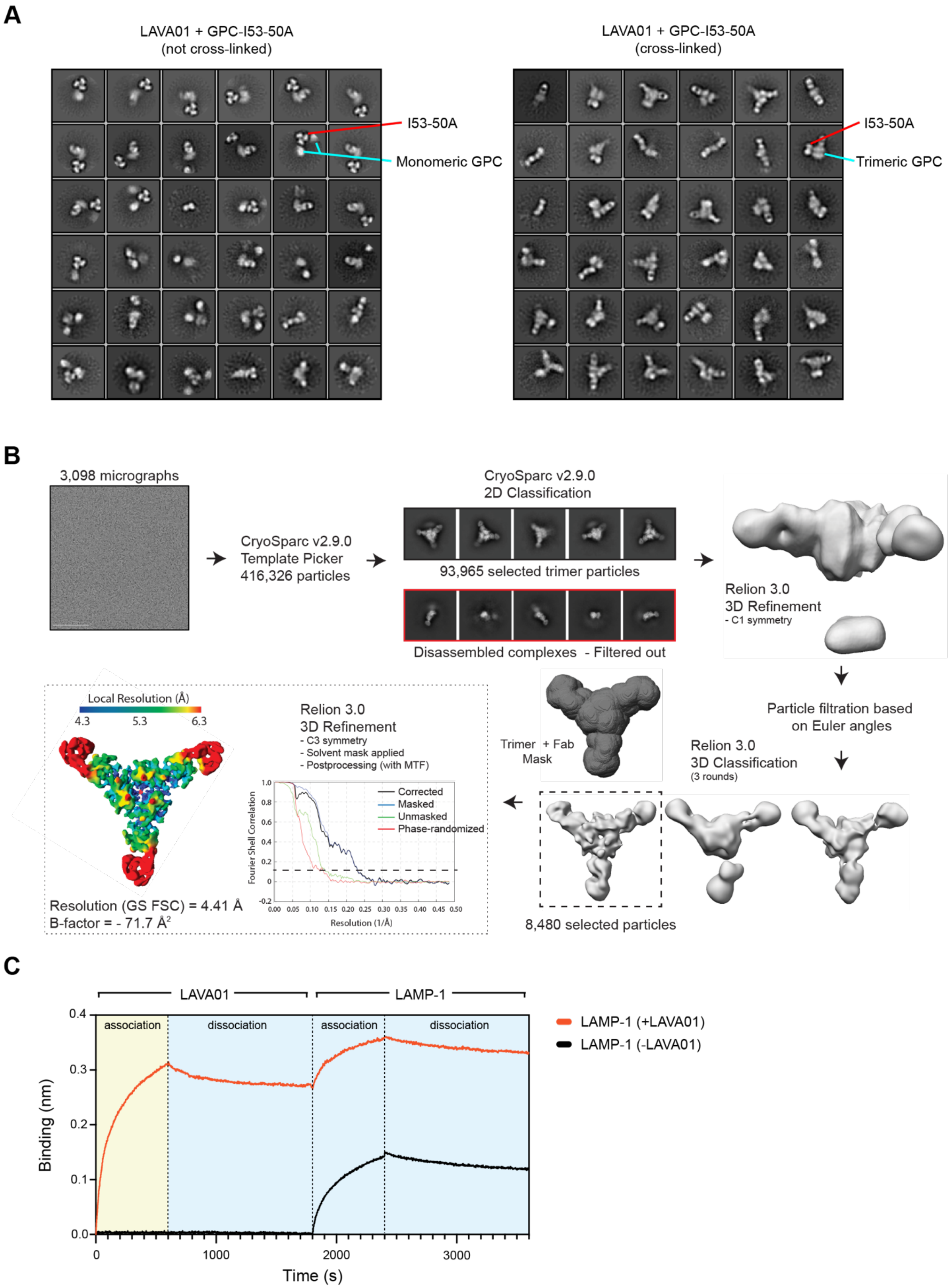
A
LAVA01 + GPC-I53-50A
(not cross-linked)
I53-50A
Monomeric GPC
LAVA01 + GPC-I53-50A
(cross-linked)
I53-50A
Trimeric GPC
B
3,098 micrographs
CryoSparc v2.9.0
Template Picker
416,326 particles
CryoSparc v2.9.0
2D Classification
93,965 selected trimer particles
Disassembled complexes - Filtered out
Relion 3.0
3D Refinement
- C1 symmetry
Particle filtration based
on Euler angles
Relion 3.0
3D Classification
(3 rounds)
Trimer + Fab
Mask
8,480 selected particles
Local Resolution (Å)
4.3
5.3
6.3
Relion 3.0
3D Refinement
- C3 symmetry
- Solvent mask applied
- Postprocessing (with MTF)
Corrected
Masked
Unmasked
Phase-randomized
Fourier Shell Correlation
Resolution (1/Å)
Resolution (GS FSC) = 4.41 Å
B-factor = - 71.7 Å^2
C
LAVA01
LAMP-1
association
dissociation
association
dissociation
LAMP-1 (+LAVA01)
LAMP-1 (-LAVA01)
Binding (nm)
Time (s)

Figure S5. CryoEM data processing workflow for complexes of chemically cross-linked GPC-I53-50A with LAVA01, related to figure 5

(A) 2D-class averages from nsEM with complexes of LAVA01 with GPC-I53-50A (left) and chemically cross-linked GPC-I53-50A (right). The I53-50A component and GPC are indicated. (B) CryoEM data processing workflow for complexes of LAVA01 with chemically cross-linked GPC-I53-50A. (C) Representative sensorgram from BLI experiments showing the binding of recombinant LAMP-1 ectodomain to GPC-I53-50A, complexed (+LAVA01) or uncomplexed (-LAVA01) with LAVA01. Loading of the sensor with GPC-I53-50A is not shown. The yellow background represents binding conditions at pH 8.0. The light blue background represents binding conditions at pH 5.0.
